# No genetic evidence for involvement of Deltaretroviruses in adult patients with precursor and mature T-cell neoplasms

**DOI:** 10.1186/1742-4690-4-11

**Published:** 2007-02-07

**Authors:** Thomas Burmeister, Stefan Schwartz, Michael Hummel, Dieter Hoelzer, Eckhard Thiel

**Affiliations:** 1 Charité Universitätsmedizin Berlin, Campus Benjamin Franklin, Medizinische Klinik III, Hindenburgdamm 30, 12200 Berlin, Germany; 2 Charité Universitätsmedizin Berlin, Campus Benjamin Franklin, Institut für Pathologie, Hindenburgdamm 30, 12200 Berlin, Germany; 3 Johann Wolfgang Goethe-Universität, Medizinische Klinik III, Theodor Stern-Kai 7, 60590 Frankfurt/Main, Germany

## Abstract

**Background:**

The Deltaretrovirus genus comprises viruses that infect humans (HTLV), various simian species (STLV) and cattle (BLV). HTLV-I is the main causative agent in adult T-cell leukemia in endemic areas and some of the simian T-cell lymphotropic viruses have been implicated in the induction of malignant lymphomas in their hosts. BLV causes enzootic bovine leukosis in infected cattle or sheep. During the past few years several new Deltaretrovirus isolates have been described in various primate species. Two new HTLV-like viruses in humans have recently been identified and provisionally termed HTLV-III and HTLV-IV. In order to identify a broad spectrum of Deltaretroviruses by a single PCR approach we have established a novel consensus PCR based on nucleotide sequence data obtained from 42 complete virus isolates (HTLV-I/-II, STLV-I/-II/-III, BLV). The primer sequences were based on highly interspecies-conserved virus genome regions. We used this PCR to detect Deltaretroviruses in samples from adult patients with a variety of rare T-cell neoplasms in Germany.

**Results:**

The sensitivity of the consensus PCR was at least between 10^-2 ^and 10^-3 ^with 100% specificity as demonstrated by serial dilutions of cell lines infected with either HTLV-I, HTLV-II or BLV. Fifty acute T-cell lymphoblastic leukemia (T-ALL) samples and 33 samples from patients with various rare mature T-cell neoplasms (T-PLL, Sézary syndrome and other T-NHL) were subsequently investigated. There were no cases with HTLV-I, HTLV-II or any other Deltaretroviruses.

**Conclusion:**

The results rule out a significant involvement of HTLV-I or HTLV-II in these disease entities and show that other related Deltaretroviruses are not likely to be involved. The newly established Deltaretrovirus PCR may be a useful tool for identifying new Deltaretroviruses.

## Background

Retroviruses are the main etiologic agents in a variety of malignant diseases in animals [1]. Bovine leukemia virus (BLV) was the first Deltaretrovirus to be discovered in 1969 by electron microscopy [2], but it was not until 1985 that the first complete nucleotide sequence of an isolate was reported [3]. Since the discovery of HTLV-I [4] and HTLV-II [5] and their closely related simian counterparts STLV-I [6] and STLV-II [7] several Deltaretrovirus isolates have been described in various non-human primate species. In 1994, a third simian Deltaretrovirus, later designated as STLV-III, was identified in a Hamadryas Baboon (*Papio Hamadryas*) [8-10]. Until recently no human counterparts of STLV-III were known, but in 2005 two independent research groups described human isolates that showed high homology to STLV-III and were considered to be HTLV-III isolates [11,12]. Moreover, a fourth Deltaretrovirus was identified in a single human individual from the Rain Forest in Cameroon. It did not show sufficient homology to be classified as primate T-cell lymphotropic virus (PTLV) type I, II or III and was thus considered to be a species representative of a hitherto unknown putative PTLV-IV virus group [12].

HTLV-I and STLV-I are etiologically linked to the induction of certain T-cell lymphomas/leukemias in their hosts [13,14]. The oncogenic action of the virus is mediated by the viral *tax *and *rex *genes that act as transcription factors, thereby promoting cell growth and malignant transformation However, the etiology of many human malignant T-cell and T-/NK-cell disorders is still not well understood. On the other hand a great deal of knowledge has been gained in the last years on the molecular biology of Deltaretroviruses, since a large number of new isolates have been described. To investigate the possible involvement of Deltaretroviruses in various human T-cell neoplasms, we have constructed a novel Deltaretrovirus consensus PCR based on nucleotide sequence alignments of all 42 complete Deltaretrovirus isolates published to date. Highly conserved virus genome regions were identified that allowed the construction of a generic PCR, capable of detecting all known Deltaretroviruses.

## Results

**Figure 1:**
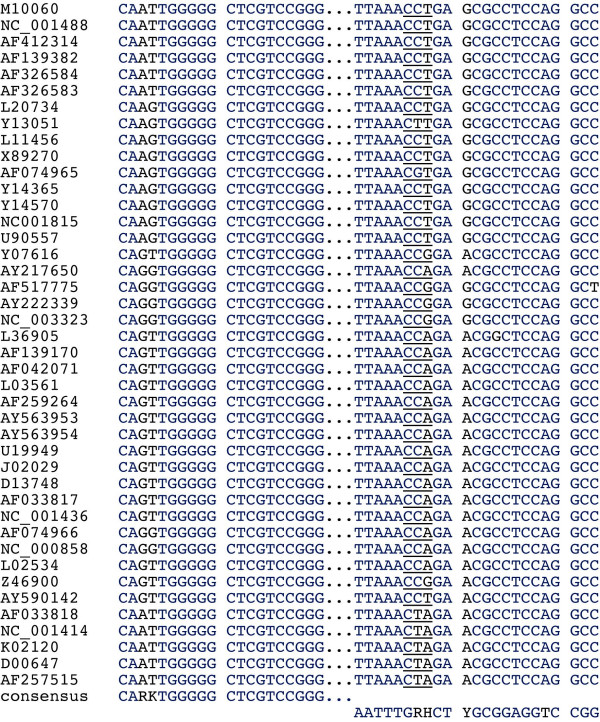
PCR primer regions with consensus primers. The accession numbers of the different isolates are given on the left. The first region corresponds to the proline tRNA binding site; the start of the *pol *ORF is underlined in the second region (site of ribosomal frameshift).

**Figure 2:**
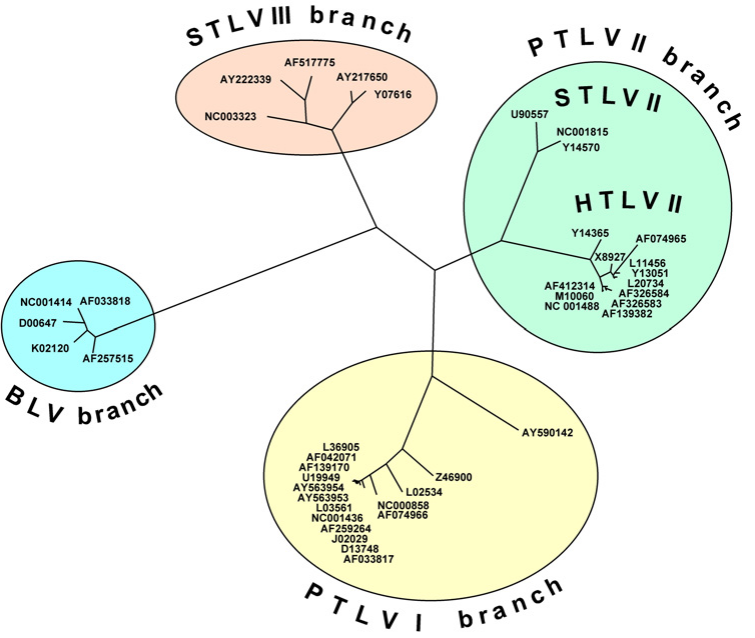
Phylogenetic tree based on the nucleotide sequence alignment of the amplified region. The recently described HTLV-III and HTLV-IV isolates are not included since no complete isolates have been published yet. The tree is not intended to set up a phylogeny but to illustrate the genetic relationships between the isolates.

**Figure 3:**
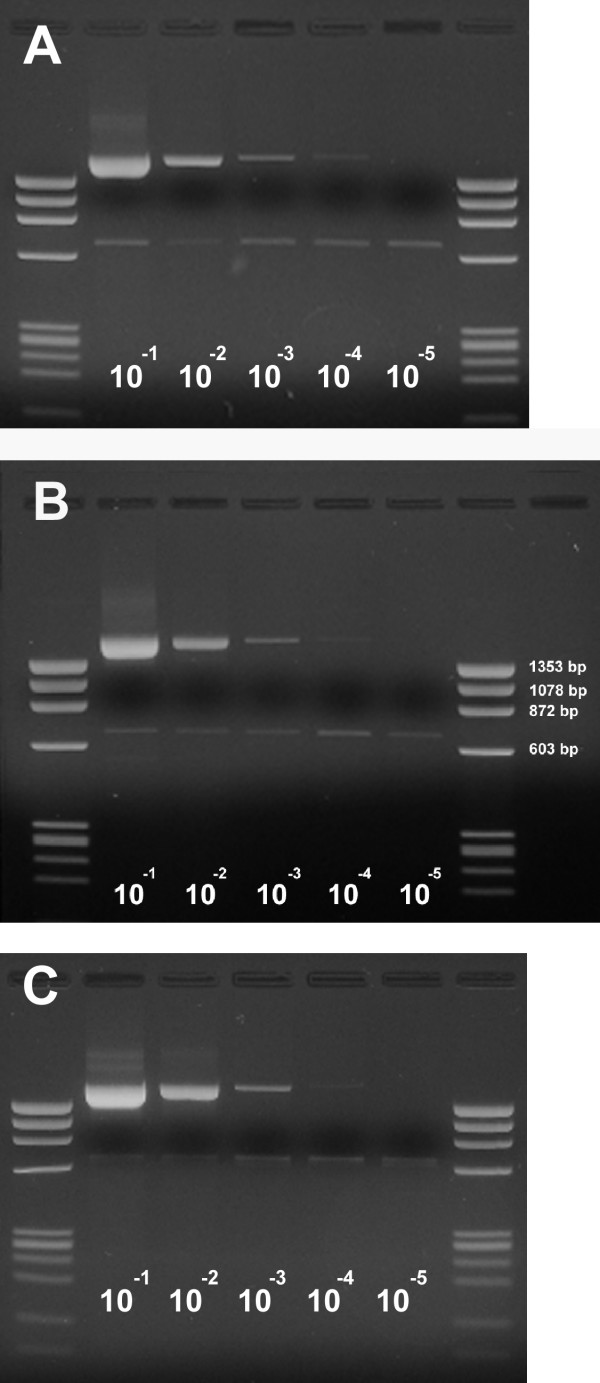
Deltaretrovirus PCR tested in serial dilutions of cell lines. Cell line DNA was diluted in genomic DNA from healthy individuals. **A**: cell line *Mo T *(HTLV-II-infected), **B**: cell line *MJ *(HTLV-I-infected), **C**: cell line *BL3.1 *(BLV-infected). First and last lane in every gel: φX174/*Hae *III size standard (QIAGEN, Hilden/Germany). No cell line harboring STLV-III, HTLV-III or HTLV-IV is currently available. All cell lines yield a PCR product of approximately 1.8 kB. A small sideband at 657 bp is visible which could serve as an internal control for DNA integrity.

A total of 42 complete Deltaretrovirus isolates could be retrieved from the EMBL/Genbank/DDBJ nucleotide sequence database (Table 1). These included 13 HTLV-I, 12 HTLV-II, 4 STLV-I, 3 STLV-II, 5 STLV-III, and 5 BLV isolates. A common feature of the Deltaretrovirus genus is the use of proline tRNA as a primer for the complementary minus-strand DNA synthesis. tRNA genes are highly conserved between different species [15]. Alignment of the collected sequences showed a very high degree of conservation of this functionally important region. Additionally, a second highly conserved region was identified approximately 1.8 kb 3' of the tRNA binding site (Fig. 1). The *pol *ORF of HTLV-I/-II/BLV is expressed by using two ribosomal -1 frame shifts, and the second frame shift with the transcription start site of the *pol *ORF lies within this region [1]. The high degree of conservation of this region is thus understandable. A phylogenetic tree constructed from the aligned region illustrates the genetic relationships (Fig. 2). No other genomic regions with a similarly high degree of conservation were identified (see Additional File 1). Degenerate primers complementary to these regions were constructed (Fig. 1). The degeneracy of the primers was moderate (4-fold for (+) and 12-fold for (-)). The PCR was tested using serial dilutions of Deltaretrovirus-infected cell line DNA in human leukocyte DNA under various conditions (Fig. 3). Retrovirus-infected cell lines frequently harbor more than one copy of the virus, although often some of these copies are defective. When calculating PCR sensitivity this factor has to be taken into account. The HTLV-I/HTLV-II/BLV copy number has been determined in various cell lines which revealed copy numbers between 1 and 17 per cell [16-18]. We thus assumed a sensitivity of 10^-2 ^– 10^-3 ^for our PCR. This sensitivity appeared highly sufficient for our purpose. The PCR produced a faint 657 bp sideband when testing human DNA or cell line DNA diluted in human DNA (Fig. 3). Cloning and sequencing of the 657 bp product (EMBL nucleotide sequence database Acc No [EMBL:AM422011]) and a successive BLAST search revealed that it originated from amplification of a sequence on chromosome 3 (Acc No [EMBL:AC114481], Ncl 81342-80686) and 11 (Acc No [EMBL:AP000785], Ncl 74948–75403) by primer *delta-F*.

**Table 1:** Accession number of the 42 virus isolates used in the nucleotide sequence alignments.

**Virus**	**EMBL/Genbank/DDBJ accession number**
HTLV-I	[EMBL:AY563954] (Brazilian isolate), [EMBL:AY563953] (Brazilian isolate), [GenBank:NC_001436], [EMBL:AF259264] (isolate WHP from China), [EMBL:AF139170] (from an HTLV-I/II seroindeterminate patient), [EMBL:J02029] (Japanese ATL isolate), [EMBL:AF033817], [EMBL:L03561], [EMBL:D13784] (Caribbean isolate), [EMBL:L02534] (Melanesian isolate), [EMBL:U19949] (isolate from an ATL patient), [EMBL:AF042071] (isolate from Germany), [EMBL:L36905] (from a patient with post-transfusion spastic paraparesis)
HTLV-II	[GenBank:NC_001488], [EMBL:AF326584] (Brazilian isolate), [EMBL:AF326583] (Brazilian isolate, strain RP329), [EMBL:AF412314] (with HIV coinfection), [EMBL:AF139382] (Brazilian isolate), [EMBL:AF074965] (isolate from a Guahibo Indian from Venezuela), [EMBL:M10060], [EMBL:L11456] (Guyami Indian isolate), [EMBL:Y14365] (Congolese Bambute Efe Pygmy isolate), [EMBL:X89270] (Italian isolate), [EMBL:L20734], [EMBL:Y13051] (African isolate, subtype b)
STLV-I	[GenBank:NC_000858] (from a naturally infected tantalus monkey from Central Africa), [EMBL:AY590142] (in *Macaca arctoides*), [EMBL:Z46900] (from Celebes macaques), [EMBL:AF074966] (isolate Tan90 from Central African Republic)
STLV-II	[GenBank:NC_001815], [EMBL:Y14570] (STLV-PP from *Pan paniscus*), [EMBL:U90557] (from *Pan paniscus*)
STLV-III	[EMBL:AF517775] (from *Papio hamadryas papio *from Senegal), [GenBank:NC_003323] (from red-capped mangabeys (*Cercocebus torquatus*) from Cameroon), [EMBL:AY217650] (from *Theropithecus gelada*), [EMBL:AY222339] (from a red-capped mangabey – *Cercocebus torquatus torquatus *– from Nigeria), [EMBL:Y07616] (STLV-PH969 from a Hamadryas baboon)
BLV	[GenBank:NC_001414], [EMBL:AF257515] (from a Holstein cow from Argentina), [EMBL:K02120] (Japanese isolate), [EMBL:AF033818], [EMBL:D00647] (Australian isolate)

It should be noted that the PCR sensitivity could be further increased to 10^-3 ^– 10^-4 ^(while retaining specificity) by lowering the annealing temperature to 60°C and increasing the number of PCR cycles but at the expense of a stronger 657 bp sideband.

A total of 83 samples were obtained from patients with various rare mature T-cell (N = 31) and precursor T-cell neoplasms (N = 50) and from 2 patients with NK-cell disorders. All samples had been thoroughly characterized immunologically and genetically and contained a high percentage (>= 50%) of malignant cells (Table 2). All samples were fresh (i.e. unfixated) tumor material, and the DNA quality was ensured by various control PCRs.

**Table 2:** Patient and disease characteristics.

**Disease entity (N)**		**N**	**Median age (range) [years]**
Precursor T-cell (50)	Early T-cell lymphoblastic	11	
	Cortical (thymic) T-cell lymphoblastic	31	34 (17–63)
	Mature T-cell lymphoblastic	8	
Mature T-cell (31)	T-prolymphocytic	16	
	Sézary syndrome and Mycosis fungoides	5	
	Ki-1 large T-cell lymphoma	3	65 (48–83)
	Intestinal T-cell lymphoma	1	
	Other (unspecified) peripheral T-NHL	6	
NK cell disorders (2)	LGL leukemia	2	29 and 78

None of the investigated samples yielded a PCR product indicative of the presence of a Deltaretrovirus. The parallel investigation of positive controls led to the expected results.

## Discussion

While the etiological involvement of HTLV-I in endemic adult T-cell leukemia/lymphoma is beyond dispute there have been repeated controversies whether this virus might also play a role in other T-cell neoplasms such as T-prolymphocytic leukemia [19], Sézary syndrome or Mycosis fungoides [20-22]. The situation is further complicated by the fact that the classification of T-NHLs has been evolving and changing over the years as new disease entities are recognized and refined diagnostic criteria are established [23,24]. Thus the results of older studies may not always be fully transferable to today's situation. Some investigators have also suggested that a truncated HTLV-I may play a role in certain T-NHLs [19,25]. On the other hand HTLV-II has not been convincingly linked to any specific malignant T-cell disorder. The simian Deltaretroviruses are implicated in lymphomatous diseases in various simian hosts (reviewed in [1]). The newly discovered HTLV-III and HTLV-IV isolates have not yet been fully characterized, and their distribution or possible involvement in human disease is unknown.

A few previous investigations performed on precursor T-cell neoplasms in Germany were mainly based on HTLV-I serology [26] which may not be as reliable as nucleic acid-based techniques [27]. Germany has a low HTLV-I seroprevalence, but virus isolates without linkage to endemic areas have occasionally been reported [28].

The causes of most malignant T-cell disorders are only partially understood. Numerous recurrent genetic aberrations have been described [29] but a clear and detailed model of disease development still does not exist. Oncogenic viruses such as Epstein-Barr virus or HTLV-I are well established causative factors in various human T-cell lymphomas and leukemias [29]. It appears possible that yet undetected Deltaretroviruses may play a role in human T-cell malignancies.

We developed a consensus PCR for detecting Deltaretroviruses based on highly conserved genomic sequences of all published complete Deltaretrovirus isolates. Since this PCR is based on highly interspecies conserved sequence motifs it may also be capable of detecting related but hitherto unknown Deltaretroviruses. However, despite high sensitivity and specificity of our PCR approach, no Deltaretrovirus-positive cases were found in our series of samples.

## Conclusion

The results rule out a role of known Deltaretroviruses in the disease entities under investigation here. The involvement of a hitherto undetected Deltaretrovirus is not completely excluded but rendered more unlikely. Truncated proviruses that have lost their 5'-region with the tRNA binding site may also escape detection by our PCR system. Despite these negative results, our newly established consensus PCR may be a useful tool in the search for and characterization of new Deltaretroviruses in primates and other mammals.

## Methods

### Cell lines

The following cell lines were used: *BL3.1 *(infected with BLV, a bovine lymphoma cell line, [30]), *MJ [G11] *(infected with HTLV-I, derived from a human cutaneous T-cell lymphoma, [31]) and *Mo T *(infected with HTLV-II, derived from a patient with hairy cell leukemia, [32]). All cell lines were obtained from the ATCC (Acc No CRL-2306, TIB-8294, and TIB-8066, respectively). Cell culture was done according to the recommendations of the supplier. DNA isolated from the cell lines was used to prepare serial dilution rows.

### DNA isolation

DNA was isolated from sample material or cell lines using the *PureGene *kit (Biozym Diagnostik, Hessisch Oldendorf/Germany) and dissolved in Tris/EDTA buffer at a concentration of 60 ng/μl.

### Preparation of cell line dilution series

Serial dilutions of cell line DNA in DNA from buffy coats of blood donors were prepared as recently described [33].

### Patient samples

All samples were obtained for diagnostic purposes, and we retrospectively investigated archived material. The patients had given their consent for scientific investigations. The T-precursor samples were obtained within the German Multicenter Study Group for Adult Acute Lymphoblastic Leukemia (GMALL). Our study complied with the Helsinki Declaration.

### PCR method

The HotStarTaq kit (QIAGEN, Hilden/Germany) was used with 200 ng sample DNA, 400 nM of each PCR primer *delta-F *5'-CARKTGGGGGCTCGTCCGGGAT-3' and *delta-R *5'-GGCCTGGAGGCGYTCHRGTTTAA-3', buffer conditions and polymerase mix as recommended by the supplier. The primers were optimized derivatives of those previously published [34] and HPLC-purified. The following cycler program was used on a GeneAmp 2400 Cycler (PerkinElmer): 94°C for 15 minutes, 40 cycles (94°C for 20 seconds, 62°C for 20 seconds, 72°C for 90 seconds), 4°C.

### Nucleotide sequence alignments

All available complete nucleotide sequences from Deltaretrovirus isolates were collected from the EMBL/Genbank/DDBJ database (Table 1), converted into FASTA file format, and aligned using the ClustalX software [35].

### Phylogenetic analysis

The PHYLIP program package [36], version 3.65 for MacOS X, with the program modules *dnacomp *and *drawgram *was used with the default parameters to construct a phylogenetic tree from the aligned sequences.

### Immunophenotyping

Immunophenotyping by FACS analysis was done essentially by standard methods described elsewhere [37].

## Abbreviations

HTLV human T-cell lymphotropic virus

STLV simian T-cell lymphotropic virus

BLV bovine leukemia virus

PTLV primate T-cell lymphotropic virus

PCR polymerase chain reaction

PLL prolymphocytic leukemia

NHL Non Hodgkin lymphoma

ALL acute lymphoblastic leukemia

## Competing interests

The author(s) declare that they have no competing interests.

## Authors' contributions

TB designed and performed the laboratory work, particularly the PCR, did the alignments and phylogenetic analysis and wrote the paper. SS and ET performed immunophenotyping of samples. MH characterized specific samples by analysis of T-cell receptor clonality. DH is chairman of the German Multicenter Study Group for Adult Acute Lymphoblastic Leukemia (GMALL) which provided the T-ALL samples. All authors have read and approved the manuscript.

## Supplementary Material

Additional File 1 contains an alignment of the 42 retrovirus sequences from Table 1 (the genome region between primer *delta-F *and *delta-R*). Click here for file
