# Supplementary material for: No genetic evidence for involvement of Deltaretroviruses in adult patients with precursor and mature T-cell neoplasms
Source: Retrovirology. 2007 Feb 7;4:11. doi: 10.1186/1742-4690-4-11 (PMC1802090; doi:10.1186/1742-4690-4-11)

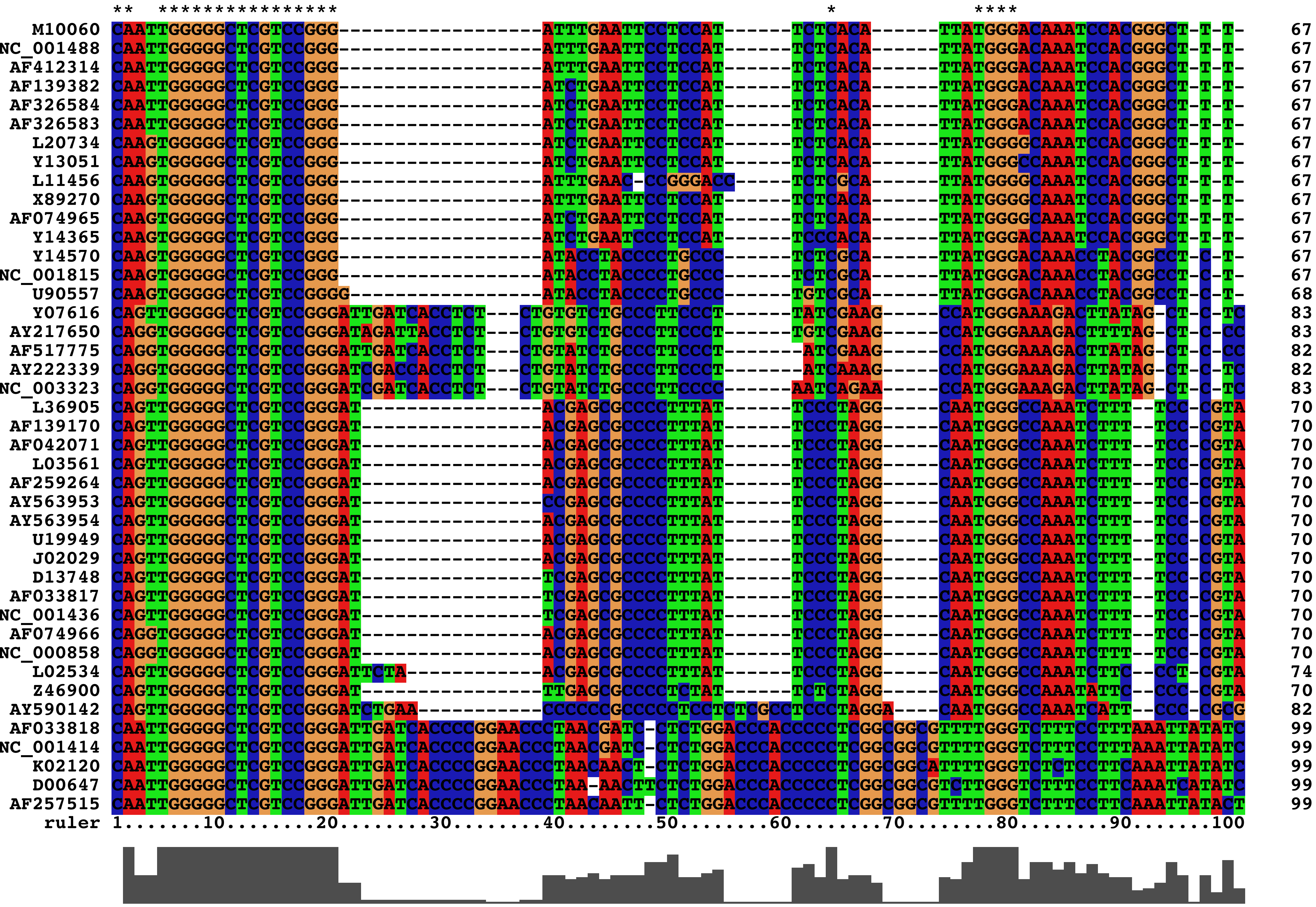

M10060 67
NC_001488 67
AF412314 67
AF139382 67
AF326584 67
AF326583 67
L20734 67
Y13051 67
L11456 67
X89270 67
AF074965 67
Y14365 67
Y14570 67
NC_001815 67
U90557 68
Y07616 83
AY217650 83
AF517775 82
AY222339 82
NC_003323 83
L36905 70
AF139170 70
AF042071 70
L03561 70
AF259264 70
AY563953 70
AY563954 70
U19949 70
J02029 70
D13748 70
AF033817 70
NC_001436 70
AF074966 70
NC_000858 70
L02534 74
Z46900 70
AY590142 82
AF033818 99
NC_001414 99
K02120 99
D00647 99
AF257515 99
ruler 1.......10........20........30........40........50........60........70........80........90.......100

```
                *   **           **  *     *     *    **   *** * **  * *  **     *  *  *  *  *  *   * ** **
   M10060 -C-CCCAACTCCAATACCCAAAGCC-CCCAGGGGGCTATCAACCCACCA--CTGGCTTAACTTTCTCCAGGCTGCTTACCGCTTGCAGCCTA-GGCCCTC   161
NC_001488 -C-CCCAACTCCAATACCCAAAGCC-CCCAGGGGGCTATCAACCCACCA--CTGGCTTAACTTTCTCCAGGCTGCTTACCGCTTGCAGCCTA-GGCCCTC   161
 AF412314 -C-CCCAACTCCAATACCCAAAGCC-CCCAGGGGGCTATCGACCCACCA--CTGGCTTAACTTTCTCCAGGCTGCTTACCGCTTGCAGCCTG-GGCCCTC   161
 AF139382 -C-CCCAACTCCAATACCCAAAGCC-CCCAGGGGGCTATCGACCCACCA--CTGGCTTAACTTTCTCCAGGCTGCTTATCGCTTGCAGCCTG-GGCCCTC   161
 AF326584 -C-CCCAACTCCAATACCCAAAGCC-CCCAGGGGGCTATCGACCCACCA--CTGGCTTAACTTTCTCCAGGCTGCTTACCGCTTGCAGCCTG-GGCCCTC   161
 AF326583 -C-CCCAACTCCAATACCCAAAGCC-CCCAGGGGGCTATCGACCCACCA--CTGGCTTAACTTTCTCCAGGCTGCTTACCGCTTGCAGCCTG-GGCCCTC   161
   L20734 -C-CCCAACTCCAATACCCAAGGCC-CCCAGGGGGCTATCGACCCACCA--CTGGCTTAATTTTCTCCAGGCTGCTTACCGCCTGCAGCCTG-GGCCCTC   161
   Y13051 -C-CCCAACTCCAATACCCAAGGCC-CCCAGGGGGCTATCGACCCACCA--CTGGCTTAATTTTCTCCAGGCTGCTTACCGCCTGCAGCCTG-GGCCCTC   161
   L11456 -C-CCCAACTCCAATACCCAAGGCC-CCCAGGGGGCTATCGACCCACCA--CTGGCTTAATTTTCTCCAGGCTGCTTACCGCCTGCAGCCTG-GGCCCTC   161
   X89270 -C-CCCAACTCCAATACCCAAGGCC-CCCAGGGGGCTATCGACCCACCA--CTGGCTTAATTTTCTCCAGGCTGCTTACCGCCTGCAGCCTG-GGCCCTC   161
 AF074965 -C-CCCAACTCCAATACCCAAGGCC-CCCAGGGGGCTATCGACCCACCA--CTGGCTTAATTTTCTCCAGGCTGCTTACCGCCTGCAGCCTG-GGCCCTC   161
   Y14365 -C-CCCAACTCCAATACCCAAGGCC-CCCAGGGGGCTATCGACCCACCA--CTGGCTTAACTTTCTCCAGGCTGCTTACCGCTTGCAGCCTG-GGCCCTC   161
   Y14570 -CGTCCAGC-CCAATCCCCAAGGCC-CCCAGGGGGTTATCAACCCACCA--TTGGTTAAACTTCCTCCAAGCCGCCTACCGGCTACAACCTG-GGCCCTC   161
NC_001815 -CGTCCAGC-CCAATCCCCAAGGCC-CCCAGGGGGTTATCAACCCACCA--TTGGTTAAACTTCCTCCAAGCCGCCTACCGGCTACAACCTG-GGCCCTC   161
   U90557 -CGCCTAGC-CCAATCCCCAAGGCC-CCCAGAGGTTTATCAACCCACCA--CTGGTTAAATTTCCTCCAAGCCTCCTACCGGCTACAACCTG-GGCCCTC   162
   Y07616 -CGGCAAAC-CCTATCCCCAAGGCC-CCAAAAGGGCTAGCAATCCACCA--TTGGTTAAACTTCCTACAAGCCGCCTATCGGTTACAGCCAG-GGCCTTC   177
 AY217650 -CGGCAAAC-CCTATCCCCAAGGCC-CCAAAAGGGCTAGCAATCCACCA--TTGGTTAAACTTCCTACAAGCCGCCTATCGGTTACAGCCAG-GGCCTTC   177
 AF517775 -CGACAAAT-CCTATCCCCAAAGCC-CCAAAGGGGCTAGCAATTCACCA--CTGGCTAAACTTCCTCCAAGCCGCGTACCGACTACAGCCAG-GGCCTTC   176
 AY222339 -CAACAAAC-CCTATCCCCAAAGCC-CCAAAGGGGCTAGCAATTCACCA--CTGGCTAAACTTCCTCCAAGCCGCGTACCGACTACAGCCAG-GGCCTTC   176
NC_003323 -CGGTAAAC-CCTATCCCCAAAGCC-CCAAAAGGGCTAGCAATTCACCA--CTGGCTAAACTTTCTCCAAGCCGCGTACCGACTGCAGCCAG-GGCCCTC   177
   L36905 GC-GCTAGC-CCTAT-TCCGCGGCCGCCCCAGGGGCTGGCCGCTCATCA--CTGGCTTAACTTCCTCCAAGCGGCATATCGCCTAGAACCCG-GTCCCTC   164
 AF139170 GC-GCTAGC-CCTAT-TCCGCGGCCGCCCCGGGGGCTGGCCGCTCATCA--CTGGCTTAACTTCCTCCAAGCGGCATATCGCCTAGAACCCG-GTCCCTC   164
 AF042071 GC-GCTAGC-CCTAT-TCCGCGGCCGCCCCGGGGGCTGGCCGCTCATCA--CTGGCTTAACTTCCTCCAAGCGGCATATCGCCTAGAACCCG-GTCCCTC   164
   L03561 GC-GCTAGC-CCTAT-TCCGCGGCCGCCCCGGGGGCTGGCCGCTCATCA--CTGGCTTAACTTCCTCCAAGCGGCATATCGCCTAGAACCCG-GTCCCTC   164
 AF259264 GC-GCTAGC-CCTAT-TCCGCGGCCGCCCCGGGGGCTGGCCGCTCATCA--CTGGCTTAACTTCCTCCAAGCGGCATATCGCCTAGAACCCG-GTCCCTC   164
 AY563953 GC-GCTAGC-CCTAT-TCCGCGGCCGCCCCGGGGGCTGGCCGCTCATCA--CTGGCTTAACTTCCTCCAGGCGGCATATCGCCTAGAACCCG-GTCCCTC   164
 AY563954 GC-GCTAGC-CCTAT-TCCGCGGCCGCCCCGGGGGCTGGCCGCTCATCA--CTGGCTTAACTTCCTCCAAGCGGCATATCGCCTAGAACCCG-GTCCCTC   164
   U19949 GC-GCTAGC-CCTAT-TCCGCGGCCGCCCCGGGGGCTGGCCGCTCATCA--CTGGCTTAACTTCCTCCAGGCGGCATATCGCCTAGAACCCG-GTCCCTC   164
   J02029 GC-GCTAGC-CCTAT-TCCGCGACCGCCCCGGGGGCTGGCCGCTCATCA--CTGGCTTAACTTCCTCCAGGCGGCATATCGCCTAGAACCCG-GTCCCTC   164
   D13748 GC-GCTAGC-CCTAT-TCCGCGGCCGCCCCGGGGGCTGGCCGCTCATCA--CTGGCTTAACTTCCTCCAGGCGGCATATCGCCTAGAACCCG-GTCCCTC   164
 AF033817 GC-GCTAGC-CCTAT-TCCGCGGCCGCCCCGGGGGCTGGCCGCTCATCA--CTGGCTTAACTTCCTCCAGGCGGCATATCGCCTAGAACCCG-GTCCCTC   164
NC_001436 GC-GCTAGC-CCTAT-TCCGCGGCCGCCCCGGGGGCTGGCCGCTCATCA--CTGGCTTAACTTCCTCCAGGCGGCATATCGCCTAGAACCCG-GTCCCTC   164
 AF074966 GC-GCTAGC-CCTAT-TCCGCGACCGCCCCGGGGGCTGGCTGCTCATCA--CTGGCTTAACTTCCTCCAGGCGGCATATCGCCTAGAACCCG-GTCCCTC   164
NC_000858 GC-GCTAGC-CCTAT-TCCGCGACCGCCCCGGGGGCTGGCTGCTCATCA--CTGGCTTAACTTCCTCCAGGCGGCATATCGCCTAGAACCCG-GTCCCTC   164
   L02534 GC-GCTAAC-CCTAT-CCCGCGGCCGCCCCGGGGGCTGGCCACTCATCA--CTGGCTTAATTTCCTCCAGGCGGCATACCGCCTGGAGCCCG-GTCCTTC   168
   Z46900 TC-ACTAGC-CCCAT-TCCGCGGCCGCCCCGGGGGCTGGCCGCTCACCA--CTGGCTTAATTTTCTTCAGGCAGCATATCGCTTAGAGCCCG-GTCCTTC   164
 AY590142 TC-ACCAGC-CCTAT-TCCCCGGCCCCCCCGGGGGCTAGCTACACACCA--CTGGCTCAATTTTCTCCAGGCGGCCTATCGCTTAGAACCTG-GTCCCTC   176
 AF033818 ATGGGAAATTCCCCCTCCTATAACCCCCCCGCTGG-TATCTCCCCCTCAGACTGGCTCAACCTTCGTCAAAGCGCGCAAAGGCT-CAATCCGCGACCCTC   197
NC_001414 ATGGGAAATTCCCCCTCCTATAACCCCCCCGCTGG-TATCTCCCCCTCAGACTGGCTCAACCTTCGTCAAAGCGCGCAAAGGCT-CAATCCGCGACCCTC   197
   K02120 ATGGGAAATTCCCCCTCCTATAACCCCCCCGCTGG-TATCTCCCCCTCAGACTGGCTCAACCTTCTGCAAAGCGCGCAAAGGCT-CAATCCGCGACCCTC   197
   D00647 ATGGGAAACTCCCCCTCCTATAACCCCCCCGCTGG-CATCTCCCCCTCAGACTGGCTCAACCTCCTGCAAAGCGCGCAAAGGCT-CAATCCACGACCCTC   197
 AF257515 ATGGGAAATTCCCCTTCTTATAACCCTCCCGCTGG-TATCTCCCCCTCAGACTGGCTCAACCTTCTGCAAAGCGCGCAAAGGCT-CAATCCGCGACCCTC   197
    ruler .......110.......120.......130.......140.......150.......160.......170.......180.......190.......200
```

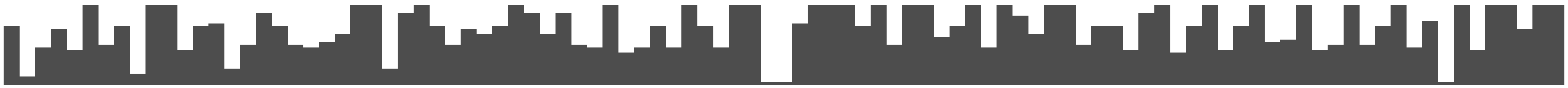

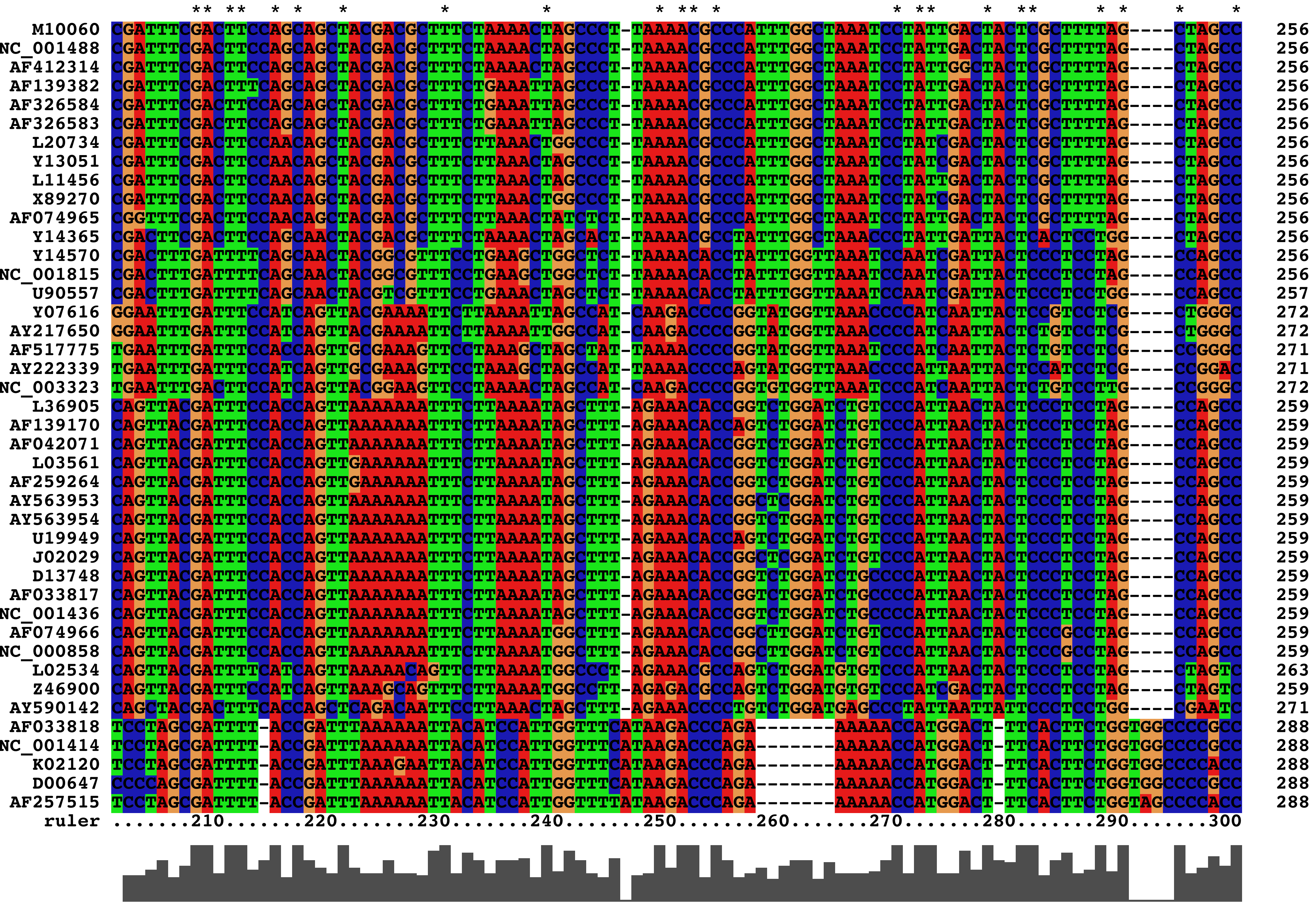
M10060 CGATTTCGACTTCCAGCAGCTACGACGCTTTCTAAAACTAGCCCT-TAAAACGCCCATTTGGCTAAATCCTATTGACTACTCGCTTTTAG----CTAGCC 256
NC_001488 CGATTTCGACTTCCAGCAGCTACGACGCTTTCTAAAACTAGCCCT-TAAAACGCCCATTTGGCTAAATCCTATTGACTACTCGCTTTTAG----CTAGCC 256
AF412314 CGATTTCGACTTCCAGCAGCTACGACGCTTTCTAAAACTAGCCCT-TAAAACGCCCATTTGGCTAAATCCTATTGGCTACTCGCTTTTAG----CTAGCC 256
AF139382 CGATTTCGACTTTCAGCAGCTACGACGCTTTCTGAAATTAGCCCT-TAAAACGCCCATTTGGCTAAATCCTATTGACTACTCGCTTTTAG----CTAGCC 256
AF326584 CGATTTCGACTTCCAGCAGCTACGACGCTTTCTGAAATTAGCCCT-TAAAACGCCCATTTGGCTAAATCCTATTGACTACTCGCTTTTAG----CTAGCC 256
AF326583 CGATTTCGACTTCCAGCAGCTACGACGCTTTCTGAAATTAGCCCT-TAAAACGCCCATTTGGCTAAATCCTATTGACTACTCGCTTTTAG----CTAGCC 256
L20734 CGATTTCGACTTCCAACAGCTACGACGCTTTCTTAAACTGGCCCT-TAAAACGCCCATTTGGCTAAATCCTATCGACTACTCGCTTTTAG----CTAGCC 256
Y13051 CGATTTCGACTTCCAACAGCTACGACGCTTTCTTAAACTAGCCCT-TAAAACGCCCATTTGGCTAAATCCTATCGACTACTCGCTTTTAG----CTAGCC 256
L11456 CGATTTCGACTTCCAACAGCTACGACGCTTTCTTAAACTAGCCCT-TAAAACGCCCATTTGGCTAAATCCTATTGACTACTCGCTTTTAG----CTAGCC 256
X89270 CGATTTCGACTTCCAACAGCTACGACGCTTTCTTAAACTGGCCCT-TAAAACGCCCATTTGGCTAAATCCTATCGACTACTCGCTTTTAG----CTAGCC 256
AF074965 CGGTTTCGACTTCCAACAGCTACGACGCTTTCTTAAACTATCTCT-TAAAACGCCCATTTGGCTAAATCCTATTGACTACTCGCTTTTAG----CTAGCC 256
Y14365 CGACTTCGACTTCCAGCAACTACGACGCTTTCTAAAACTAGCACT-TAAAACGCCTATTTGGCTAAACCCTATTGATTACTCACTCCTGG----CTAGCC 256
Y14570 CGACTTTGATTTTCAGCAACTACGGCGTTTCCTGAAGCTGGCTCT-TAAAACACCTATTTGGTTAAATCCAATCGATTACTCCCTCCTAG----CCAGCC 256
NC_001815 CGACTTTGATTTTCAGCAACTACGGCGTTTCCTGAAGCTGGCTCT-TAAAACACCTATTTGGTTAAATCCAATCGATTACTCCCTCCTAG----CCAGCC 256
U90557 CGACTTTGATTTTCAGCAACTACGTCGTTTCCTGAAACTAGCTCT-TAAAACACCTATTTGGTTAAATCCAATCGATTACTCCCTCCTGG----CCAGCC 257
Y07616 GGAATTTGATTTCCATCAGTTACGAAAATTCTTAAAATTAGCCAT-CAAGACCCCGGTATGGTTAAACCCCATCAATTACTCCGTCCTCG----CTGGGC 272
AY217650 GGAATTTGATTTCCATCAGTTACGAAAATTCTTAAAATTGGCCAT-CAAGACCCCGGTATGGTTAAACCCCATCAATTACTCTGTCCTCG----CTGGGC 272
AF517775 TGAATTTGATTTCCACCAGTTGCGAAAGTTCCTAAAGCTAGCTAT-TAAAACCCCGGTATGGTTAAATCCCATCAATTACTCTGTCCTCG----CCGGGC 271
AY222339 TGAATTTGATTTCCATCAGTTGCGAAAGTTCCTAAAGCTAGCCAT-TAAAACCCCAGTATGGTTAAACCCCATTAATTACTCCATCCTCG----CCGGAC 271
NC_003323 TGAATTTGACTTCCATCAGTTACGGAAGTTCCTAAAACTAGCCAT-CAAGACCCCGGTGTGGTTAAATCCCATCAATTACTCTGTCCTTG----CCGGGC 272
L36905 CAGTTACGATTTCCACCAGTTAAAAAAATTTCTTAAAATAGCTTT-AGAAACACCGGTCTGGATCTGTCCCATTAACTACTCCCTCCTAG----CCAGCC 259
AF139170 CAGTTACGATTTCCACCAGTTAAAAAAATTTCTTAAAATAGCTTT-AGAAACACCAGTCTGGATCTGTCCCATTAACTACTCCCTCCTAG----CCAGCC 259
AF042071 CAGTTACGATTTCCACCAGTTAAAAAAATTTCTTAAAATAGCTTT-AGAAACACCGGTCTGGATCTGTCCCATTAACTACTCCCTCCTAG----CCAGCC 259
L03561 CAGTTACGATTTCCACCAGTTGAAAAAATTTCTTAAAATAGCTTT-AGAAACACCGGTCTGGATCTGTCCCATTAACTACTCCCTCCTAG----CCAGCC 259
AF259264 CAGTTACGATTTCCACCAGTTGAAAAAATTTCTTAAAATAGCTTT-AGAAACACCGGTCTGGATCTGTCCCATTAACTACTCCCTCCTAG----CCAGCC 259
AY563953 CAGTTACGATTTCCACCAGTTAAAAAAATTTCTTAAAATAGCTTT-AGAAACACCGGCTCGGATCTGTCCCATTAACTACTCCCTCCTAG----CCAGCC 259
AY563954 CAGTTACGATTTCCACCAGTTAAAAAAATTTCTTAAAATAGCTTT-AGAAACACCGGTCTGGATCTGTCCCATTAACTACTCCCTCCTAG----CCAGCC 259
U19949 CAGTTACGATTTCCACCAGTTAAAAAAATTTCTTAAAATAGCTTT-AGAAACACCAGTCTGGATCTGTCCCATTAACTACTCCCTCCTAG----CCAGCC 259
J02029 CAGTTACGATTTCCACCAGTTAAAAAAATTTCTTAAAATAGCTTT-AGAAACACCGGCTCGGATCTGTCCCATTAACTACTCCCTCCTAG----CCAGCC 259
D13748 CAGTTACGATTTCCACCAGTTAAAAAAATTTCTTAAAATAGCTTT-AGAAACACCGGTCTGGATCTGCCCCATTAACTACTCCCTCCTAG----CCAGCC 259
AF033817 CAGTTACGATTTCCACCAGTTAAAAAAATTTCTTAAAATAGCTTT-AGAAACACCGGTCTGGATCTGCCCCATTAACTACTCCCTCCTAG----CCAGCC 259
NC_001436 CAGTTACGATTTCCACCAGTTAAAAAAATTTCTTAAAATAGCTTT-AGAAACACCGGTCTGGATCTGCCCCATTAACTACTCCCTCCTAG----CCAGCC 259
AF074966 CAGTTACGATTTCCACCAGTTAAAAAAATTTCTTAAAATGGCTTT-AGAAACACCGGCTTGGATCTGTCCCATTAACTACTCCCGCCTAG----CCAGCC 259
NC_000858 CAGTTACGATTTCCACCAGTTAAAAAAATTTCTTAAAATGGCTTT-AGAAACACCGGCTTGGATCTGTCCCATTAACTACTCCCGCCTAG----CCAGCC 259
L02534 CAGTTACGATTTTCATCAGTTAAAAACAGTTCTTAAAATGGCCCT-AGAAACGCCAGTCTGGATGTGTCCCATTAACTACTCCCTCCTAG----CTAGTC 263
Z46900 CAGTTACGATTTCCATCAGTTAAAGCAGTTTCTTAAAATGGCCTT-AGAGACGCCAGTCTGGATGTGTCCCATCGACTACTCCCTCCTAG----CTAGTC 259
AY590142 CAGCTACGACTTTCACCAGCTCAGACAATTCCTTAAACTAGCTTT-AGAAACCCCTGTCTGGATGAGCCCTATTAATTATTCCCTCCTGG----CGAATC 271
AF033818 TCCTAGCGATTTT-ACCGATTTAAAAAATTACATCCATTGGTTTCATAAGACCCAGA-------AAAAACCATGGACT-TTCACTTCTGGTGGCCCCGCC 288
NC_001414 TCCTAGCGATTTT-ACCGATTTAAAAAATTACATCCATTGGTTTCATAAGACCCAGA-------AAAAACCATGGACT-TTCACTTCTGGTGGCCCCGCC 288
K02120 TCCTAGCGATTTT-ACCGATTTAAAGAATTACATCCATTGGTTTCATAAGACCCAGA-------AAAAACCATGGACT-TTCACTTCTGGTGGCCCCACC 288
D00647 CCCCAGCGATTTT-ACCGATTTAAAAAATTACATCCATTGGTTTCATAAGACCCAGA-------AAAAACCATGGACT-TTCACTTCTGGTGGCCCCGCC 288
AF257515 TCCTAGCGATTTT-ACCGATTTAAAAAATTACATCCATTGGTTTTATAAGACCCAGA-------AAAAACCATGGACT-TTCACTTCTGGTAGCCCCACC 288
ruler .......210.......220.......230.......240.......250.......260.......270.......280.......290.......300

```
          *  *  * *        *  * **              *  *        *           *          *               * *
M10060    TTATCCCCAA--GGGATATCCAGGAAGGGTGGTAGAGATTATAAATATCCTTGTCAAAAATCAAG--TCTCCCCTAGCGCCCCCGCCGCCCCAGTT-CCG   351
NC_001488 TTATCCCCAA--GGGATATCCAGGAAGGGTGGTAGAGATTATAAATATCCTTGTCAAAAATCAAG--TCTCCCCTAGCGCCCCCGCCGCCCCAGTT-CCG   351
AF412314  TTATCCCCAA--GGGATATCCAGGAAGGGTGGTAGAGATTATAAATATCCTTGTCAAAAATCAAG--TCTCCCCTAGCGCCCCCGCCGCCCCAGTT-CCG   351
AF139382  TTATCCCCAA--GGGATATCCAGGAAGGGTGGTAGAGATTATAAATATCCTTGTCAAAAATCAAG--TCTCCCCTAGCGCCCCCGCCGCCCCAGTT-CCG   351
AF326584  TTATCCCCAA--GGGATATCCAGGAAGGGTGGTAGAGATTATAAATATCCTTGTCAAAAATCAAG--TCTCCCCTAGCGCCCCCGCCGCCCCAGTT-CCG   351
AF326583  TTATCCCCAA--GGGATATCCAGGAAGGGTGGTAGAGATTATAAATATCCTTGTCAAAAATCAAG--TCTCCCCTAGCGCCCCCGCCGCCCCAGTT-CCG   351
L20734    TTATCCCCAA--AGGATATCCGGGAAGGGTGGTAGAGATTATAAACATCCTTGTCAAAAACCAAG--TCTCCCCTAGCGCCCCCGCCGCCCCAGTT-CCG   351
Y13051    TTATCCCCAA--AGGATACCCGGGAAGGGTGGTAGAGATTATAAACATCCTTGTCAAAAACCAAG--TCTCCCCTAGCGCCCCCGCCGCCCCAGTT-CCG   351
L11456    TTATCCCCAA--AGGATATCCGGGAAGGGTGGTAGAGATTATAAACATCCTTGTCAAAAACCAAG--TCTCCCCTAGCGCCCCCGCCGCCCCAGTT-CCG   351
X89270    TTGTCCCCAA--AGGATATCCGGGAAGGGTGGTAGAGATTATAAACATCCTTGTCAAAAATCAAG--TCTCCCCTAGCGCCCCCGCCGCCCCAGTT-CCG   351
AF074965  TTATCCCCAA--AGGATATCCGGGAAGGGTGGTAGAGATTATATACATCCTTGTCAAAAACCAAG--TCTCCCCTAGCGCCTCACCCGCCCCAGTT-CCG   351
Y14365    TTATCCCCAA--GGGATATCCAGGAAGGGTGGTAGAAATTATAAACATCCTTGTCAAAAATCATG--TCTCCCCTAGCGCCCCCGCCGCCCCAGTT-CCG   351
Y14570    TCATCCCCAA--GGGTTACCCCGGGCGGACAATCGAAATCATTAATGTGTTAATTAAAAATCAAA--CGTCCCCCACCCCGCCTCCTGCCCC-GTC-TCT   350
NC_001815 TCATCCCCAA--GGGTTACCCCGGGCGGACAATCGAAATCATTAATGTGTTAATTAAAAATCAAA--CGTCCCCCACCCCGCCTCCTGCCCC-GTC-TCT   350
U90557    TCATCCCCAA--GGGTTACCCCGGGCGGACAAGCGAGATTATTAATGTGTTAATTAGAAATCAAG--CGTCCCCCACCCCGCCTCCTGCCCC-GTC-TCT   351
Y07616    TCATTCCCAA--AAACTACCCGGGTCGAGTCCATGAAATAGTGGCCATCCTAAT------CCAAG--AAACTCCCG-CACGCGAGACACCCCCGT---CC   358
AY217650  TCATTCCCAA--AAACTACCCGGGTCGGGTCCATGAAATAGTGGCCATCCTAAT------CCAAG--AAACTCCCG-CACGCGAGACACCCCCGT---CC   358
AF517775  TTATTCCCAA--AAACTACCCGGGCAGAGTTCATGAAATTGTAGCCATCTTAAT------TCAAG--AAACCCCTG-CGCGGGAGGCACCCCCGT---CA   357
AY222339  TAATTCCCAA--AAACTACCCGGGCAGAGTTCATGAAATAGTAGCCATCCTGAT------TCAAG--AGACCCCTA-TGCGGGAAGCGCCCCCGT---CA   357
NC_003323 TCATTCCCAA--GAACTACCCGGGCAGGGTCCATGAAATAGTAGCCATCCTAAT------TCAAG--AGACCCCTG-CGCGCGAGGCGCCCCCAT---CG   358
L36905    TACTCCCAAA--AGGATACCCCGGCCGGGTGAATGAAATTTTACACATACTCATCCAAACCCAAGCCCAGATCCCGTCCCGTCCCGCGCCACCGCCGCCG   357
AF139170  TACTCCCAAA--AGGATACCCCGGCCGGGTGAATGAAATTTTACACATACTCATCCAAACCCAAGCCCAGATCCCGTCCCGTCCCGCGCCACCGCCGCCG   357
AF042071  TACTCCCAAA--AGGATACCCCGGCCGGGTGAATGAAATTTTACACATACTCATCCAAACCCAAGCCCAGATCCCGTCCCGTCCCGCGCCACCGCCGCCG   357
L03561    TACTCCCAAA--AGGATACCCCGGCCGGGTGAATGAAATTTTACACATACTCATCCAAACCCAAGCCCAGATCCCGTCCCGTCCCGCGCCACCGCCGCCG   357
AF259264  TACTCCCAAA--AGGATACCCCGGCCGGGTGAATGAAATTTTACACATACTCATCCAAACCCAAGCCCAGATCCCGTCCCGTCCCGCGCCACCGCCGCCG   357
AY563953  TACTCCCAAA--AGGATACCCCGGCCGGGTGAATGAAATTTTACACATACTCATCCAAACCCAAGCCCAGATCCCGTCCCGTCCCGCGCCACCGCCGCCG   357
AY563954  TACTCCCAAA--AGGATACCCCGGCCGGGTGAATGAAATTTTACACATACTCATCCAAACCCAAGCCCAGATCCCGTCCCGTCCCGCGCCACCGCCGCCG   357
U19949    TACTCCCAAA--AGGATACCCCGGCCGGGTGAATGAAATTTTACACATACTCATCCAAACCCAAGCCCAGATCCCGTCCCGTCCCGCGCCACCGCCGCCG   357
J02029    TACTCCCAAA--AGGATACCCCGGCCGGGTGAATGAAATTTTACACATACTCATCCAAACCCAAGCCCAGATCCCGTCCCGTCCCGCGCCACCGCCGCCG   357
D13748    TACTCCCAAA--AGGATACCCCGGCCGGGTGAATGAAATTTTACACATACTCATCCAAACCCAAGCCCAGATCCCGTCCCGCCCCGCGCCGCCGCCGCCG   357
AF033817  TACTCCCAAA--AGGATACCCCGGCCGGGTGAATGAAATTTTACACATACTCATCCAAACCCAAGCCCAGATCCCGTCCCGCCCCGCGCCGCCGCCGCCG   357
NC_001436 TACTCCCAAA--AGGATACCCCGGCCGGGTGAATGAAATTTTACACATACTCATCCAAACCCAAGCCCAGATCCCGTCCCGCCCCGCGCCGCCGCCGCCG   357
AF074966  TACTCCCAAA--AGGGTACCCCGGCCGGGTAAATGAAATTTTACACATACTCATCCAAACCCAAGCCCAGATCCCGTGCCACCCCGCGCCACCGCCGCCG   357
NC_000858 TACTCCCAAA--AGGGTACCCCGGCCGGGTAAATGAAATTTTACACATACTCATCCAAACCCAAGCCCAGATCCCGTGCCACCCCGCGCCACCGCCGCCG   357
L02534    TACTCCCAAA--AGGATACCCTGGCCAGGTAAATGAAATTTTACAGGTACTCATCCAAACCCAAACCCAGATCCCGTCCCACCCCGCGCCGCCGCCGCCG   361
Z46900    TACTCCCGAA--AGG-TACCCCGGCCGGGTAAATGAAATTTTACACATACTTATTCAAACCCAGGCCCAGAGCCCGTCCCGCCCCGCGCCGCCGCCGCCG   356
AY590142  TCCTCCCCAA--GGGATATCCCGGCCGAGTCAATGAAATACTAAATATACTCATCCAAGCCCAGGTCCAAACCCCTCCCCATCCCCCCTCCCCGTTATCA   369
AF033818  TCATGCCCACCCGGGAAATTC-GGTCGGGTTCCCCTCGTCTTGGCCACCCTAAA------CGAAG--TACTCTCAAACGATGAGGGCGCCCCAGGT----   375
NC_001414 TCATGCCCACCCGGGAAATTC-GGTCGGGTTCCCCTCGTCTTGGCCACCCTAAA------CGAAG--TACTCTCAAACGATGAGGGCGCCCCAGGT----   375
K02120    TCATGTCCACCCGGGAGATTC-GGCCGGGTTCCCCTCGTCTTGGCCACCCTAAA------CGAAG--TACTCTCAAACGAAGGGGGCGCCCCGGGT----   375
D00647    TCATGCCCACCCGGGAAATTC-GGCCGAGTTCCCCTTGTCTTGGCCACCCTAAA------CGAAG--TGCTCTCAAACGATGAGGGCGCCCCGGGT----   375
AF257515  TCATGTCCACCCGGGAGATTC-GGCCGGGTTCCCCTTGTCTTGGCCACCCTAAA------CGAAG--TGCTCTCAAACGATGGAGGCACCCCGGGT----   375
ruler     .......310.......320.......330.......340.......350.......360.......370.......380.......390.......400
```

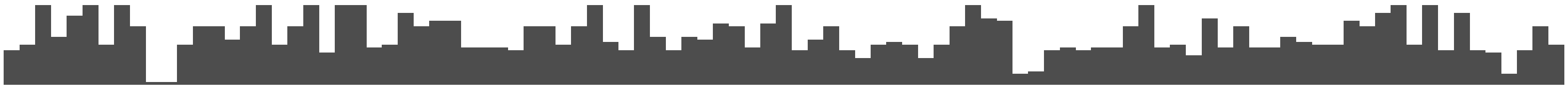

```
                 *  *   *        *
M10060     ACACCTATCTG-----CCC-TACT-ACTACTCCTCCGCCACC-----TCCCCCCCCTTCCCCGGAGGCCCATGTTCCCCCCCCTTACGTGGAACCCACCA   439
NC_001488  ACACCTATCTG-----CCC-TACT-ACTACTCCTCCGCCACC-----TCCCCCCCCTTCCCCGGAGGCCCATGTTCCCCCCCCTTACGTGGAACCCACCA   439
AF412314   ACACCTATCTG-----CCC-TACT-ACTACTCCTCCGCCACC-----TCCCCCCCCTTCCCCGGAGGCCCATGTTCCCCCCCCTTACGTGGAACCCACCA   439
AF139382   ACACCTATCTG-----CCC-TACT-ACTACTCCTCCGCCACC-----TCCCCCCCCTTCCCCGGAGGCCCATGTTCCCCCCCCTTACGTGGAACCCACCA   439
AF326584   ACACCTATCTG-----CCC-TACT-ACTACTCCTCCGCCACC-----TCCCCCCCCTTCCCCGGAGGCCCATGTTCCCCCTCCTTACGCGGAACCCACCA   439
AF326583   ACACCTATCTG-----CCC-TACT-ACTACTCCTCCGCCACC-----TGCCCCGCCGCCCCAGTATGCACATGCTTCCCCGCCCTACGGCGAACCCACCA   439
L20734     ACACCTATCTG-----CCC-TACC-ACTACCCCTCCGCCACC-----TCCCCCCCCTTCCCCGGAGGCCCATGTTCCCCCCCCTTACGTGGAACCCACTA   439
Y13051     ACACCTATCTG-----CCC-TACC-ACTACCCCTCCGCCACC-----TCCCCCCCCTTCCCCGGAGGCCCATGTCCCCCCCCCTTACGTGGAACCCACTA   439
L11456     ACACCTATCTG-----CCC-TACC-ACTACCCCTCCGCCACC-----TCCCCCCCCTTCCCCGGAGGCCCATGTCCCCCCCCCATACGTAGAACCCACTA   439
X89270     ACACCTATCTG-----CCC-CACT-ACCACTCCTCCGCCACC-----CCCCCCCCCTTCCCCGGAGGCCCATGTTCCCCCCCCTTACGTGGAACCCACCT   439
AF074965   ACTCCTATCTG-----CCC-TACC-A----------------------------------------------------------------------GGTA   374
Y14365     ACACCTATCTG-----CCC-GACG-ACTGCTCCTCCGCCACC-----GCCCCCCCCTTCCCCGGAGGGCCATGTCCCCCCCCCTTATGCAGAGCCCACTA   439
Y14570     AC-CCGAACCA-----GCTA-ACCCACCGCCCCTCCAACA-------GCCCTCGGCTCCTCCGGAACCCCATACACCCCCCCCCTATATAAAGCCTCCCG   436
NC_001815  AC-CCGAACCA-----GCTA-ACCCACCGCCCCTCCAACA-------GCCCTCGGCTCCTCCGGAACCCCATACACCCCCCCCCTATATAAAGCCTCCCG   436
U90557     AC-CCGAACCG-----GCTA-ACCCACCGCCCCTCCAGCA-------GCCCTCGGCTCCTCCGGAACCCCATACGCCCCCCCCCTATATAGAGCCTCCCG   437
Y07616     GC-CCCG-CTA-----GCAG-ATG-ACC-C------------------------------CCAAAAGCC--TCCGCCCTATCCTGA-ACAAGTGCAGACG   415
AY217650   GC-CCCG-CTA-----GCAG-ATG-ACC-C------------------------------TCAAAAGCC--TCCACCCTATCCTGA-ACAAGTGCAAACG   415
AF517775   GC-CCCA-CCA-----ACAG-ATG-ACC-C------------------------------TCAAAAGCC--TCCGCCCTACCCCGA-GCAGGCACAGGGG   414
AY222339   GC-CCCG-CCA-----GCAG-ATG-ACC-C------------------------------TCAAAAGCC--TCCGCCCTATCCCGA-GCAGGCACCAGGG   414
NC_003323  GC-CCCA-CCA-----GCAG-ATG-ACC-C------------------------------TCAAAAGCC--TCCACCCTACCCCGA-GCATGCACAGGTA   415
L36905     TCATCCC-CCA------CCC-ACG-ACCCCC------------------------CGGATTCGGATCCACAAATCCCCCCTCCCTATGTTGAACCTACGG   424
AF139170   TCATCCC-CCA------CCC-ACG-ACCCCC------------------------CGGATTCTGATCCACAAATCCCCCCTCCCTATGTTGAGCCTACGG   424
AF042071   TCATCCC-CCA------CCC-ACG-ACCCCC------------------------CGGATTCTGATCCACAAATCCCCCCTCCCTATGTTGAGCCTACGG   424
L03561     TCATCCC-CCA------CCC-ACG-ACCCCC------------------------CGGATTCTGATCCACAAATCCCCCCTCCCTATGTTGAGCCTACGG   424
AF259264   TCATCCC-CCA------CCC-ACG-ACCCCC------------------------CGGATTCTGATCCACAAATCCCCCCTCCCTATGTTGAGCCTACGG   424
AY563953   TCATCCC-CCA------CCC-ACG-ACCCCC------------------------CGGATTCTGATCCACAAATCCCCCCTCCCTATGTTGAGCCTACGG   424
AY563954   TCATCCC-CCA------CCC-ACG-ACCCCC------------------------CGGATTCTGATCCACAAATCCCCCCTCCCTATGTTGAGCCTACGG   424
U19949     TCATCCC-CCA------CCC-ACG-ACCCCC------------------------CAGATTCTGATCCACAAATCCCCCCTCCCTATGTTGAGCCTACGG   424
J02029     TCATCCC-CCA------CCC-ACG-ACCCCC------------------------CGGATTCTGATCCACAAATCCCCCCTCCCTATGTTGAGCCTACGG   424
D13748     TCATCCT-CCA------CCC-ACG-ACCCCC------------------------CGGATTCTGACCCACAAATCCCCCCTCCCTATGTTGAGCCTACAG   424
AF033817   TCATCCT-CCA------CCC-ACG-ACCCCC------------------------CGGATTCTGACCCACAAATCCCCCCTCCCTATGTTGAGCCTACAG   424
NC_001436  TCATCCT-CCA------CCC-ACG-ACCCCC------------------------CGGATTCTGACCCACAAATCCCCCCTCCCTATGTTGAGCCTACAG   424
AF074966   TCATCCC-CCA------CTC-TCG-ACCCCC------------------------CGGATTCTGATCCACAAATCCCCCCTCCTTATGTTGAGCCTACGG   424
NC_000858  TCATCCC-CCA------CTC-TCG-ACCCCC------------------------CGGATTCTGATCCACAAATCCCCCCTCCTTATGTTGAGCCTACGG   424
L02534     TCATCCC-CCA------CCC-ACG-ACCCCC------------------------CAGATTCTGATCCACAGATCCCCCCTCCCTATGTTGAGCCTACAG   428
Z46900     TCACCTC-CCA------CCC-ACA-GCCCCC------------------------CAGATTCCGATCCGCAAATCCCCCCTCCCTACATTGAGCCTACGG   423
AY590142   GCCTCTC-CCAACACTGCCC-CCA-GCTCCCAGTTTCCCGCTGAAACTAGTCTAACTGCCTCTGACCTCCACATCCCACCCCCCTATGCGGAGCCCTCTG   466
AF033818   GCATCAGCC--------CCAGAAGAACA----------------------------------------------ACCCCCCCCTTATG-----------A   410
NC_001414  GCATCAGCC--------CCAGAAGAACA----------------------------------------------ACCCCCCCCTTATG-----------A   410
K02120     GCATCGGCC--------CCAGAAGAACA----------------------------------------------ACCCCCCCCTTATG-----------A   410
D00647     GCATCGGCC--------CCAGAAGAACA----------------------------------------------ACCCCCCCCTTATG-----------A   410
AF257515   GCATCGGCC--------CCAGAAGAACA----------------------------------------------ACCCCCCCCTTATG-----------A   410
ruler      .......410.......420.......430.......440.......450.......460.......470.......480.......490.......500
```

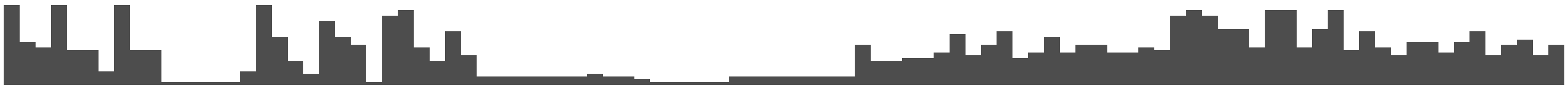

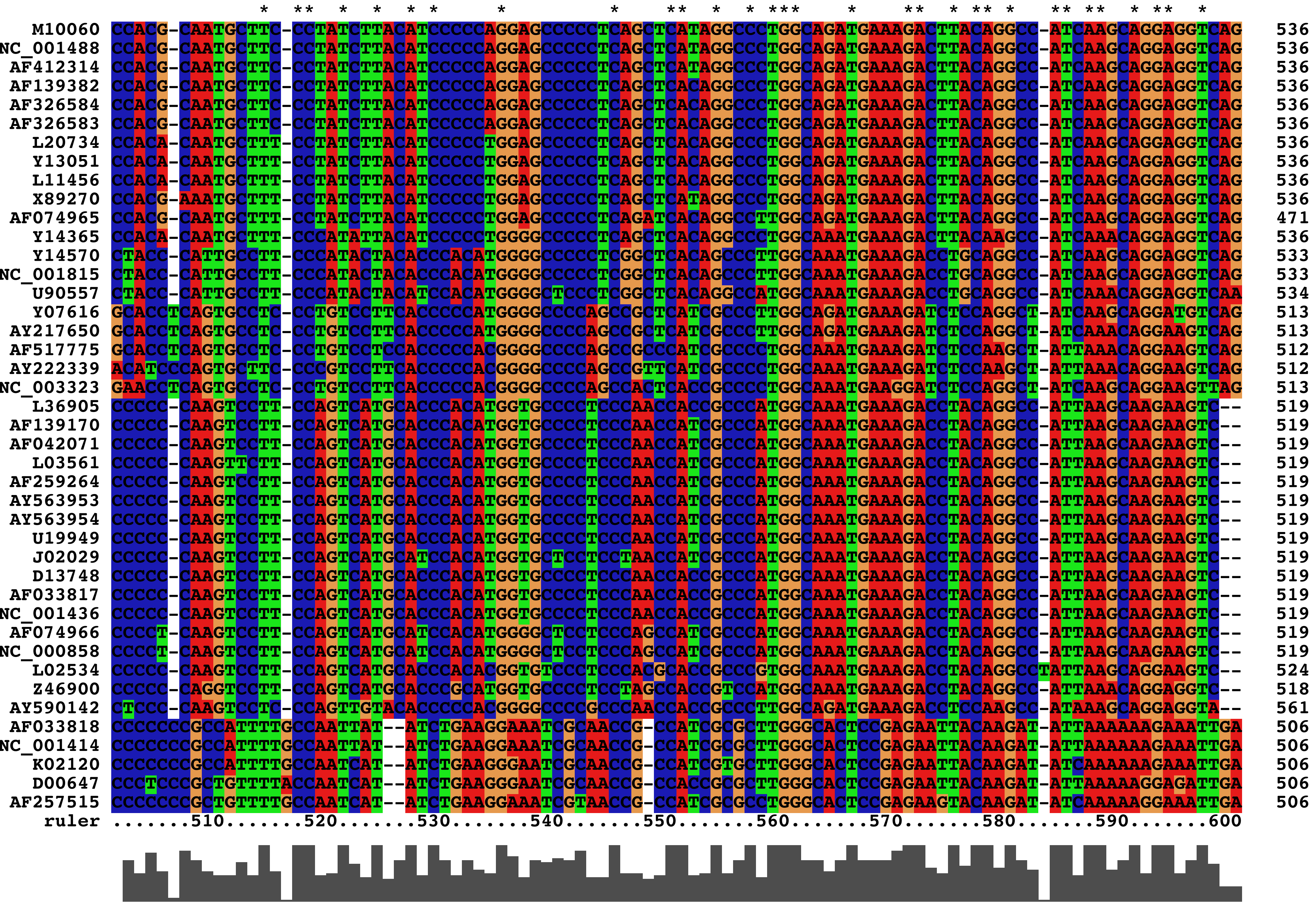
M10060 536
NC_001488 536
AF412314 536
AF139382 536
AF326584 536
AF326583 536
L20734 536
Y13051 536
L11456 536
X89270 536
AF074965 471
Y14365 536
Y14570 533
NC_001815 533
U90557 534
Y07616 513
AY217650 513
AF517775 512
AY222339 512
NC_003323 513
L36905 519
AF139170 519
AF042071 519
L03561 519
AF259264 519
AY563953 519
AY563954 519
U19949 519
J02029 519
D13748 519
AF033817 519
NC_001436 519
AF074966 519
NC_000858 519
L02534 524
Z46900 518
AY590142 561
AF033818 506
NC_001414 506
K02120 506
D00647 506
AF257515 506
ruler .......510.......520.......530.......540.......550.......560.......570.......580.......590.......600

```
                    ** *  **    * * * *    ** ** **  *  *  * **  *    **    ** ** **  *    **  *  *  * **    *
   M10060 CTCCT---CTGCTCTTGGCAGCCCCCAGTTC--ATGCAGACCCTCCGGCTGGCGGTACAACAGTTTGACCCCACCGCCAAGGACTTACAAGATCTCCTCC  631
NC_001488 CTCCT---CTGCTCTTGGCAGCCCCCAGTTC--ATGCAGACCCTCCGGCTGGCGGTACAACAGTTTGACCCCACCGCCAAGGACTTACAAGATCTCCTCC  631
 AF412314 CTCCT---CTGCTCCGGGCAGCCCCCAGTTC--ATGCAGACCCTCCGGCTGGCGGTACAACAGTTTGACCCCACCGCCAAGGACTTACAAGATCTCCTCC  631
 AF139382 CTCCT---CTGCTCCTGGCAGCCCCCAGTTC--ATGCAGACCCTCCGGCTGGCGGTACAACAGTTTGACCCCACTGCCAAGGACTTACAAGATCTCCTCC  631
 AF326584 CTCCT---CTGCTCCTGGCAGCCCCCAGTTC--ATGCAGACCCTCCGGCTGGCGGTACAACAGTTTGACCCCACTGCCAAGGACTTACAAGATCTCCTCC  631
 AF326583 CTCCT---CTGCTCCTGGCAGCCCCCAGTTC--ATGCAGACCCTCCGGCTGGCGGTACAACAGTTTGACCCCACTGCCAAGGACTTACAAGATCTCCTCC  631
   L20734 CTCCT---CTGCCCCTGGCAGCCCCCAGTTC--ATGCAGACCCTCCGGCTGGCGGTACAACAGTTTGACCCCACCGCCAAGGACTTACAAGATCTCCTCC  631
   Y13051 CTCCT---CTGCCCCTGGCAGCCCCCAGTTC--ATGCAGACCCTCCGGCTGGCGGTACAACAGTTTGACCCCACCGCCAAGGACTTACAAGATCTCCTCC  631
   L11456 CTCCT---CTGCCCCTGGCAGCCCCCAGTTC--ATGCAGACCCTCCGGCTGGCGGTACAACAGTTTGACCCCACCGCCAAGGACTTACAAGATCTCCTCC  631
   X89270 CTCCT---CTGCCCCTGGCAGCCCCCAGTTC--ATGCAGACCCTCCGGCTGGCGGTACAACAGTTTGACCCCACCGCCAAGGACTTACAAGATCTCCTCC  631
 AF074965 CTCCT---CTGCCCCTGGCAGCCCCCAGTTC--ATGCAGACCCTCCGGCTGGCGGTACAACAGTTTGACCCCACCGCCAAGGACTTACAAGATCTCCTCC  566
   Y14365 CTCCT---CTGCCCCTGGCAGCCCCCAATTC--ATGCAGACCCTCCGGCTGGCGGTACAACAGTTTGACCCCACCGCCAAGGATTTGCAAGATCTCCTCC  631
   Y14570 CACCT---CAGCCCCCGGGAGTCCCCAGTTC--ATGCAAACAGTCCGGCTCGCAATTCAGCAATTCGACCCCACGGCCAAAGACTTACAAGATCTCTTGC  628
NC_001815 CACCT---CAGCCCCCGGGAGTCCCCAGTTC--ATGCAAACAGTCCGGCTCGCAATTCAGCAATTCGACCCCACGGCCAAAGACTTACAAGATCTCTTGC  628
   U90557 TACCT---CGGCCCCTGGGAGTCCCCAGTTC--ATGCAAACAGTCCGGCTCGCAATTCAGCAATTCGACCCCACGGCCAAAGACTTACAAGATCTCTTGC  629
   Y07616 CTCTT---CCGCCCCTGGCAGCCTCCAATTC--ATGCAGACTGTCCGGCTAGCTGTTCAACAATTTGACCCCACTGCAAAAGATCTTCACGACCTCCTAC  608
 AY217650 CTCTT---CCGCCCCAGGCAGCCCCCAATTC--ATGCAGACTGTCCGCCTAGCCGTTCAGCAATTTGACCCCACTGCAAAAGATCTTCACGACCTCCTAC  608
 AF517775 CTCTT---CCGCCCCTGGTAGCCCCCAGTTC--ATGCAGACTGTGCGCTTAGCTGTCCAGCAATTTGACCCCACAGCAAAGGATCTCCACGATCTCCTAC  607
 AY222339 CTCTT---CCGCCCCTGGTAGCCCCCAGTTC--ATGCAGACTGTGCGCTTAGCTGTCCAGCAGTTTGACCCCACAGCAAAAGATCTCCACGATCTCCTAC  607
NC_003323 TTCTT---CCGCCCCTGGCAGCCCCCAGTTC--ATGCAGACCGTGCGTCTAGCTGTCCAGCAGTTTGACCCCACAGCTAAAGATCTCCATGATCTCCTAC  608
   L36905 -TCCCAAGCAGCCCCTGGGAGCCCCCAGTTT--ATGCAGACCATCCGGCTTGCGGTGCAGCAGTTTGACCCCACTGCCAAAGACCTCCAAGACCTCCTGC  616
 AF139170 -TCCCAAGCAGCCCCTGGGAGCCCCCAGTTT--ATGCAGACCATCCGGCTTGCGGTGCAGCAGTTTGACCCCACTGCCAAAGACCTCCAAGACCTCCTGC  616
 AF042071 -TCCCAAGCAGCCCCTGGGAGCCCCCAGTTT--ATGCAGACCATCCGGCTTGCGGTACAGCAGTTTGACCCCACTGCCAAAGACCTCCAAGACCTCCTGC  616
   L03561 -TCCCAAGCAGCCCCTGGGAGCCCCCAGTTT--ATGCAGACCATCCGGCTTGCGGTGCAGCAGTTTGACCCCACTGCCAAAGACCTCCAAGACCTCCTGC  616
 AF259264 -TCCCAAGCAGCCCCTGGGAGCCCCCAGTTT--ATGCAGACCATCCGGCTTGCGGTGCAGCAGTTTGACCCCACTGCCAAAGACCTCCAAGACCTCCTGC  616
 AY563953 -TCCCAAGCAGCCCCTGGGAGCCCCCAGTTT--ATGCAGACCATCCGGCTTGCGGTGCAGCAGTTTGACCCCACTGCCAAAGACCTCCAAGACCTCCTGC  616
 AY563954 -TCCCAAGCAGCCCCTGGGAGCCCCCAGTTT--ATGCAGACCATCCGGCTTGCGGTGCAGCAGTTTGACCCCACTGCCAAAGACCTCCAAGACCTCCTGC  616
   U19949 -TCCCAAGCAGCCCCTGGGAGCCCCCAGTTT--ATGCAGACCATCCGGCTTGCGGTGCAGCAGTTTGACCCCACTGCCAAAGACCTCCAAGACCTCCTGC  616
   J02029 -TCCCAAGCAGCCCCTGGGAGCCCCCAGTTT--ATGCAGACCATCCGGCTTGCGGTGCAGCAGTTTGACCCCACTGCCAAAGACCTCCAAGACCTCCTGC  616
   D13748 -TCCCAAGCGGCCCCTGGAAGCCCCCAGTTT--ATGCAGACCATCCGGCTTGCGGTGCAGCAGTTTGACCCCACTGCCAAAGACCTCCAAGACCTCCTGC  616
 AF033817 -TCCCAAGCGGCCCCTGGAAGCCCCCAGTTT--ATGCAGACCATCCGGCTTGCGGTGCAGCAGTTTGACCCCACTGCCAAAGACCTCCAAGACCTCCTGC  616
NC_001436 -TCCCAAGCGGCCCCTGGAAGCCCCCAGTTT--ATGCAGACCATCCGGCTTGCGGTGCAGCAGTTTGACCCCACTGCCAAAGACCTCCAAGACCTCCTGC  616
 AF074966 -TCCCAGGCAGCCCCCGGGAGCCCCCAGTTT--ATGCAGACCATCCGGCTTGCAGTGCAGCAGTTTGACCCCACTGCCAAAGACCTCCAAGACCTCCTAC  616
NC_000858 -TCCCAGGCAGCCCCCGGGAGCCCCCAGTTT--ATGCAGACCATCCGGCTTGCAGTGCAGCAGTTTGACCCCACTGCCAAAGACCTCCAAGACCTCCTAC  616
   L02534 -TCCCAAGCAGCCCCCGGGAGCCCCCAGTTT--ATGCAGACCATTCGGCTTGCAGTACAGCAGTTTGACCCCACTGCCAAGGACCTCCAGGACCTCCTGC  621
   Z46900 -TCCCAGGCAGCCCCTGGGAGCCCCCAGTTT--ATGCAAACTGTCAGGCTTGCGGTACAGCAGTTTGACCCCACTGCCAAGGACCTCCAGGACCTCCTGC  615
 AY590142 -TCCCAAGCCGCTCCGGGAAGCCCCCAGTTC--ATGCAAACCGTTAGGCTAGCGGTACAACAATTCGATCCCACTGCCAAGGATCTGCAGGATCTCCTGC  658
 AF033818 AAATAA---GGCACCGGGT--TCGCAAGTATGGATACAAACACTACGACTTGCAATCTTACAGGCCGACCCTACTCCTGCTGACCTAGAACAACTTTGCC  601
NC_001414 AAATAA---GGCACCGGGT--TCGCAAGTATGGATACAAACACTACGACTTGCAATCTTACAGGCCGACCCTACTCCTGCTGACCTAGAACAACTTTGCC  601
   K02120 AAATAA---GGCACCGGGT--TCGCAAGTATGGATACAAACACTACGACTTGCAATCCTGCAGGCCGACCCTACTCCGGCTGACCTAGAACAACTTTGCC  601
   D00647 AAATAA---GGCACCGGGG--TCGCAAGTATGGATACAAACGCTACGACTTGCAATCCTGCAGGCCGACCCTACCCCTGCTGACCTAGAACAGCTTTGCC  601
 AF257515 AAATAA---GGCACCGGGT--TCGCAAGTATGGATACAAACACTACGGCTTGCAATCCTGCAGGCCGACCCTACTCCTGCTGACCTAGAACAACTTTGCC  601
    ruler .......610.......620.......630.......640.......650.......660.......670.......680.......690.......700
```

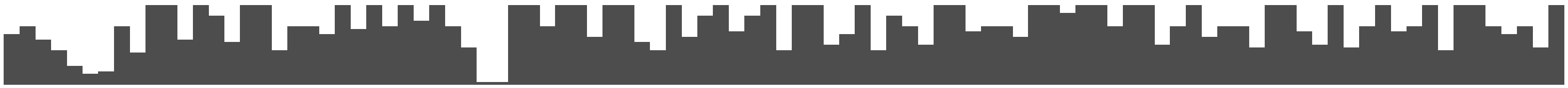

```
           * **  * ** *  **     *        *   *    *  *  * *   *          **  *     * **  *             * ** *  
M10060     AGTACCTATGCTCCTCCCTCGTAGTTT---C---CTTACACCATCAGCAGCTTAACACACTAATTACCGAGGCTGAGAC--CCGCGGGATGACAGGCTAC   723
NC_001488  AGTACCTATGCTCCTCCCTCGTAGTTT---C---CTTACACCATCAGCAGCTTAACACACTAATTACCGAGGCTGAGAC--CCGCGGGATGACAGGCTAC   723
AF412314   AGTACCTATGCTCCTCCCTCGTAGTTT---C---CTTACACCATCAGCAGCTTAACACACTAATTACCGAGGCTGAGAC--CCGCGGGATGACAGGCTAC   723
AF139382   AGTACCTATGCTCCTCCCTCGTAGTTT---C---CTTACACCATCAGCAGCTCAACACACTAATTACCGAAGCTGAGAC--CCGCGGGATGACAGGCTAC   723
AF326584   AGTACCTATGCTCCTCCCTCGTAGTTT---C---CTTACACCATCAGCAGCTCAACACACTAATTACCGAGGCTGAGAC--CCGCGGGATGACAGGCTAC   723
AF326583   AGTACCTATGCTCCTCCCTCGTAGTTT---C---CTTACACCATCAGCAGCTCAACACACTAATTACCGAGGCTGAGAC--CCGCGGGATGACAGGCTAC   723
L20734     AGTACCTATGCTCCTCCCTCGTGGTTT---C---CTTACACCATCAGCAGCTCAACACACTAATCACCGAGGCTGAGAC--TCGCGGGGTGACAGGCTAC   723
Y13051     AGTACCTATGCTCCTCCCTCGTGGTTT---C---CTTACACCATCAGCAGCTCAACACACTAATTACCGAGGCTGAGAC--TCGCGGGGTGACAGGCTAC   723
L11456     AGTACCTGTGCTCCTCCCTCGTGGTTT---C---CTTACACCATCAGCAGCTCAACACACTAATCACCGAGGCTGAGAC--TCGCGGGGTGACAGGCTAC   723
X89270     AGTACCTATGCTCCTCCCTCGTGGTTT---C---CTTACACCATCAGCAGCTCAACACACTAATCACCGAGGCTGAGAC--TCGCGGGGTGACAGGCTAC   723
AF074965   AGTACCTGTGCTCCTCCCTCGTGGTTT---C---CTTACACCATCAGCAGCTCAACACACTAATCACCGAGGCTGAGAC--TCGCGGGGTGACAGGCTAC   658
Y14365     AGTACCTATGCTCCTCTCTCGTGGTCT---C---CCTACACCATCAGCAGCTCAACACACTAATCACCGAGGCTGAGAC--CCGCGGGATGGCAGGCTAT   723
Y14570     AGTACCTCTGCTCCTCCCTAGTCGTCT---C---CCTTCACCATCAACAATTCCATACCCTGATTACCGAAGCTGAAAC--CCGGGGAATGACAGGTTAT   720
NC_001815  AGTACCTCTGCTCCTCCCTAGTCGTCT---C---CCTTCACCATCAACAATTCCATACCCTGATTACCGAAGCTGAAAC--CCGGGGAATGACAGGTTAT   720
U90557     AGTACCTCTGCTCCTCCCTAGTCGTCT---C---CCTTCACCATCAACAACTCCATACCCTAATTACCGAGGCTGAAAC--CAGGGGAATGACAGGTTAT   721
Y07616     AGTACCTATGCTCCTCACTAGTTGCCT---CTTACTTACATCATCAGCAGCTTGAGACCCTCATAGCTCAGGCAGAAAC--TCAAGGTATAACAGGATAC   703
AY217650   AGTACCTATGCTCCTCCCTAGTTGCCT---C---CTTACACCATCAGCAGCTTGAGACCCTCATAGCTCAGGCAGAAAC--TCAAGGTATAACAGGATAT   700
AF517775   AGTACCTATGCTCCTCATTAGTCGCCT---C---CCTGCACCATCAGCAGCTTGAGACCCTCATAGCTCAGGCAGAAAC--CCAAGGTATAACAGGATAT   699
AY222339   AGTACCTATGCTCCTCACTAGTTGCCT---C---CCTACACCATCAACAACTTGAGACCCTCATAGCCCAGGCAGAGAC--CCAAGGTATAACAGGGTAC   699
NC_003323  AGTACTTATGCTCCTCGCTAGTTACCT---C---CCTGCACCATCAGCAACTTGAGACTCTCATAGCCCAGGCAGAAAC--CCAAGGTATAACAGGCTAT   700
L36905     AGTACCTTTGCTCCTCCCTCGTGGCTT---C---CCTCCATCACCAGCAGCTAGATAGCCTTATATCAGAGGCCGAAAC--CCGAGGTATTACAGGTTAT   708
AF139170   AGTACCTTTGCTCCTCCCTCGTGGCTT---C---CCTCCATCACCAGCAGCTAGATAGCCTTATATCAGAGGCCGAAAC--CCGAGGTATTACAGGTTAT   708
AF042071   AGTACCTTTGCTCCTCCCTCGTGGCTT---C---CCTCCATCACCAGCAGCTAGATAGCCTTATATCAGAGGCCGAAAC--CCGAGGTATTACAGGTTAT   708
L03561     AGTACCTTTGCTCCTCCCTCGTGGCTT---C---CCTCCATCACCAGCAGCTAGATAGCCTTATATCAGAGGCCGAAAC--CCGAGGTATTACAGGTTAT   708
AF259264   AGTACCTTTGCTCCTCCCTCGTGGCTT---C---CCTCCATCACCAGCAGCTAGATAGCCTTATATCAGAGGCCGAAAC--CCGGGGTATTACAGGTTAT   708
AY563953   AGTACCTTTGCTCCTCCCTCGTGGCCT---C---CCTCCATCACCAGCAGCTAGATAGCCTTATATCAGAGGCCGAAAC--CCGAGGTATTACAGGTTAT   708
AY563954   AGTACCTTTGCTCCTCCCTCGTGGCCT---C---CCTCCATCACCAGCAGCTAGATAGCCTTATATCAGAGGCCGAAAC--CCGAGGTATTACAGGTTAT   708
U19949     AGTACCTTTGCTCCTCCCTCGTGGCTT---C---CCTCCATCACCAGCAGCTAGATAGCCTTATATCAGAGGCCGAAAC--CCGAGGTATTACAGGTTAT   708
J02029     AGTACCTTTGCTCCTCCCTCGTGGCTT---C---CCTCCATCACCAGCAGCTAGATAGCCTTATATCAGAGGCCGAAAC--CCGAGGTATTACAGGTTAT   708
D13748     AGTACCTTTGCTCCTCCCTCGTGGCTT---C---CCTCCATCACCAGCAGCTAGATAGCCTTATATCAGAGGCCGAAAC--TCGAGGTATTACAGGTTAT   708
AF033817   AGTACCTTTGCTCCTCCCTCGTGGCTT---C---CCTCCATCACCAGCAGCTAGATAGCCTTATATCAGAGGCCGAAAC--TCGAGGTATTACAGGTTAT   708
NC_001436  AGTACCTTTGCTCCTCCCTCGTGGCTT---C---CCTCCATCACCAGCAGCTAGATAGCCTTATATCAGAGGCCGAAAC--TCGAGGTATTACAGGTTAT   708
AF074966   AGTACCTTTGCTCCTCCCTCGTGGCCT---C---CCTCCATCACCAGCAGCTAGATAGCCTTATATCAGAGGCCGAAAC--TCGAGGTATTACAGGTTAT   708
NC_000858  AGTACCTTTGCTCCTCCCTCGTGGCCT---C---CCTCCATCACCAGCAGCTAGATAGCCTTATATCAGAGGCCGAAAC--TCGAGGTATTACAGGTTAT   708
L02534     AGTACCTTTGCTCCTCCCTAGTAGCTT---C---CCTCCATCACCAGCAGCTAGATAGCCTTATATCAGAGGCTGAGAC--TCGAGGTATTACAGGATAT   713
Z46900     AATACCTTTGCTCCTCCCTGGTGGCTT---C---CCTCCATCACCAACAGCTAGATAGCCTTATGTCAGAAGCCGAGAC--TCGAGGTATTACAGGCTAT   707
AY590142   AGTATCTATGTTCCTCCCTAATAGCCT---C---CCTCCACCATCAACAGCTAGATAGCCTCATCTCAGAGGCAGAAAC--CCGAGGAATTACCGGATAC   750
AF033818   AATATAT-TGCT--TCCCCGGTCGATCAAACG-GCCCACATGACCAGC--CTAACGGCAGCAATAGCCGCCGCTGAAGCGGCCAATACCCTCCAGGGTTT   695
NC_001414  AATATAT-TGCT--TCCCCGGTCGATCAAACG-GCCCACATGACCAGC--CTAACGGCAGCAATAGCCGCCGCTGAAGCGGCCAATACCCTCCAGGGTTT   695
K02120     AATATAT-TGCT--TCCCCGGTCGACCAAACG-GCCCATATGACCAGC--CTAACGGCAGCAATAGCCGCCGCTGAAGCGGC-AACACCCTCCAGGGTTT   694
D00647     AATATAT-TGCT--TCCCCGGTCGACCAAACG-GCCCACATGACCAGC--CTAACGGCGGCAATAGCCG---CTGAAGCGGC-AATACCCTCCAGGGTTT   691
AF257515   AATATAT-TGCT--TCCCCGGTCGACCAAACG-GCCCACATGACTAGC--CTAACGGCAGCAATAGCTGCCGCTGAAGCGGCCAACACCCTCCAGGGTTT   695
ruler      .......710.......720.......730.......740.......750.......760.......770.......780.......790.......800
```

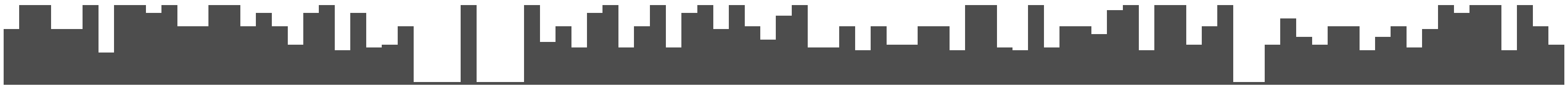

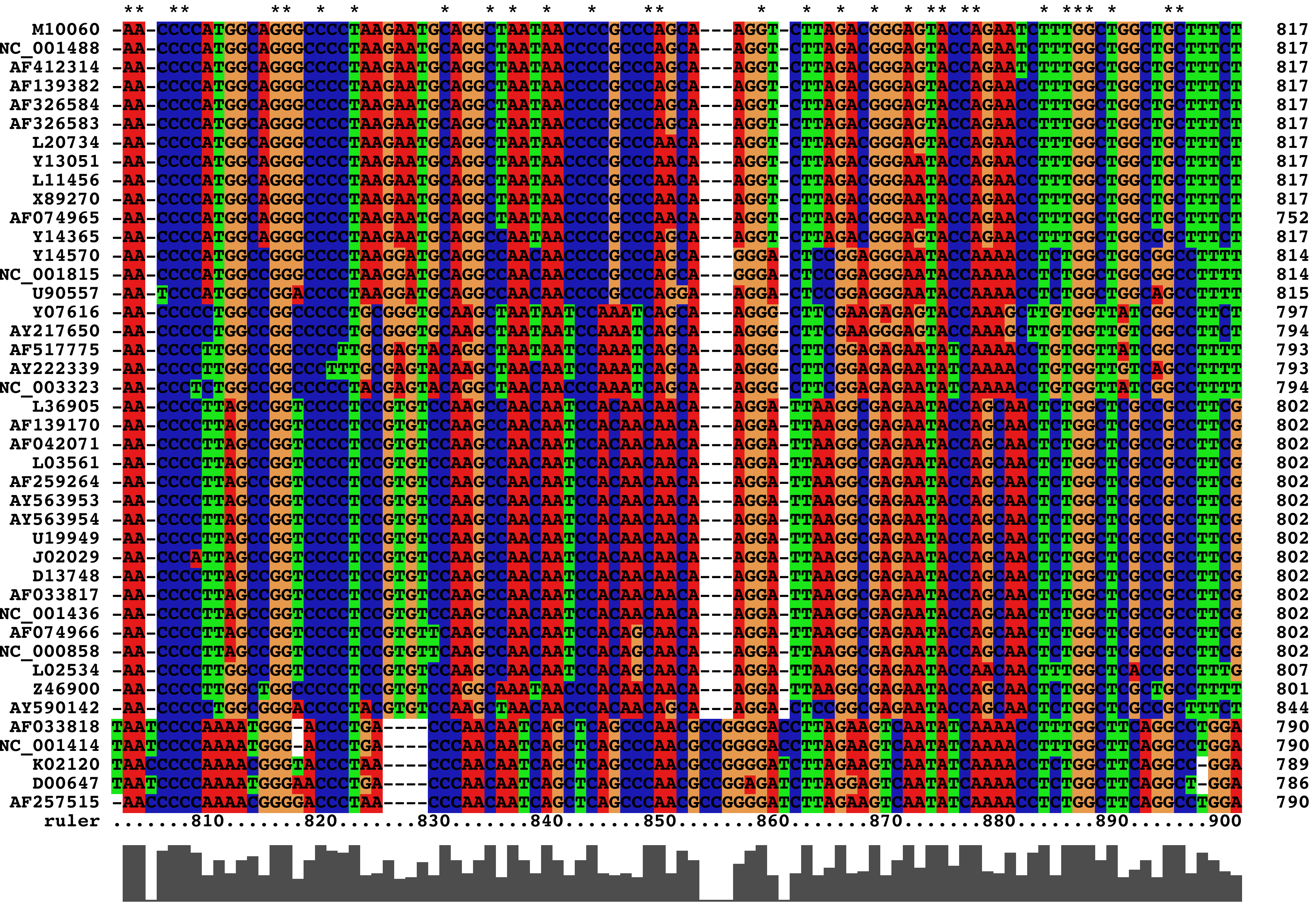
M10060 817
NC_001488 817
AF412314 817
AF139382 817
AF326584 817
AF326583 817
L20734 817
Y13051 817
L11456 817
X89270 817
AF074965 752
Y14365 817
Y14570 814
NC_001815 814
U90557 815
Y07616 797
AY217650 794
AF517775 793
AY222339 793
NC_003323 794
L36905 802
AF139170 802
AF042071 802
L03561 802
AF259264 802
AY563953 802
AY563954 802
U19949 802
J02029 802
D13748 802
AF033817 802
NC_001436 802
AF074966 802
NC_000858 802
L02534 807
Z46900 801
AY590142 844
AF033818 790
NC_001414 790
K02120 789
D00647 786
AF257515 790
ruler .......810.......820.......830.......840.......850.......860.......870.......880.......890.......900

```
             *  **         *     *     *     *  ***  *    *  ***  *  **  **  *    *          * *   *            **   *    *    *    *    **   *
M10060     CCACCCTGCCAGGCAATACCCGTG-ACCCCTCTTGGGCAGCTATCCTACAGGGGCTGG--AGGAACCCTATTGCGCGTTCGTAGAGCGCCT-TAACGTGG   913
NC_001488  CCACCCTGCCAGGCAATACCCGTG-ACCCCTCTTGGGCAGCTATCCTACAGGGGCTGG--AGGAACCCTATTGCGCGTTCGTAGAGCGCCT-TAACGTGG   913
AF412314   CCACCCTGCCAGGCAATACCCGTG-ACCCCTCTTGGGCAGCTATCCTACAGGGGCTGG--AGGAACCCTATTGCGCGTTCGTAGAGCGCCT-TAACGTGG   913
AF139382   CCACCCTGCCAGGCAATACCCGTG-ACCCCTCTTGGGCAGCTATCCTACAGGGGCTGG--AGGAACCCTATTGCGCGTTCGTAAAGCGCCT-TAACGTGG   913
AF326584   CCACCCTGCCAGGCAATACCCGTG-ACCCCTCTTGGGCAGCTATCCTACAGGGGCTGG--AGGAACCCTATTGCGCGTTCGTAGAGCGCCT-TAACGTGG   913
AF326583   CCACCCTGCCAGGCAATACCCGCG-ACCCCTCTTGGGCAGCTATCCTACAGGGGCTGG--AGGAACCCTATTGCGCGTTCGTAGAGCGCCT-TAACGTGG   913
L20734     CCACCCTTCCAGGCAATACCCGTG-ACCCCTCTTGGGCGGCTATCCTACAGGGGCTGG--AAGAACCCTACTGCGCGTTCGTAGAGCGCCT-TAATGTGG   913
Y13051     CCACCCTTCCAGGCAATACCCGTG-ACCCCTCTTGGGCGGCTATCCTACAGGGGCTGG--AAGAACCCTACTGCGCGTTCGTAGAGCGCCT-TAATGTGG   913
L11456     CCACCCTTCCAGGCAATACCCGTG-ACCCCTCTTGGGCGGCTATCCTACAAGGGCTGG--AAGAACCCTATTGCGCGTTCGTAGAGCGCCT-TAATGTGG   913
X89270     CCACCCTTCCAGGCAATACCCGTG-ACCCCTCTTGGGCAGCTATCCTACAGGGGCTGG--AGGAACCCTACTGCGCGTTCGTAGAGCGCCT-TAATGTGG   913
AF074965   CCACCCTTCCAGGCAATACCCGTG-ACCCCTCTTGGGCGGCTATCCTACAAGGGCTGG--AAGAACCCTATTGCGCGTTCGTAGAGCGCCT-TAATGTGG   848
Y14365     CCACCCTGCCAGGCAACACCCGTG-ATCCCTCTTGGGCGGCTATCCTACAGGGGCTGG--AGGAACCCTATTGCGCGTTCGTAGAACGCCT-TAATGTGG   913
Y14570     CAGCTCTGCCAGGGAACACCCGCG-ACCCATCCTGGGCAGCAATCTTGCAAGGGCTAG--AGGAACCCTACTGCGCTTTCGTAGAACGCCT-TAATGTTG   910
NC_001815  CAGCTCTGCCAGGGAACACCCGCG-ACCCATCCTGGGCAGCAATCTTGCAAGGGCTAG--AGGAACCCTACTGCGCTTTCGTAGAACGCCT-TAATGTTG   910
U90557     CGGCTCTGCCAGGGAACACCCGCG-ACCCATCTTGGGCGGCAATCTTGCAAGGGCTAG--AAGAACCCTATTGCGCCTTGCTAGAGCGCCT-TAATGTTG   911
Y07616     CCGCTCTCCCGGGGAACACCAAGG-ACCCCACCTGGGCGGCTATCCTCCAAGGTCCTG--AGGAGCCCTTTTGTTGTTTTGTAGAAAGGCT-CAATGTGG   893
AY217650   CCGCTCTCCCCGGGAACACCAAGG-ACCCCACCTGGGCGGCTATCCTCCAAGGTCCTG--AGGAGCCCTTTTGCTCTTTTGTGGAAAGGCT-CAATGTGG   890
AF517775   CCGCACTCCCAGGGAACACTAAGG-ACCCCACTTGGGCAGCTATCCTCCAAGGACCTG--AGGAACCCTTTTGCTCCTTTGTAGAAAGGCT-CAATATAG   889
AY222339   CCGCACTCCCGGGGAACACAAAGG-ACCCCACCTGGGCAGCTATCCTTCAAGGACCTG--AGGAGCCCTTCTGCTCTTTTGTAGAAAGACT-CAATGTGG   889
NC_003323  CCGCCCTCCCGGGAAACACCAAGG-ACCCCACCTGGGCGGCCATCCTCCAAGGACCTG--AAGAACCTTTCTGCTCTTTCGTGGAGAGGCT-TAATGTGG   890
L36905     CCGCCCTGCCAGGGAGTGCCAAAG-ACCCTTCCTGGGCCTCTATCCTCCAAGGCCTGG--AGGAGCCTTACCACGCCTTCGTAGAACGCCT-CAACATAG   898
AF139170   CCGCCCTGCCAGGGAGTGCCAAAG-ACCCTTCCTGGGCCTCTATCCTCCAAGGCCTGG--AGGAGCCTTACCACGCCTTCGTAGAACGCCT-CAACATAG   898
AF042071   CCGCCCTGCCAGGGAGTGCCAAAG-ACCCTTCCTGGGCCTCTATCCTCCAAGGCCTGG--AGGAGCCTTACCACGCCTTCGTAGAACGCCT-CAACATAG   898
L03561     CCGCCCTGCCAGGGAGTGCCAAAG-ACCCTTCCTGGGCCTCTATCCTCCAAGGCCTGG--AGGAGCCTTACCACGCCTTCGTAGAACGCCT-CAACATAG   898
AF259264   CCGCCCTGCCAGGGAGTGCCAAAG-ACCCTTCCTGGGCCTCTATCCTCCAAGGCCTGG--AGGAGCCTTACCACGCCTTCGTAGAACGCCT-CAACATAG   898
AY563953   CCGCCCTGCCAGGGAGTGCCAAAG-ACCCTTCCTGGGCCTCTATCCTCCAAGGCCTGG--AGGAGCCTTACCACGCCTTCGTAGAACGCCT-CAACATAG   898
AY563954   CCGCCCTGCCAGGGAGTGCCAAAG-ACCCTTCCTGGGCCTCTATCCTCCAAGGCCTGG--AGGAGCCTTACCACGCCTTCGTAGAACGCCT-CAACATAG   898
U19949     CCGCCCTGCCGGGGAGTGCTAAAG-ACCCTTCCTGGGCCTCTATCCTCCAAGGCCTGG--AGGAGCCTTACCACGCCTTCGTAGAACGCCT-CAACATAG   898
J02029     CCGCCCTGCCGGGGAGTGCCAAAG-ACCCTTCCTGGGCCTCTATCCTCCAAGGCCTGG--AGGAGCCTTACCACGCCTTCGTAGAACGCCT-CAACATAG   898
D13748     CCGCCCTGCCAGGGAGTGCCAAAG-ACCCTTCCTGGGCCTCTATCCTCCAAGGCCTGG--AGGAGCCTTACCACGCCTTCGTAGAACGCCT-CAACATAG   898
AF033817   CCGCCCTGCCAGGGAGTGCCAAAG-ACCCTTCCTGGGCCTCTATCCTCCAAGGCCTGG--AGGAGCCTTACCACGCCTTCGTAGAACGCCT-CAACATAG   898
NC_001436  CCGCCCTGCCAGGGAGTGCCAAAG-ACCCTTCCTGGGCCTCTATCCTCCAAGGCCTGG--AGGAGCCTTACCACGCCTTCGTAGAACGCCT-CAACATAG   898
AF074966   CCGCCCTACCAGGGAGTGCCAAAG-ACCCTTCCTGGGCCTCTATCCTCCAAGGCCTGG--AGGAGCCTTACCACACCTTCGTAGAACGTCT-CAACGTAG   898
NC_000858  CCGCCCTACCAGGGAGTGCCAAAG-ACCCTTCCTGGGCCTCTATCCTCCAAGGCCTGG--AGGAGCCTTACCACACCTTCGTAGAACGTCT-CAACGTAG   898
L02534     CCGCCCTGCCTGGGAGTGCCAAAG-ACCCTTCCTGGGCCTCCATCCTCCAAGGCCTAG--AGGAGCCTTACCACACCTTCGTAGAACGCCT-CAACGTAG   903
Z46900     CCGCCCTGCCAGGGAGCGCTAAAG-ACCCTTCCTGGGCCTCTATCCTCCAAGGCCTGG--AAGAGCCTTACCACGCCTTTGTAGAACGCCT-CAATGTAG   897
AY590142   CTGCCTTGCCAGGGAGTGCCAAAG-ACCCCTCATGGGCCTCCATCCTTCAGGGTCTAG--AAGAACCCTATCACTCTTTTGTGGAGCGCCT-CAATGTAG   940
AF033818   AAAATCTCCCTACTCGTCCTTCAG-TACAACCCTGGTCCACCATCGTCCAAGGCCCCGCCGAGAGCTATGTAGAG--TTTGTCAACCGGTTACAA-ATTT   886
NC_001414  AAAATCTCCCTACTCGTCCTTCAG-TACAACCCTGGTCCACCATCGTCCAAGGCCCCGCCGAGAGCTATGTAGAG--TTTGTCAACCGGTTACAA-ATTT   886
K02120     AAAATCTCCCTACTCGTCCTTCAGCTACAACCTTGGTCCACCATCGTCCAAGGCCCCGCCGAAAGCTCTGTAGAG--TTTGTCAACCGGTTACAA-ATTT   886
D00647     AGAATCTCCCTACTCGTCCTTCAGGTACAACCCTGGTCCACCATCGTCCAGGGCCCCGCCGAGAGCTATGTAGAG--TTTGTCAACCGGTTACAA-ATTT   883
AF257515   AAAATCTCCCTACTCGGCCTTCAG-TCCAACCCTGGTCCACCATCGTCCAAGGCCCCGCCGAAAGCTATGTAGAA--TTTGTCAACCGGTTACAA-ATTT   886
ruler      .......910.......920.......930.......940.......950.......960.......970.......980.......990.......1000
```

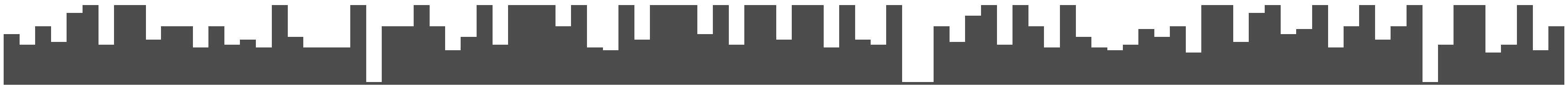

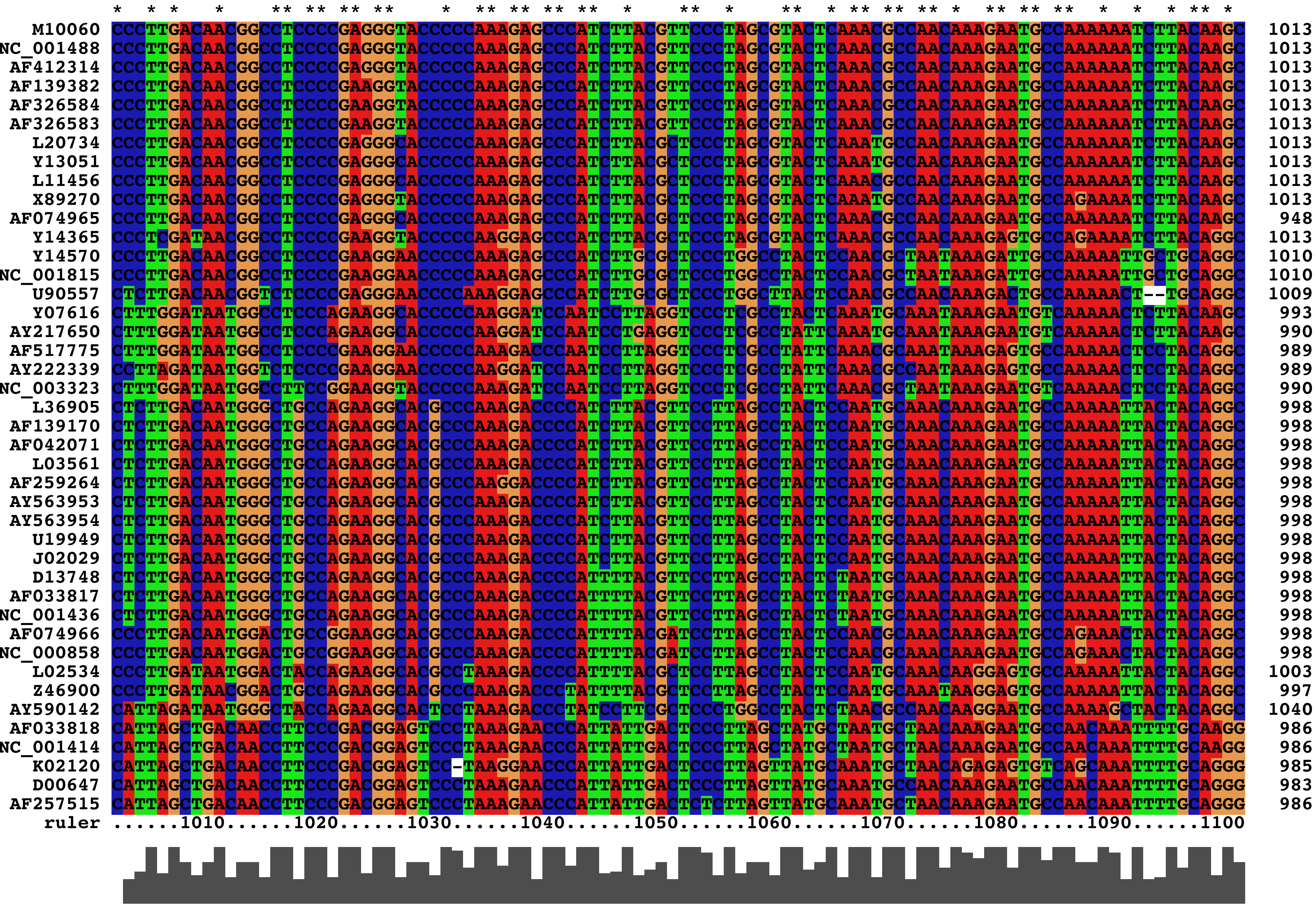
*  * *   *    ** ** ** **     *  ** ** ** **  *     **   *     **   * ** ** ** *   ** ** **   *   *   * ** *
M10060 CCCTTGACAACGGCCTCCCCGAGGGTACCCCCAAAGAGCCCATCTTACGTTCCCTAGCGTACTCAAACGCCAACAAAGAATGCCAAAAAATCTTACAAGC 1013
NC_001488 CCCTTGACAACGGCCTCCCCGAGGGTACCCCCAAAGAGCCCATCTTACGTTCCCTAGCGTACTCAAACGCCAACAAAGAATGCCAAAAAATCTTACAAGC 1013
AF412314 CCCTTGACAACGGCCTCCCCGAGGGTACCCCCAAAGAGCCCATCTTACGTTCCCTAGCGTACTCAAACGCCAACAAAGAATGCCAAAAAATCTTACAAGC 1013
AF139382 CCCTTGACAACGGCCTCCCCGAAGGTACCCCCAAAGAGCCCATCTTACGTTCCCTAGCGTACTCAAACGCCAACAAAGAATGCCAAAAAATCTTACAAGC 1013
AF326584 CCCTTGACAACGGCCTCCCCGAAGGTACCCCCAAAGAGCCCATCTTACGTTCCCTAGCGTACTCAAACGCCAACAAAGAATGCCAAAAAATCTTACAAGC 1013
AF326583 CCCTTGACAACGGCCTCCCCGAAGGTACCCCCAAAGAGCCCATCTTACGTTCCCTAGCGTACTCAAACGCCAACAAAGAATGCCAAAAAATCTTACAAGC 1013
L20734 CCCTTGACAACGGCCTCCCCGAGGGCACCCCCAAAGAGCCCATCTTACGCTCCCTAGCGTACTCAAATGCCAACAAAGAATGCCAAAAAATCTTACAAGC 1013
Y13051 CCCTTGACAACGGCCTCCCCGAGGGCACCCCCAAAGAGCCCATCTTACGCTCCCTAGCGTACTCAAATGCCAACAAAGAATGCCAAAAAATCTTACAAGC 1013
L11456 CCCTTGACAACGGCCTCCCCGAGGGCACCCCCAAAGAGCCCATCTTACGCTCCCTAGCGTACTCAAACGCCAACAAAGAATGCCAAAAAATCTTACAAGC 1013
X89270 CCCTTGACAACGGCCTCCCCGAGGGTACCCCCAAAGAGCCCATCTTACGCTCCCTAGCGTACTCAAATGCCAACAAAGAATGCCAGAAAATCTTACAAGC 1013
AF074965 CCCTTGACAACGGCCTCCCCGAGGGCACCCCCAAAGAGCCCATCTTACGCTCCCTAGCGTACTCAAACGCCAACAAAGAATGCCAAAAAATCTTACAAGC 948
Y14365 CCCTCGATAACGGCCTCCCCGAAGGTACCCCCAAGGAGCCCATCTTACGCTCCCTAGCGTACTCAAACGCCAACAAAGAGTGCCAGAAAATCTTACAGGC 1013
Y14570 CCCTTGACAACGGCCTCCCCGAAGGAACCCCCAAAGAGCCCATCTTGCGCTCCCTGGCCTACTCCAACGCTAATAAAGATTGCCAAAAATTGCTGCAGGC 1010
NC_001815 CCCTTGACAACGGCCTCCCCGAAGGAACCCCCAAAGAGCCCATCTTGCGCTCCCTGGCCTACTCCAACGCTAATAAAGATTGCCAAAAATTGCTGCAGGC 1010
U90557 CTCTTGACAACGGTCTCCCCGAGGGAACCCCAAAGGAGCCCATCTTGCGCTCCCTGGCTTACTCCAACGCCAACAAAGACTGCCAAAAACT--TGCAGGC 1009
Y07616 CTTTGGATAATGGCCTCCCAGAAGGCACCCCCAAGGATCCAATCCTTAGGTCCCTCGCCTACTCAAATGCAAATAAAGAATGTCAAAAACTCTTACAAGC 993
AY217650 CTTTGGATAATGGCCTCCCAGAAGGCACCCCCAAGGATCCAATCCTGAGGTCCCTCGCCTATTCAAATGCAAATAAAGAATGTCAAAAACTCTTACAAGC 990
AF517775 CTTTGGATAATGGCCTCCCCGAAGGAACCCCCAAAGACCCAATCCTTAGGTCCCTCGCCTATTCAAACGCAAATAAAGAGTGCCAAAAACTCCTACAGGC 989
AY222339 CCTTAGATAATGGTCTCCCCGAAGGAACCCCCAAGGATCCAATCCTTAGGTCCCTCGCCTATTCAAACGCCAATAAAGAGTGCCAAAAACTCCTACAGGC 989
NC_003323 CTTTGGATAATGGCCTTCCGGAAGGTACCCCCAAAGATCCAATCCTTAGGTCCCTCGCCTATTCAAACGCTAATAAAGAATGTCAAAAACTCCTACAGGC 990
L36905 CTCTTGACAATGGGCTGCCAGAAGGCACGCCCAAAGACCCCATCTTACGTTCCTTAGCCTACTCCAATGCAAACAAAGAATGCCAAAAATTACTACAGGC 998
AF139170 CTCTTGACAATGGGCTGCCAGAAGGCACGCCCAAAGACCCCATCTTACGTTCCTTAGCCTACTCCAATGCAAACAAAGAATGCCAAAAATTACTACAGGC 998
AF042071 CTCTTGACAATGGGCTGCCAGAAGGCACGCCCAAAGACCCCATCTTACGTTCCTTAGCCTACTCCAATGCAAACAAAGAATGCCAAAAATTACTACAGGC 998
L03561 CTCTTGACAATGGGCTGCCAGAAGGCACGCCCAAAGACCCCATCTTACGTTCCTTAGCCTACTCCAATGCAAACAAAGAATGCCAAAAATTACTACAGGC 998
AF259264 CTCTTGACAATGGGCTGCCAGAAGGCACGCCCAAGGACCCCATCTTACGTTCCTTAGCCTACTCCAATGCAAACAAAGAATGCCAAAAATTACTACAGGC 998
AY563953 CTCTTGACAATGGGCTGCCAGAAGGCACGCCCAAAGACCCCATCTTACGTTCCTTAGCCTACTCCAATGCAAACAAAGAATGCCAAAAATTACTACAGGC 998
AY563954 CTCTTGACAATGGGCTGCCAGAAGGCACGCCCAAAGACCCCATCTTACGTTCCTTAGCCTACTCCAATGCAAACAAAGAATGCCAAAAATTACTACAGGC 998
U19949 CTCTTGACAATGGGCTGCCAGAAGGCACGCCCAAAGACCCCATCTTACGTTCCTTAGCCTACTCCAATGCAAACAAAGAATGCCAAAAATTACTACAGGC 998
J02029 CTCTTGACAATGGGCTGCCAGAAGGCACGCCCAAAGACCCCATCTTACGTTCCTTAGCCTACTCCAATGCAAACAAAGAATGCCAAAAATTACTACAGGC 998
D13748 CTCTTGACAATGGGCTGCCAGAAGGCACGCCCAAAGACCCCATTTTACGTTCCTTAGCCTACTCTAATGCAAACAAAGAATGCCAAAAATTACTACAGGC 998
AF033817 CTCTTGACAATGGGCTGCCAGAAGGCACGCCCAAAGACCCCATTTTACGTTCCTTAGCCTACTCTAATGCAAACAAAGAATGCCAAAAATTACTACAGGC 998
NC_001436 CTCTTGACAATGGGCTGCCAGAAGGCACGCCCAAAGACCCCATTTTACGTTCCTTAGCCTACTCTAATGCAAACAAAGAATGCCAAAAATTACTACAGGC 998
AF074966 CCCTTGACAATGGACTGCCGGAAGGCACGCCCAAAGACCCCATTTTACGATCCTTAGCCTACTCCAACGCAAACAAAGAATGCCAGAAACTACTACAGGC 998
NC_000858 CCCTTGACAATGGACTGCCGGAAGGCACGCCCAAAGACCCCATTTTACGATCCTTAGCCTACTCCAACGCAAACAAAGAATGCCAGAAACTACTACAGGC 998
L02534 CCCTTGATAATGGACTACCAGAAGGCACGCCTAAAGACCCCATTTTACGCTCCTTAGCCTACTCCAATGCAAACAAGGAGTGCCAAAAATTACTACAGGC 1003
Z46900 CCCTTGATAACGGACTGCCAGAAGGCACGCCCAAAGACCCTATTTTACGCTCCTTAGCCTACTCCAATGCAAATAAGGAGTGCCAAAAATTACTACAGGC 997
AY590142 CATTAGATAATGGGCTACCAGAAGGCACTCCTAAAGACCCTATCCTTCGCTCCCTGGCCTACTCTAACGCCAACAAGGAATGCCAAAAGCTACTACAGGC 1040
AF033818 CATTAGCTGACAACCTTCCCGACGGAGTCCCTAAAGAACCCATTATTGACTCCCTTAGCTATGCTAATGCTAACAAAGAATGCCAACAAATTTTGCAAGG 986
NC_001414 CATTAGCTGACAACCTTCCCGACGGAGTCCCTAAAGAACCCATTATTGACTCCCTTAGCTATGCTAATGCTAACAAAGAATGCCAACAAATTTTGCAAGG 986
K02120 CATTAGCTGACAACCTTCCCGACGGAGTCC-TAAGGAACCCATTATTGACTCCCTTAGTTATGCAAATGCTAACAGAGAGTGTCAGCAAATTTTGCAGGG 985
D00647 CATTAGCTGACAACCTTCCCGACGGAGTCCCTAAAGAACCCATTATTGACTCCCTTAGTTATGCAAATGCCAACAAAGAATGCCAACAAATTTTGCAGGG 983
AF257515 CATTAGCTGACAACCTTCCCGACGGAGTCCCTAAAGAACCCATTATTGACTCTCTTAGTTATGCAAATGCTAACAAAGAATGCCAACAAATTTTGCAGGG 986
ruler ......1010......1020......1030......1040......1050......1060......1070......1080......1090......1100

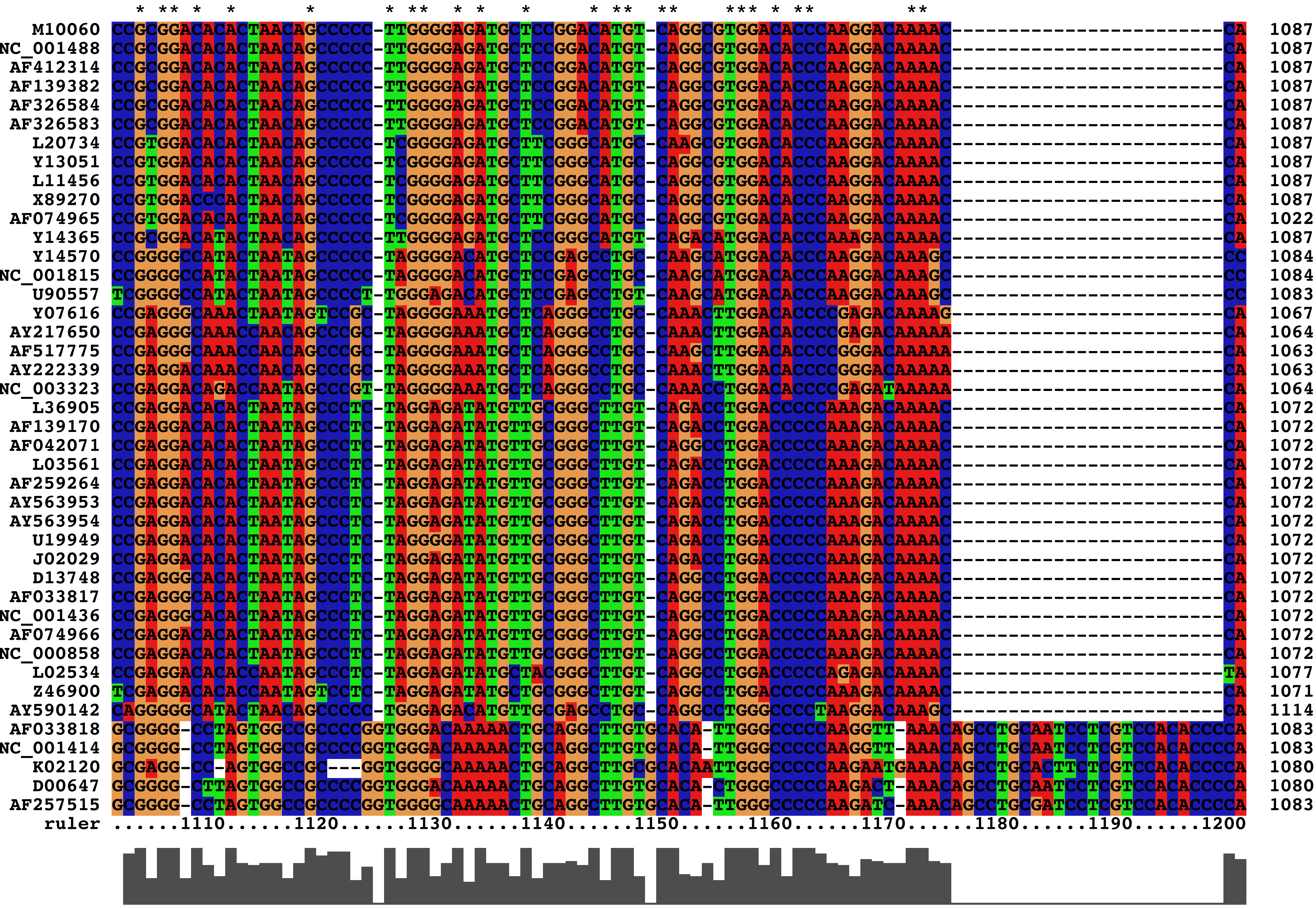

  *.**.*..*......*......*.**..*.*...*.....*.**..**....***.*.**........**
M10060 CCGCGGACACACTAACAGCCCCC-TTGGGGAGATGCTCCGGACATGT-CAGGCGTGGACACCCAAGGACAAAAC------------------------CA 1087
NC_001488 CCGCGGACACACTAACAGCCCCC-TTGGGGAGATGCTCCGGACATGT-CAGGCGTGGACACCCAAGGACAAAAC------------------------CA 1087
AF412314 CCGCGGACACACTAACAGCCCCC-TTGGGGAGATGCTCCGGACATGT-CAGGCGTGGACACCCAAGGACAAAAC------------------------CA 1087
AF139382 CCGCGGACACACTAACAGCCCCC-TTGGGGAGATGCTCCGGACATGT-CAGGCGTGGACACCCAAGGACAAAAC------------------------CA 1087
AF326584 CCGCGGACACACTAACAGCCCCC-TTGGGGAGATGCTCCGGACATGT-CAGGCGTGGACACCCAAGGACAAAAC------------------------CA 1087
AF326583 CCGCGGACACACTAACAGCCCCC-TTGGGGAGATGCTCCGGACATGT-CAGGCGTGGACACCCAAGGACAAAAC------------------------CA 1087
L20734 CCGTGGACACACTAACAGCCCCC-TCGGGGAGATGCTTCGGGCATGC-CAAGCGTGGACACCCAAGGACAAAAC------------------------CA 1087
Y13051 CCGTGGACACACTAACAGCCCCC-TCGGGGAGATGCTTCGGGCATGC-CAGGCGTGGACACCCAAGGACAAAAC------------------------CA 1087
L11456 CCGTGGACACACTAACAGCCCCC-TCGGGGAGATGCTTCGGGCATGC-CAGGCGTGGACACCCAAGGACAAAAC------------------------CA 1087
X89270 CCGTGGACCCACTAACAGCCCCC-TCGGGGAGATGCTTCGGGCATGC-CAGGCGTGGACACCCAAGGACAAAAC------------------------CA 1087
AF074965 CCGTGGACACACTAACAGCCCCC-TCGGGGAGATGCTTCGGGCATGC-CAGGCGTGGACACCCAAGGACAAAAC------------------------CA 1022
Y14365 CCGCGGACATACTAACAGCCCCC-TTGGGGAGATGCTCCGGGCATGT-CAGACATGGACACCCAAAGACAAAAC------------------------CA 1087
Y14570 CCGGGGCCATACTAATAGCCCCC-TAGGGGACATGCTCCGAGCCTGC-CAAGCATGGACACCCAAGGACAAAGC------------------------CC 1084
NC_001815 CCGGGGCCATACTAATAGCCCCC-TAGGGGACATGCTCCGAGCCTGC-CAAGCATGGACACCCAAGGACAAAGC------------------------CC 1084
U90557 TCGGGGCCATACTAATAGCCCCT-TGGGAGACATGCTCCGAGCCTGT-CAAGCATGGACACCCAAGGACAAAGC------------------------CC 1083
Y07616 CCGAGGGCAAACTAATAGTCCGC-TAGGGGAAATGCTCAGGGCCTGC-CAAACTTGGACACCCCGAGACAAAAG------------------------CA 1067
AY217650 CCGAGGGCAAACCAACAGCCCGC-TAGGGGAAATGCTCAGGGCCTGC-CAAACTTGGACACCCCGAGACAAAAA------------------------CA 1064
AF517775 CCGAGGGCAAACCAACAGCCCGC-TAGGGGAAATGCTCAGGGCCTGC-CAAGCTTGGACACCCCGGGACAAAAA------------------------CA 1063
AY222339 CCGAGGACAAACCAACAGCCCGC-TAGGGGAAATGCTCAGGGCCTGC-CAAACTTGGACACCCCGGGACAAAAA------------------------CA 1063
NC_003323 CCGAGGACAGACCAATAGCCCGT-TAGGGGAAATGCTCAGGGCCTGC-CAAACCTGGACACCCCGAGATAAAAA------------------------CA 1064
L36905 CCGAGGACACACTAATAGCCCTC-TAGGAGATATGTTGCGGGCTTGT-CAGACCTGGACCCCCAAAGACAAAAC------------------------CA 1072
AF139170 CCGAGGACACACTAATAGCCCTC-TAGGAGATATGTTGCGGGCTTGT-CAGACCTGGACCCCCAAAGACAAAAC------------------------CA 1072
AF042071 CCGAGGACACACTAATAGCCCTC-TAGGAGATATGTTGCGGGCTTGT-CAGGCCTGGACCCCCAAAGACAAAAC------------------------CA 1072
L03561 CCGAGGACACACTAATAGCCCTC-TAGGAGATATGTTGCGGGCTTGT-CAGACCTGGACCCCCAAAGACAAAAC------------------------CA 1072
AF259264 CCGAGGACACACTAATAGCCCTC-TAGGAGATATGTTGCGGGCTTGT-CAGACCTGGACCCCCAAAGACAAAAC------------------------CA 1072
AY563953 CCGAGGACACACTAATAGCCCTC-TAGGAGATATGTTGCGGGCTTGT-CAGACCTGGACCCCCAAAGACAAAAC------------------------CA 1072
AY563954 CCGAGGACACACTAATAGCCCTC-TAGGAGATATGTTGCGGGCTTGT-CAGACCTGGACCCCCAAAGACAAAAC------------------------CA 1072
U19949 CCGAGGACACACTAATAGCCCTC-TAGGGGATATGTTGCGGGCTTGT-CAGACCTGGACCCCCAAAGACAAAAC------------------------CA 1072
J02029 CCGAGGACACACTAATAGCCCTC-TAGGAGATATGTTGCGGGCTTGT-CAGACCTGGACCCCCAAAGACAAAAC------------------------CA 1072
D13748 CCGAGGGCACACTAATAGCCCTC-TAGGAGATATGTTGCGGGCTTGT-CAGGCCTGGACCCCCAAAGACAAAAC------------------------CA 1072
AF033817 CCGAGGGCACACTAATAGCCCTC-TAGGAGATATGTTGCGGGCTTGT-CAGGCCTGGACCCCCAAAGACAAAAC------------------------CA 1072
NC_001436 CCGAGGGCACACTAATAGCCCTC-TAGGAGATATGTTGCGGGCTTGT-CAGGCCTGGACCCCCAAAGACAAAAC------------------------CA 1072
AF074966 CCGAGGACACACTAATAGCCCTC-TAGGAGATATGTTGCGGGCTTGT-CAGGCCTGGACCCCCAAAGACAAAAC------------------------CA 1072
NC_000858 CCGAGGACACACTAATAGCCCTC-TAGGAGATATGTTGCGGGCTTGT-CAGGCCTGGACCCCCAAAGACAAAAC------------------------CA 1072
L02534 CCGAGGACACACCAATAGCCCTC-TAGGAGATATGCTACGGGCTTGT-CAGGCCTGGACCCCCAGAGACAAAAC------------------------TA 1077
Z46900 TCGAGGACACACCAATAGTCCTC-TAGGAGATATGCTGCGGGCTTGT-CAGGCCTGGACCCCCAAAGACAAAAC------------------------CA 1071
AY590142 CAGGGGGCATACTAACAGCCCCC-TGGGAGACATGTTGCGAGCCTGC-CAGGCCTGGGCCCCTAAGGACAAAGC------------------------CA 1114
AF033818 GCGGGG-CCTAGTGGCCGCCCCGGTGGGACAAAAACTGCAGGCTTGTGCACA-TTGGGCCCCCAAGGTT-AAACAGCCTGCAATCCTCGTCCACACCCCA 1083
NC_001414 GCGGGG-CCTAGTGGCCGCCCCGGTGGGACAAAAACTGCAGGCTTGTGCACA-TTGGGCCCCCAAGGTT-AAACAGCCTGCAATCCTCGTCCACACCCCA 1083
K02120 GCGAGG-CC-AGTGGCCGC---GGTGGGGCAAAAACTGCAGGCTTGCGCACAATTGGGCCCCCAAGAATGAAACAGCCTGCACTTCTCGTCCACACCCCA 1080
D00647 GCGGGG-CTTAGTGGCCGCCCCGGTGGGACAAAAACTGCAGGCTTGTGCACA-CTGGGCCCCCAAGACT-AAACAGCCTGCAATCCTCGTCCACACCCCA 1080
AF257515 GCGGGG-CCTAGTGGCCGCCCCGGTGGGGCAAAAACTGCAGGCTTGTGCACA-TTGGGCCCCCAAGATC-AAACAGCCTGCGATCCTCGTCCACACCCCA 1083
ruler ......1110......1120......1130......1140......1150......1160......1170......1180......1190......1200

```
                              *        *  * ***       *          **  ** *  ** *      *    ** ** ***    * ** **
    M10060 AGGTCCTTGTGGTCCAA-CCACGGAGGCCCCCCCCCACA----C---------AGCCC-TGCTTTCGTTGTGGCAAGGTAGGACACTGGAGTCGGGACTG- 1172
 NC_001488 AGGTCCTTGTGGTCCAA-CCACGGAGGCCCCCCCCCACA----C---------AGCCC-TGCTTTCGTTGTGGCAAGGTAGGACACTGGAGTCGGGACTG- 1172
  AF412314 AGGTCCTTGTGGTCCAA-CCACGGAGGCCCCCCCCCACA----C---------AGCCC-TGCTTTCGTTGTGGCAAGGTAGGACACTGGAGTCGGGACTG- 1172
  AF139382 AGGTCCTTGTGGTCCAA-CCACGGAAGCCCCCCCCCACA----C---------AGCCC-TGCTTTCGTTGTGGCAAGATAGGACACTGGAGTCGGGACTG- 1172
  AF326584 AGGTCCTTGTGGTCCAA-CCACGGAAGCCCCCCCCCACA----C---------AGCCC-TGCTTTCGTTGTGGCAAGATAGGACACTGGAGTCGGGACTG- 1172
  AF326583 AGGTCCTTGTGGTCCAA-CCACGGAAGCCCCCCCCCACA----C---------AGCCC-TGCTTTCGTTGTGGCAAGATAGGACACTGGAGTCGGGACTG- 1172
    L20734 AGGTCCTTGTGGTCCAA-CCACGGAGGCCCCCCCCCACA----C---------AGCCC-TGCTTTCGTTGTGGCAAGATAGGACACTGGAGTCGGGACTG- 1172
    Y13051 AGGTCCTTGTGGTCCAA-CCACGGAGGCCCCCCCCCACA----C---------AGCCC-TGCTTTCGTTGTGGCAAAATAGGACACTGGAGTCGGGACTG- 1172
    L11456 AGGTCCTTGTGGTCCAA-CCACGGAGGCCCCCCCCCACA----C---------AGCCC-TGCTTTCGTTGTGGCAAGGTAGGACACTGGAGTCGGGACTG- 1172
    X89270 AGGTCCTTGTGGTCCAA-CCACGGAGGCCCCCCCCCACA----C---------AGCCC-TGCTTTCGTTGTGGCAAGATAGGACACTGGAGTCGGGACTG- 1172
  AF074965 AGGTCCTTGTGGTCCAA-CCACGGAGGCCCCCCCCCACA----C---------AGCCC-TGCTTTCGTTGTGGCAAGGTAGGACACTGGAGTCGGGACTG- 1107
    Y14365 AGGTCCTTGTGGTCCAA-CCACGGAGGCCCCCCCCCACA----C---------AGCCC-TGCTTTCGTTTTGGCAAGGTAGGACACTGGGGTCGGGACTG- 1172
    Y14570 GGGTCCTCGTCGTCCAA-CCACGAAAGCCCCCGCCCACG----C---------AGCCC-TGCTTCCGCTGCGGGAAAACAGGGCATTGGAGCCGAGACTG- 1169
 NC_001815 GGGTCCTCGTCGTCCAA-CCACGAAAGCCCCCGCCCACG----C---------AGCCC-TGCTTCCGCTGCGGGAAAACAGGGCATTGGAGCCGAGACTG- 1169
    U90557 GGGTCCTCGTCGTCCAA-TCACGAAAGCCCCCGCCCACG----C---------AGCCCCTG-TTCCGCTGTGGAAAGGCAGGGCATTGGAGCCGAGACTG- 1168
    Y07616 AAATACTGATGATACAG-CCTAAAAAGACTCCTCCCCCGAAC-C---------AGCCG-TGCTTCCGCTGCGGGCAAGCGGGCCATTGGAGCAGGGACTG- 1155
  AY217650 AAATACTAATGATACAG-CCTAAAAAGACTCCTCCCCCGAAC-C---------AGCCA-TGCTTCCGCTGCGGGCAGGCAGGTCATTGGAGCAGAGACTG- 1152
  AF517775 AAATACTGATGATACAA-CCTAAAAAGACTCCTCCCCCAAAC-C---------AACCA-TGCTTCCGCTGCGGGCAAGTAGGTCATTGGAGCAGAGATTG- 1151
  AY222339 AAGTACTAATAATACAA-CCTAAAAAGACTCCTCCCCCAAAC-C---------AACCA-TGTTTCCGCTGCGGACAAGTAGGTCATTGGAGCAGAGATTG- 1151
 NC_003323 AAATACTAATGATACAA-CCTAAAAAGACTCCTCCCCCAAAC-C---------AACCA-TGCTTCCGCTGTGGGCAAGCAGGTCATTGGAGCAGAGATTG- 1152
    L36905 AAGTGTTAGTTGTCCAG-CCTAAAAAACCCCC-CCCAAAT---C---------AGCCG-TGCTTCCGGTGCGGGAAAGCAGGCCACTGGAGTCGGGACTG- 1157
  AF139170 AAGTGTTAGTTGTCCAG-CCTAAAAAACCCCC-CCCAAAT---C---------AGCCG-TGCTTCCGGTGCGGGAAAGCAGGCCACTGGAGTCGGGACTG- 1157
  AF042071 AAGTGTTAGTTGTCCAG-CCTAAAAAACCCCC-CCCAAAT---C---------AGCCG-TGCTTCCGGTGCGGGAAAGCAGGCCACTGGAGTCGAGACTG- 1157
    L03561 AAGTGTTAGTTGTCCAG-CCTAAAAAACCCCC-CCCAAAT---C---------AGCCG-TGCTTCCGGTGCGGGAAAGCAGGCCACTGGAGTCGGGACTG- 1157
  AF259264 AAGTGTTAGTTGTCCAG-CCTAAAAAACCCCC-CCCAAAT---C---------AGCCG-TGCTTCCGGTGCGGGAAAGCAGGCCACTGGAGTCGGGACTG- 1157
  AY563953 AAGTGTTAGTTGTCCAG-CCTAAAAAACCCCC-CCCAAAT---C---------AGCCG-TGCTTCCGGTGCGGGAAAGCAGGCCACTGGAGTCGGGACTG- 1157
  AY563954 AAGTGTTAGTTGTCCAG-CCTAAAAAACCCCC-CCCAAAT---C---------AGCCG-TGCTTCCGGTGCGGGAAAGCAGGCCACTGGAGTCGGGACTG- 1157
    U19949 AAGTGTTAGTTGTCCAG-CCTAAAAAACCCCC-CCCAAAT---C---------AGCCG-TGCTTCCGGTGCGGGAAAGCAGGCCACTGGAGTCGGGACTG- 1157
    J02029 AAGTGTTAGTTGTCCAG-CCTAAAAAACCCCC-CCCAAAT---C---------AGCCG-TGCTTCCGGTGCGGGAAAGCAGGCCACTGGAGTCGGGACTG- 1157
    D13748 AAGTGTTAGTTGTCCAG-CCTAAAAAACCCCC-CCCAAAT---C---------AGCCG-TGCTTCCGGTGCGGGAAAGCAGGCCACTGGAGTCGGGACTG- 1157
  AF033817 AAGTGTTAGTTGTCCAG-CCTAAAAAACCCCC-CCCAAAT---C---------AGCCG-TGCTTCCGGTGCGGGAAAGCAGGCCACTGGAGTCGGGACTG- 1157
 NC_001436 AAGTGTTAGTTGTCCAG-CCTAAAAAACCCCC-CCCAAAT---C---------AGCCG-TGCTTCCGGTGCGGGAAAGCAGGCCACTGGAGTCGGGACTG- 1157
  AF074966 AAGTGTTAGTTGTCCAG-CCTAAAAAACCCCC-CCCAAAT---C---------AGCCG-TGCTTCCGGTGCGGGAAAGCAGGTCACTGGAGCCGAGACTG- 1157
 NC_000858 AAGTGTTAGTTGTCCAG-CCTAAAAAACCCCC-CCCAAAT---C---------AGCCG-TGCTTCCGGTGCGGGAAAGCAGGTCACTGGAGCCGAGACTG- 1157
    L02534 AGGTGTTAGTCGTCCAG-CCTAAAAAACCCCC-CCCAAAT---C---------AGCCG-TGCTTCCGGTGCGGGAAAGCAGGCCACTGGAGCCGAGACTG- 1162
    Z46900 AAGTGCTAGTTATCCAG-CCCAAAAAACCCCC-CCCAAAT---C---------AGCCA-TGTTTCCGGTGCGGAAAAGCAGGCCACTGGAGCCGGGACTG- 1156
  AY590142 AGGTACTAGTAGTCCAG-CCAAAAAAGCCTCC-CCCAAAT---C---------AACCT-TGCTTCCGATGCGGGAAAGCTGGCCATTGGAGCCGAGACTG- 1199
  AF033818 GGGCCCAAGATGCCCGGGCCTCGGCAACCGGCCCCCAAAAGGCCCCCCCCGGGACCA-TGCTATCGATGCCTCAAAGAAGGCCATTGGGCCCGGGACTGT 1182
 NC_001414 GGGCCCAAGATGCCCGGGCCTCGGCAACCGGCCCCCAAAAGGCCCCCCCCGGGACCA-TGCTATCGATGCCTCAAAGAAGGCCATTGGGCCCGGGACTGT 1182
    K02120 GGGCCCAAGATGCCCGGGCCTCGGCAACCGGCCCCCAAAAGGCCTCCCCCAGGACCA-TGCTATCGATGCCTCAAAGAAGGCCATTGGGCCCGGGATTGT 1179
    D00647 GGGCCCAAGATGCCCGGGCCTCGGCAACCGGCCCCCAAAAGGCCCCCCCCGGGACCA-TGCTATCGATGCCTCAAAGAAGGCCATTGGGCCCGGGACTGT 1179
  AF257515 GGGCCCAAGATGCCTGGGCCCCGGCAACCGGCCCCCAAAAGGCCCCCCCCAGGACCA-TGCTATCGATGCCTCAAAGAAGGCCATTGGGCCCGGGACTGT 1182
     ruler ......1210......1220......1230......1240......1250......1260......1270......1280......1290......1300
```

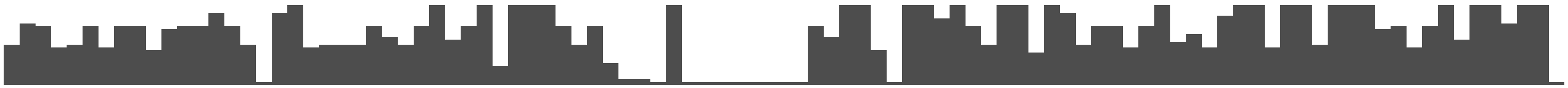

```
           ** *  * ** ** ** ** ** ** *   * **  * ** *   * ** *****  * ** ** **       **  *
M10060     --TACCCAG-CCAC--GCCCCCCTCCTGGCCCCTGCCCCCTATGCCAAGATCCTTCTCACTGGAAAAGGGACTGCCCACAACTCAAACCCC-CTCAGGA-  1265
NC_001488  --TACCCAG-CCAC--GCCCCCCTCCTGGCCCCTGCCCCCTATGCCAAGATCCTTCTCACTGGAAAAGGGACTGCCCACAACTCAAACCCC-CTCAGGA-  1265
AF412314   --TACCCAG-CCAC--GCCCCCCTCCTGGCCCCTGCCCCCTATGCCAAGATCCTTCTCACTGGAAAAGGGACTGCCCACAACTCAAACCCC-CTCAGGA-  1265
AF139382   --TACCCAG-CCAC--GCCCCCCTCCTGGCCCCTGCCCCCTATGCCAAGATCCTTCTCACTGGAAAAGGGACTGCCCACAACTCAAACCCC-CTCAGGA-  1265
AF326584   --TACCCAG-CCAC--GCCCCCCTCCTGGCCCCTGCCCCCTATGCCAAGAACCTTCTCACTGGAAAAGGGACTGCCCACAACTCAAACCCC-CTCAGGA-  1265
AF326583   --TACCCAG-CCAC--GCCCCCCTCCTGGCCCCTGCCCCCTATGCCAAGATCCTTCTCACTGGAAAAGGGACTGCCCACAACTCAAACCCC-CTCAGGA-  1265
L20734     --CACCCAG-CCAC--GCCCCCCTCCTGGCCCCTGCCCCCTATGCCAGGATCCTTCTCATTGGAAAAGGGACTGCCCACAGCTTAAACCCC-CTCAGGA-  1265
Y13051     --CACCCAG-CCAC--GCCCCCCTCCTGGCCCCTGCCCCCTATGCCAAGATCCTTCTCATTGGAAAAGGGACTGCCCACAGCTTAAACCCC-CTCAGGA-  1265
L11456     --CACCCAG-CCAC--GCCCCCCTCCTGGCCCCTGCCCCCTATGCCAGGATCCTTCTCACTGGAAAAGGGACTGCCCACAGCTTAAACCCC-CTCAGGA-  1265
X89270     --CACCCAG-CCAC--GCCCCCCTCCTGGCCCCTGCCCCCTATGCCAGGATCCTTCTCACTGGAAAAGGGACTGCCCACAACCCAAACCCC-CTCAGGA-  1265
AF074965   --CACCCAG-CCAC--GCCCCCCTCCTGGCCCCTGCCCCCTATGCCAGGATCCTTCTCACTGGAAAAGGGACTGCCCACAGCTTAAACCCC-CTCAGGA-  1200
Y14365     --CACCCAG-CCAC--GCCCCCCTCCTGGCCCCTGCCCCCTATGCCAAGATCCCTCTCACTGGAAAAGAGACTGCCCACAGCTTAAACCCC-CTCAGGA-  1265
Y14570     --CACCCTC-CCAC--GCCCCCCCCCTGGTCCGTGTCCCTTGTGCAAGGACCCTTCCCATTGGAAACGAGATTGTCCACAGTTCAAACCCC-CCCCAAC-  1262
NC_001815  --CACCCTC-CCAC--GCCCCCCCCCTGGTCCGTGTCCCTTGTGCAAGGACCCTTCCCATTGGAAACGAGATTGTCCACAGTTCAAACCCC-CCCCAAC-  1262
U90557     --CACCCTC-CCAC--GCCCCCCCCCTGGTCCATGCCCCTTGTGCAAAGACCCTTCCCATTGGAAACGAGATTGTCCACAGCTCAAACCCC-CCCCGAC-  1261
Y07616     --TAAACAG-CCCC--GGCCCCCTCCAGGCCCTTGCCCCCTATGCCAGGATCCCGCCCACTGGAAGCGGGACTGCCCACAGTTAAAAGCGGACACCAA--  1248
AY217650   --TAAACAG-CCCC--GGCCCCCTCCAGGCCCTTGCCCCCTATGCCAGGATCCCGCCCACTGGAAGCGGGACTGCCCACAGTTAAAAGCAGACACCAA--  1245
AF517775   --TAAACAG-CCTC--GGCCCCCTCCAGGCCCCTGCCCCGTATGTCAGGACCCCGCCCACTGGAAGCGGGACTGTCCACAATTAAAAACAGACACCAA--  1244
AY222339   --TAAACAG-CCTC--GGCCCCCTCCGGGCCCCTGCCCCGTATGTCAGGACCCCGCCCACTGGAAACGGGACTGCCCGCAGTTGAAAACAGATACCAA--  1244
NC_003323  --TAAACAG-CCCC--GACCCCCTCCGGGCCCCTGCCCTCTCTGCCAGGACCCCGCCCATTGGAAGCGGGACTGCCCACAGTTAAAAGCAGATACCAA--  1245
L36905     --CACTCAG-CCTC--GTCCCCCCCCCGGGCCATGCCCCCTATGTCAAGACCCAACTCACTGGAAGCGAGACTGCCCCCGCCTAAAGCCCACTATCCCAG  1252
AF139170   --CACTCAG-CCTC--GTCCCCCCCCCGGGCCATGCCCCCTATGTCAAGACCCAACTCACTGGAAGCGAGACTGCCCCCGCCTAAAGCCCACTATCCCAG  1252
AF042071   --CACTCAG-CCTC--GTCCCCCCCCCGGGCCATGCCCCCTATGTCAAGACCCAACTCACTGGAAGCGAGACTGCCCCCGCCTAAAGCCCACTATCCCAG  1252
L03561     --CACTCAG-CCCC--GTCCCCCCCCCGGGCCATGCCCCCTATGTCAAGACCCAACTCACTGGAAGCGAGACTGCCCCCGCCTAAAGCCCACTATCCCAG  1252
AF259264   --CACTCAG-CCTC--GCCCCCCCCCCGGGCCATGCCCCCTATGTCAAGACCCAACTCACTGGAAGCGAGACTGCCCCCGCCTAAAGCCCACTATCCCAG  1252
AY563953   --CACTCAG-CCTC--GTCCCCCCCCCGGGCCATGCCCCCTATGTCAAGACCCAACTCACTGGAAGCGAGACTGCCCCCGCCTAAAGCCCACTATCCCAG  1252
AY563954   --CACTCAG-CCTC--GTCCCCCCCCCGGGCCATGCCCCCTATGTCAAGACCCAACTCACTGGAAGCGAGACTGCCCCCGCCTAAAGCCCACTATCCCAG  1252
U19949     --CACTCAG-CCTC--GTCCCCCCCCCGGGCCATGCCCCCTATGTCAAGACCCAACTCACTGGAAGCGAGACTGCCCCCGCCTAAAGCCCACTATCCCAG  1252
J02029     --CACTCAG-CCTC--GTCCCCCCCCCGGGCCATGCCCCCTATGTCAAGACCCAACTCACTGGAAGCGAGACTGCCCCCGCCTAAAGCCCACTATCCCAG  1252
D13748     --CACTCAG-CCTC--GTCCTCCCCCTGGGCCATGCCCCCTATGTCAAGATCCAACTCACTGGAAGCGAGACTGCCCCCGCCTAAAGCCCACTATCCCAG  1252
AF033817   --CACTCAG-CCTC--GTCCTCCCCCTGGGCCATGCCCCCTATGTCAAGATCCAACTCACTGGAAGCGAGACTGCCCCCGCCTAAAGCCCACTATCCCAG  1252
NC_001436  --CACTCAG-CCTC--GTCCTCCCCCTGGGCCATGCCCCCTATGTCAAGATCCAACTCACTGGAAGCGAGACTGCCCCCGCCTAAAGCCCACTATCCCAG  1252
AF074966   --CACTCAG-CCTC--GTCCTCCCCCCGG-CCATGCC--CTATGTCAAGACCCAACTCACTGGAAGCGAGACTGCCCTCGCCTGAAGCCCGCTATCTCGG  1249
NC_000858  --CACTCAG-CCTC--GTCCTCCCCCCGG-CCATGCC--CTATGTCAAGACCCAACTCACTGGAAGCGAGACTGCCCTCGCCTGAAGCCCGCTATCTCGG  1249
L02534     --CGCTCAG-CCTC--GTCCTCCCCCCGGGCCATGCCCCCTCTGTCAAGACCCAACTCACTGGAAGCGGGACTGTCCCCGCCTAAAGCCCGCCATCCCAG  1257
Z46900     --CACTCAA-CCTC--GTCCTCCCCCCGGGCCATGCCCCCTCTGTCAGGACCCAACTCACTGGAAGCGGGACTGTCCTCGCCTAAAGCCCACCACCCCAG  1251
AY590142   --CACCCAG-CCTC--GCCCACCTCCCGGCCCATGCCCCCTTTGTCAAGACCCAACCCACTGGAAAAGGGACTGCCCTCGCCTAAAACCCGCCACCGC--  1292
AF033818   CCCACCAAGACCACCGGCCCCCCTCCGGGACCTTGTCCCATATGTAAAGATCCTTCCCATTGGAAACGAGACTGTCCAACCCTCAAATCAAAAAACTAA-  1281
NC_001414  CCCACCAAGACCACCGGCCCCCCTCCGGGACCTTGTCCCATATGTAAAGATCCTTCCCATTGGAAACGAGACTGTCCAACCCTCAAATCAAAAAACTAA-  1281
K02120     CCTACCAAGGCCACCGGCCCACCTCCGGGACCTTGCCCCATATGTAAAGATCCTTCCCATTGGAAACGAGACTGTCCAACCCTCAAATCAAAAAACTAA-  1278
D00647     CCCACCAAAACCACCGGCCCCCCTCCGGGACCTTGTCCCATATGTAAAGATCCTTCCCATTGGAAACGAGACTGTCCAACCCTCAAATCAAAAAACTAA-  1278
AF257515   CCCACCAAGACCACCGGCCCCCCTCCGGGACCTTGTCCTATATGTAAAGATCTTTCCCATTGGAAACGAGACTGTCCAACCCTCAAATCAAAAAACTAA-  1281
ruler      ......1310......1320......1330......1340......1350......1360......1370......1380......1390......1400
```

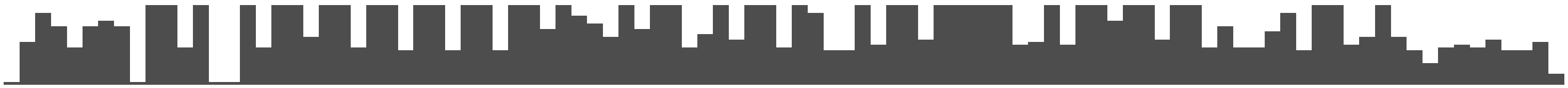

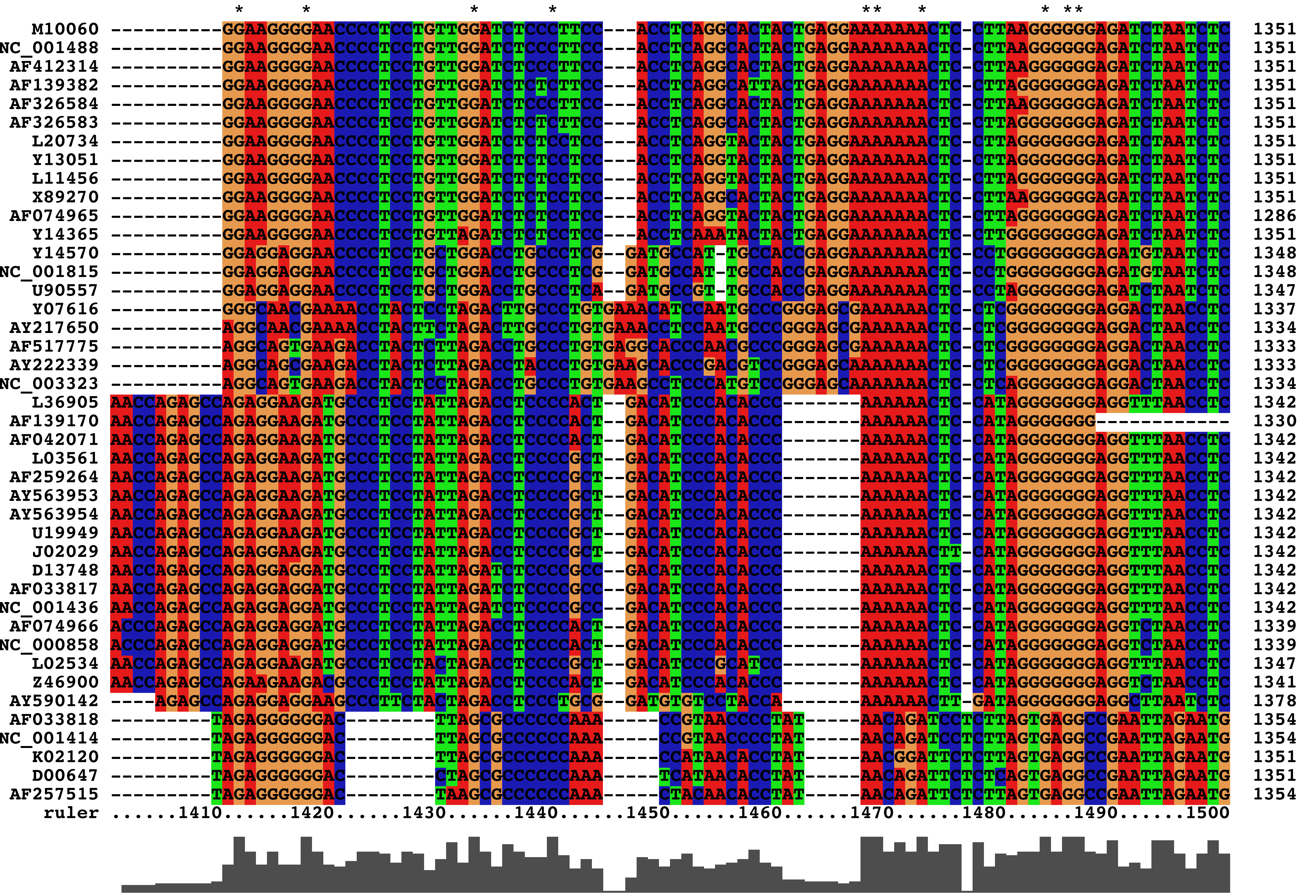
M10060 1351
NC_001488 1351
AF412314 1351
AF139382 1351
AF326584 1351
AF326583 1351
L20734 1351
Y13051 1351
L11456 1351
X89270 1351
AF074965 1286
Y14365 1351
Y14570 1348
NC_001815 1348
U90557 1347
Y07616 1337
AY217650 1334
AF517775 1333
AY222339 1333
NC_003323 1334
L36905 1342
AF139170 1330
AF042071 1342
L03561 1342
AF259264 1342
AY563953 1342
AY563954 1342
U19949 1342
J02029 1342
D13748 1342
AF033817 1342
NC_001436 1342
AF074966 1339
NC_000858 1339
L02534 1347
Z46900 1341
AY590142 1378
AF033818 1354
NC_001414 1354
K02120 1351
D00647 1351
AF257515 1354
ruler ......1410......1420......1430......1440......1450......1460......1470......1480......1490......1500

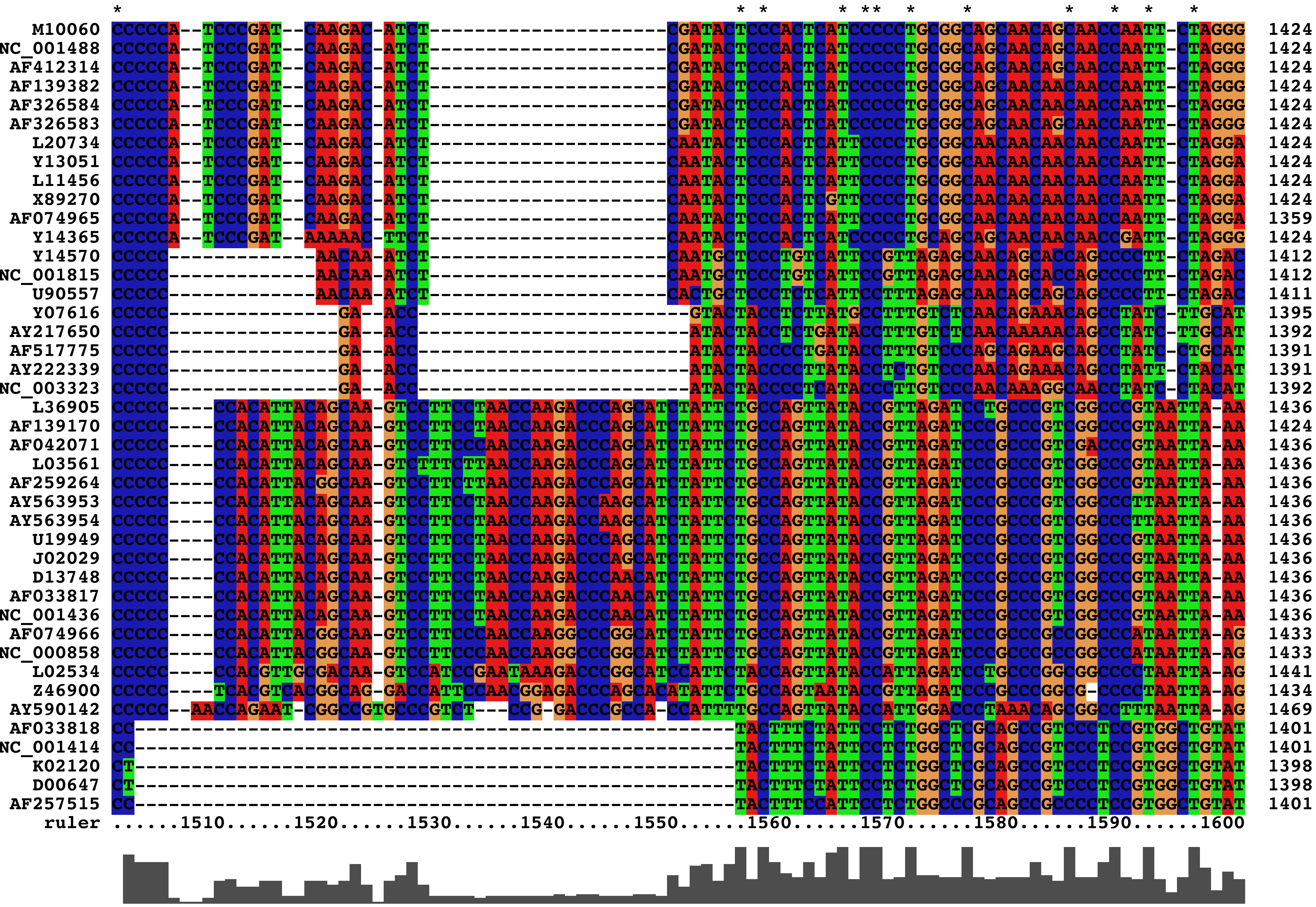
M10060 CCCCCA--TCCCGAT--CAAGAC-ATCT---------------------CGATACTCCCACTCATCCCCCTGCGGCAGCAACAGCAACCAATT-CTAGGG 1424
NC_001488 CCCCCA--TCCCGAT--CAAGAC-ATCT---------------------CGATACTCCCACTCATCCCCCTGCGGCAGCAACAGCAACCAATT-CTAGGG 1424
AF412314 CCCCCA--TCCCGAT--CAAGAC-ATCT---------------------CGATACTCCCACTCATCCCCCTGCGGCAGCAACAGCAACCAATT-CTAGGG 1424
AF139382 CCCCCA--TCCCGAT--CAAGAC-ATCT---------------------CGATACTCCCACTCATCCCCCTGCGGCAGCAACAACAACCAATT-CTAGGG 1424
AF326584 CCCCCA--TCCCGAT--CAAGAC-ATCT---------------------CGATACTCCCACTCATCCCCCTGCGGCAGCAACAACAACCAATT-CTAGGG 1424
AF326583 CCCCCA--TCCCGAT--CAAGAC-ATCT---------------------CGATACTCCCACTCATCCCCCTGCGGCAGCAACAGCAACCAATT-CTAGGG 1424
L20734 CCCCCA--TCCCGAT--CAAGAC-ATCT---------------------CAATACTCCCACTCATTCCCCTGCGGCAACAACAACAACCAATT-CTAGGA 1424
Y13051 CCCCCA--TCCCGAT--CAAGAC-ATCT---------------------CAATACTCCCACTCATTCCCCTGCGGCAACAACAACAACCAATT-CTAGGA 1424
L11456 CCCCCA--TCCCGAT--CAAGAC-ATCT---------------------CAATACTCCCACTCATTCCCCTGCGGCAACAACAACAACCAATT-CTAGGA 1424
X89270 CCCCCA--TCCCGAT--CAAGAC-ATCT---------------------CAATACTCCCACTCGTTCCCCTGCGGCAACAACAACAACCAATT-CTAGGA 1424
AF074965 CCCCCA--TCCCGAT--CAAGAC-ATCT---------------------CAATACTCCCACTCATTCCCCTGCGGCAACAACAACAACCAATT-CTAGGA 1359
Y14365 CCCCCA--TCCCGAT--AAAAAC-TTCT---------------------CAATACTCCCACTCATCCCCCTGCAGCAGCAACAACAACCGATT-CTAGGG 1424
Y14570 CCCCC-------------AACAA-ATCT---------------------CAATGCTCCCTGTCATTCCGTTAGAGCAACAGCACCAGCCCCTT-CTAGAC 1412
NC_001815 CCCCC-------------AACAA-ATCT---------------------CAATGCTCCCTGTCATTCCGTTAGAGCAACAGCACCAGCCCCTT-CTAGAC 1412
U90557 CCCCC-------------AACAA-ATCT---------------------CACTGCTCCCTGTCATTCCTTTAGAGCAACAGCAGCAGCCCCTT-CTAGAC 1411
Y07616 CCCCC---------------GA--ACC------------------------GTACTACCTCTTATGCCTTTGTCTCAACAGAAACAGCCTATC-TTGCAT 1395
AY217650 CCCCC---------------GA--ACC------------------------ATACTACCTCTGATACCTTTGTCTCAACAAAAACAGCCTATC-TTGCAT 1392
AF517775 CCCCC---------------GA--ACC------------------------ATACTACCCCTGATACCTTTGTCCCAGCAGAAGCAGCCTATC-CTGCAT 1391
AY222339 CCCCC---------------GA--ACC------------------------ATACTACCCCTTATACCTCTGTCCCAACAGAAACAGCCTATT-CTACAT 1391
NC_003323 CCCCC---------------GA--ACC------------------------ATACTACCCCTCATACCCTTGTCCCAACAAAGGCAACCTATC-CTACAT 1392
L36905 CCCCC----CCACATTACAGCAA-GTCCTTCCTAACCAAGACCCAGCATCTATTCTGCCAGTTATACCGTTAGATCCTGCCCGTCGGCCCGTAATTA-AA 1436
AF139170 CCCCC----CCACATTACAGCAA-GTCCTTCCTAACCAAGACCCAGCATCTATTCTGCCAGTTATACCGTTAGATCCCGCCCGTCGGCCCGTAATTA-AA 1424
AF042071 CCCCC----CCACATTACAGCAA-GTCCTTCCCAACCAAGACCCAGCATCTATTCTGCCAGTTATACCGTTAGATCCCGCCCGTCGACCCGTAATTA-AA 1436
L03561 CCCCC----CCACATTACAGCAA-GTCTTTCTTAACCAAGACCCAGCATCTATTCTGCCAGTTATACCGTTAGATCCCGCCCGTCGGCCCGTAATTA-AA 1436
AF259264 CCCCC----CCACATTACGGCAA-GTCCTTCTTAACCAAGACCCAGCATCTATTCTGCCAGTTATACCGTTAGATCCCGCCCGTCGGCCCGTAATTA-AA 1436
AY563953 CCCCC----CCACATTACAGCAA-GTCCTTCCTAACCAAGACCAAGCATCTATTCTGCCAGTTATACCGTTAGATCCCGCCCGTCGGCCCTTAATTA-AA 1436
AY563954 CCCCC----CCACATTACAGCAA-GTCCTTCCTAACCAAGACCAAGCATCTATTCTGCCAGTTATACCGTTAGATCCCGCCCGTCGGCCCTTAATTA-AA 1436
U19949 CCCCC----CCACATTACAGCAA-GTCCTTCCTAACCAAGACCCAGCATCTATTCTGCCAGTTATACCGTTAGATCCCGCCCGTCGGCCCGTAATTA-AA 1436
J02029 CCCCC----CCACATTACAGCAA-GTCCTTCCTAACCAAGACCCAGCATCTATTCTGCCAGTTATACCGTTAGATCCCGCCCGTCGGCCCGTAATTA-AA 1436
D13748 CCCCC----CCACATTACAGCAA-GTCCTTCCTAACCAAGACCCAACATCTATTCTGCCAGTTATACCGTTAGATCCCGCCCGTCGGCCCGTAATTA-AA 1436
AF033817 CCCCC----CCACATTACAGCAA-GTCCTTCCTAACCAAGACCCAACATCTATTCTGCCAGTTATACCGTTAGATCCCGCCCGTCGGCCCGTAATTA-AA 1436
NC_001436 CCCCC----CCACATTACAGCAA-GTCCTTCCTAACCAAGACCCAACATCTATTCTGCCAGTTATACCGTTAGATCCCGCCCGTCGGCCCGTAATTA-AA 1436
AF074966 CCCCC----CCACATTACGGCAA-GTCCTTCCCAACCAAGGCCCGGCATCTATTCTGCCAGTTATACCGTTAGATCCCGCCCGCCGGCCCATAATTA-AG 1433
NC_000858 CCCCC----CCACATTACGGCAA-GTCCTTCCCAACCAAGGCCCGGCATCTATTCTGCCAGTTATACCGTTAGATCCCGCCCGCCGGCCCATAATTA-AG 1433
L02534 CCCCC----CCACGTTGCGACAA-GTCCATCCGAATAAAGACCCGGCATCCATTCTACCAGTTATACCATTAGATCCTGCCCGGCGGCCCCTAATTA-AG 1441
Z46900 CCCCC----TCACGTCACGGCAG-GACCATTCCAACGGAGACCCAGCACATATTCTGCCAGTAATACCGTTAGATCCCGCCCGGCG-CCCCTAATTA-AG 1434
AY590142 CCCCC--AACCAGAAT-CGGCCGTGCCCGTCT---CCG-GACCCGCCA-CCATTTTGCCAGTTATACCATTGGACCCTAAACAGCGGCCTTTAATTA-AG 1469
AF033818 CC-----------------------------------------------------TACTTTCTATTCCTCTGGCTCGCAGCCGTCCCTCCGTGGCTGTAT 1401
NC_001414 CC-----------------------------------------------------TACTTTCTATTCCTCTGGCTCGCAGCCGTCCCTCCGTGGCTGTAT 1401
K02120 CT-----------------------------------------------------TACTTTCTATTCCTCTGGCTCGCAGCCGTCCCTCCGTGGCTGTAT 1398
D00647 CT-----------------------------------------------------TACTTTCTATTCCTCTGGCTCGCAGCCGTCCCTCCGTGGCTGTAT 1398
AF257515 CC-----------------------------------------------------TACTTTCCATTCCTCTGGCCCGCAGCCGCCCCTCCGTGGCTGTAT 1401
ruler ......1510......1520......1530......1540......1550......1560......1570......1580......1590......1600

```
                  *                   *               * **  *     *    ** ** ** ** **    ** **  *   **
M10060    GTCCGGATCTCCGTTATGGG--ACAAACACC-TCAGCCTACCCAAGCGCT---ACTT---GACACAGGAGCCGACCTTACGGTTATACCCCAGAC-ACTC 1514
NC_001488 GTCCGGATCTCCGTTATGGG--ACAAACACC-TCAGCCTACCCAAGCGCT---ACTT---GACACAGGAGCCGACCTTACGGTTATACCCCAGAC-ACTC 1514
AF412314  GTCCGGATCTCCGTTATGGG--ACAAACACC-TCAGCCTACCCAAGCGCT---ACTT---GACACAGGAGCCGACCTTACGGTTATACCCCAGAC-ACTC 1514
AF139382  GTCCGGATCTCCGTTATGGG--ACAAACACC-TCAGCCTACCCAAGCGCT---ACTT---GACACAGGAGCCGACCTTACGGTTATACCCCAGAC-ACTC 1514
AF326584  GTCCGGATCTCCGTTATGGG--ACAAACACC-TCAGCCTACCCAAGCGCT---ACTT---GACACAGGAGCCGACCTTACGGTTATACCCCAGAC-ACTC 1514
AF326583  GTCCGGATATCCGTTATGGG--ACAAACACC-TCAGCCTACCCAAGCGCT---ACTT---GACACAGGAGCCGACCTTACGGTTATACCCCAGAC-ACTC 1514
L20734    GTCCGGATTTCCGTTATGGG--ACAAACACC-TCAGCCTACCCAAGCGCT---ACTT---GACACAGGAGCCGACCGTACGGTTATACCCCAGAC-ACTC 1514
Y13051    GTCCGGATTTCCGTTATGGG--GCAAACACC-TCAGCCTACCCAAGCGCT---ACTT---GACACAGGAGCCGACCGTACGGTTATACCCCAGGC-ACTC 1514
L11456    GTCCGGATTTCCGTTATGGG--ACAAACACC-TCAGCCTACCCAAGCGCT---ACTT---GACACAGGAGCCGACCGTACGGTTATACCCCAGAC-ACTC 1514
X89270    GTCCGGATTTCCGTTATGGG--GCAAACACC-TCAGCCTACCCAAGCGCT---ACTT---GACACAGGAGCCGACCGTACGGTTATACCCCAGAC-ACTC 1514
AF074965  GTCCGGATTTCCGTTATGGG--GCAAACACC-TCAGCCTACCCAAGCGCT---ACTT---GACACAGGAGCCGACCGTACGGTTATACCCCATAC-ACTC 1449
Y14365    GTCCGGATTTCCGTTATGGG--ACAAACACC-TCAGCCTACCCAAGCGCT---ACTG---GACACAGGAGCCGACCTTACGGTCATACCCCAGAC-ACTC 1514
Y14570    GTTCAAGTCTCCATTGCAGGC-GCCCCC-CC-TCGACCTACCCAGGCACT----CTTA--GACACGGGGGCTGATCTCACCGTCCTGCCCCAGGC-CCTT 1502
NC_001815 GTTCAAGTCTCCATTGCAGGC-GCCCCC-CC-TCGACCTACCCAGGCACT----CTTA--GACACGGGGGCTGATCTCACCGTCCTGCCCCAGGC-CCTT 1502
U90557    GTTCAGGTCTCCATTGCAGGC-GCCCCC-CC-TCGACCTACCCAGGCACT----CTTA--GACACAGGGGCTGATCTCACCGTCCTGCCCCAGGC-CCTT 1501
Y07616    GTTCAGGTGTCCCTCTCCAAT-ACCTCCCCTGTGGAC--ATCCAAGCCCT---CCTT---GACACCGGAGCGGACGTTACAGTCCT--CCCGGCTTACTT 1484
AY217650  GTTCAGGTGTCCCTCTCCAAT-ACCTCCCCTGTGGAC--ATCCAAGCCCT---CCTT---GACACCGGGGCGGACGTTACAGTCCT--CCCGGCCTACTT 1481
AF517775  GTCCAGGTGTCCTTCTCCAAC-ACCTCCCCTGTTAGC--ATTCAAGCACT---CCTC---GATACCGGGGCAGACATCACTGTCCT--CCCGGCCTGCTT 1480
AY222339  GTCCAGGTGTCCTTTTCCAAC-ACCTCCCCGGTCAAC--GTTCAAGCGCT---ACTC---GACACCGGGGCAGACATCACTGTCCT--CCCTGCCTGCTT 1480
NC_003323 GTCCAGGTGTCCTTTTCCAAC-ACCTCCCCTGTTGGC--GTTCAAGCACT---CCTC---GACACCGGGGCGGACATTACTGTTCT--CCCGGCCTACCT 1481
L36905    GCCCAGGTTGACACC-CAGACCAGCCAC-CCAAAGAC-TATCGAAGCTTT---ACTA---GATACAGGAGCAGACATGACAGTCCT--TCCGATAGCCTT 1525
AF139170  GCCCAGGTTGACACC-CAGACCAGCCAC-CCAAAGAC-TATCGAAGCTTT---ACTA---GATACAGGAGCAGACATGACAGTCCT--TCCGATAGCCTT 1513
AF042071  GCCCAGGTTGACACC-CAGACCAGCCAC-CCAAAGAC-TATCGAAGCTTT---ACTA---GATACGGGAGCAGACATGACAGTCCT--TCCGATAGCCTT 1525
L03561    GCCCAGGTTGACACC-CAGACCAGCCAC-CCAAAGAC-TATCGAAGCTCT---ACTA---GATACAGGAGCAGACATGACAGTCCT--TCCGATAGCCTT 1525
AF259264  GCCCAGGTTGACACC-CAGACCAGCCAC-CCAAAGAC-TATCGAAGCTTT---ACTA---GATACAGGAGCAGACATGACAGTCCT--TCCGATAGCCTT 1525
AY563953  GCCCAGGTTGACACC-CAGACCAGCCAC-CCAAAGAC-TATCGAAGCTTT---ACTA---GATACAGGAGCAGACATGACAGTCCT--TCCGATAGCCTT 1525
AY563954  GCCCAGGTTGACACC-CAGACCAGCCAC-CCAAAGAC-TATCGAAGCTTT---ACTA---GATACAGGAGCAGACATGACAGTCCT--TCCGATAGCCTT 1525
U19949    GCCCAGGTTGACACC-CAGACCAGCCAC-CCAAAGAC-TATCGAAGCTTT---ACTA---GATACAGGAGCAGACATGACAGTCCT--TCCGATAGCCTT 1525
J02029    GCCCAGGTTGACACC-CAGACCAGCCAC-CCAAAGAC-TATCGAAGCTTT---ACTA---GATACAGGAGCAGACATGACAGTCCT--TCCGATAGCCTT 1525
D13748    GCCCAGATTGACACC-CAGACCAGCCAC-CCAAAGAC-TATCGAAGCTCT---ACTA---GATACAGGAGCAGACATGACAGTCCT--TCCGATAGCCTT 1525
AF033817  GCCCAGATTGACACC-CAGACCAGCCAC-CCAAAGAC-TATCGAAGCTCT---ACTA---GATACAGGAGCAGACATGACAGTCCT--TCCGATAGCCTT 1525
NC_001436 GCCCAGATTGACACC-CAGACCAGCCAC-CCAAAGAC-TATCGAAGCTCT---ACTA---GATACAGGAGCAGACATGACAGTCCT--TCCGATAGCCTT 1525
AF074966  GCCCAGGTTGACACC-CAGACCAGCCAC-CCAAAGAC-TATCGAAGCTTT---ACTA---GATACAGGAGCAGACATGACAGTCCT--TCCAATAGCCTT 1522
NC_000858 GCCCAGGTTGACACC-CAGACCAGCCAC-CCAAAGAC-TATCGAAGCTTT---ACTA---GATACAGGAGCAGACATGACAGTCCT--TCCAATAGCCTT 1522
L02534    GCCCAAGTTGACACC-CAGACCAGTCAC-CCGAGGAC-CATCGAAGCCTT---ATTA---GATACAGGAGCAGACATGACAGTCCT--TCCAATAGCCTT 1530
Z46900    GCCCAAGTGGACACC-CAGACCAATGGC-CCAAAAAC-CATCGAAGCGTT---ACTA---GATACGGGGGCGGACATGACGGTCCT--TCCAATAGCCCT 1523
AY590142  GCCGAAATTGAAGTC-CCAACAAATT-C-CCGAAAACCTGTTCAAGCCCT---GTTA---GACACTGGGGCGGACATGACCGTCCT--CCCCCTTGCCCT 1558
AF033818  ACCTGTCTGGCCCCT---GGCTGCAGCCCTCTCAGAA----TCAAGCCCTTATGCTCGTGGACACCGGGGCTGAAAATACGGTCCT--CCCACAAAATTG 1492
NC_001414 ACCTGTCTGGCCCCT---GGCTGCAGCCCTCTCAGAA----TCAAGCCCTTATGCTCGTGGACACCGGGGCTGAAAATACGGTCCT--CCCACAAAATTG 1492
K02120    ACCTGTCTGGCCCCT---GGCTGCAGCCCTCTCAGAA----TCAAGCCCTCATGCTTGTGGACACCGGGGCTGAAAATACGGTTCT--CCCACAAAATTG 1489
D00647    ACCTGTCTGGCCCCT---GGCTGCAGCCCTCTCAAAA----TCAAGCCCTCATGCTCGTGGACACCGGGGCTGAAAATACGGTCCT--CCCACAAAATTG 1489
AF257515  ACCTGTCTGGGCCCT---GGCTGCAGCCCTCTCAAAA----TCAAGCCCTCATGCTCGTGGACACCGGGGCTGAAAATACAGTTCT--CCCACAAAATTG 1492
ruler     ......1610......1620......1630......1640......1650......1660......1670......1680......1690......1700
```

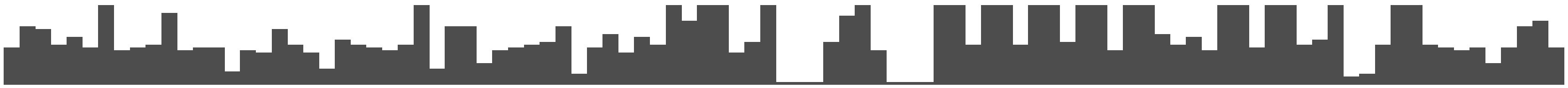

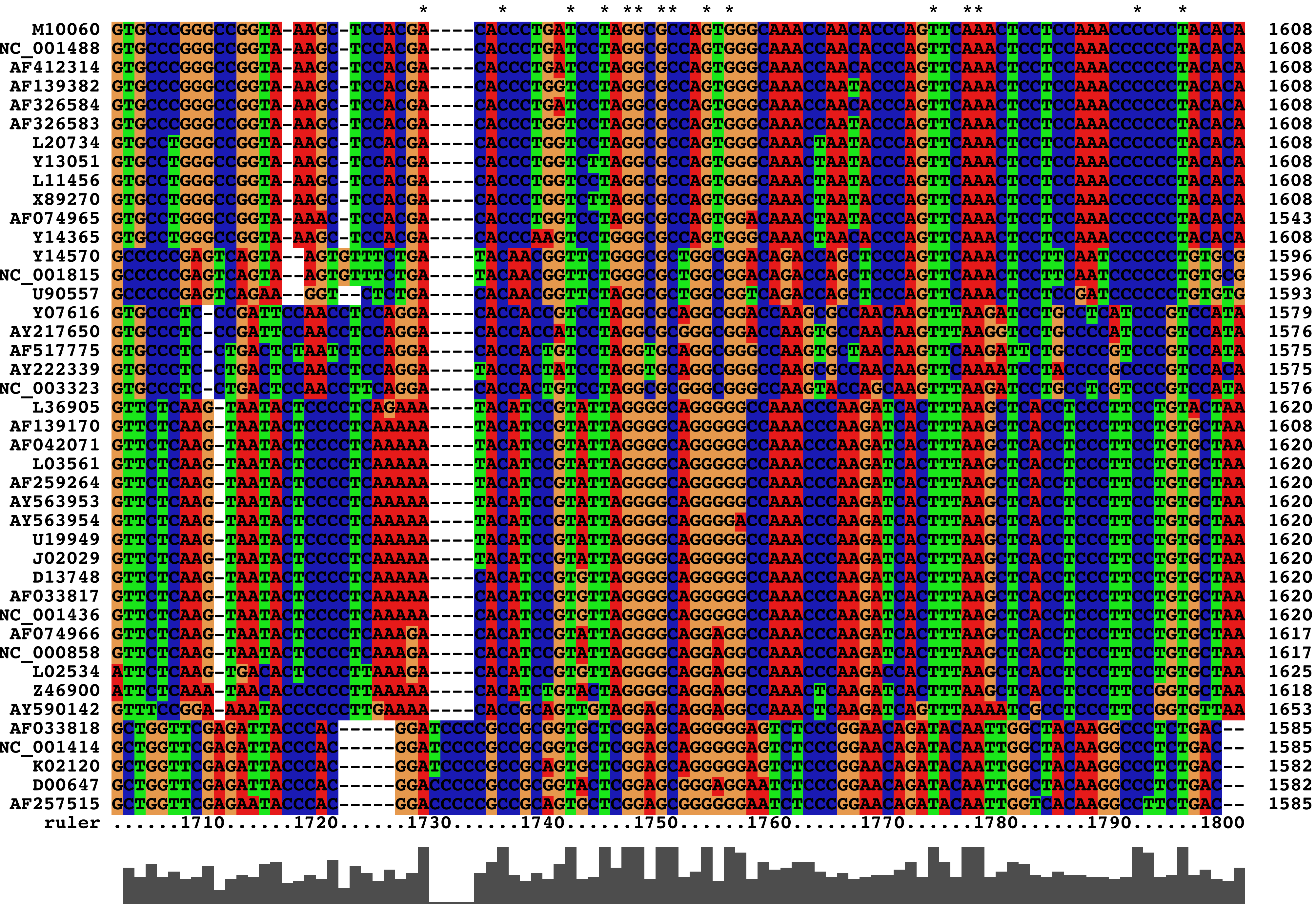
* * * * ** ** * * * ** * *
M10060 GTGCCCGGGCCGGTA-AAGC-TCCACGA----CACCCTGATCCTAGGCGCCAGTGGGCAAACCAACACCCAGTTCAAACTCCTCCAAACCCCCCTACACA 1608
NC_001488 GTGCCCGGGCCGGTA-AAGC-TCCACGA----CACCCTGATCCTAGGCGCCAGTGGGCAAACCAACACCCAGTTCAAACTCCTCCAAACCCCCCTACACA 1608
AF412314 GTGCCCGGGCCGGTA-AAGC-TCCACGA----CACCCTGATCCTAGGCGCCAGTGGGCAAACCAACACCCAGTTCAAACTCCTCCAAACCCCCCTACACA 1608
AF139382 GTGCCCGGGCCGGTA-AAGC-TCCACGA----CACCCTGGTCCTAGGCGCCAGTGGGCAAACCAATACCCAGTTCAAACTCCTCCAAACCCCCCTACACA 1608
AF326584 GTGCCCGGGCCGGTA-AAGC-TCCACGA----CACCCTGATCCTAGGCGCCAGTGGGCAAACCAACACCCAGTTCAAACTCCTCCAAACCCCCCTACACA 1608
AF326583 GTGCCCGGGCCGGTA-AAGC-TCCACGA----CACCCTGGTCCTAGGCGCCAGTGGGCAAACCAATACCCAGTTCAAACTCCTCCAAACCCCCCTACACA 1608
L20734 GTGCCTGGGCCGGTA-AAGC-TCCACGA----CACCCTGGTCCTAGGCGCCAGTGGGCAAACTAATACCCAGTTCAAACTCCTCCAAACCCCCCTACACA 1608
Y13051 GTGCCTGGGCCGGTA-AAGC-TCCACGA----CACCCTGGTCTTAGGCGCCAGTGGGCAAACTAATACCCAGTTCAAACTCCTCCAAACCCCCCTACACA 1608
L11456 GTGCCTGGGCCGGTA-AAGC-TCCACGA----CACCCTGGTCCTAGGCGCCAGTGGGCAAACTAATACCCAGTTCAAACTCCTCCAAACCCCCCTACACA 1608
X89270 GTGCCTGGGCCGGTA-AAGC-TCCACGA----CACCCTGGTCTTAGGCGCCAGTGGGCAAACTAATACCCAGTTCAAACTCCTCCAAACCCCCCTACACA 1608
AF074965 GTGCCTGGGCCGGTA-AAAC-TCCACGA----CACCCTGGTCCTAGGCGCCAGTGGACAAACTAATACCCAGTTCAAACTCCTCCAAACCCCCCTACACA 1543
Y14365 GTGCCTGGGCCGGTA-AAGC-TCCACGA----CACCCAAGTCCTGGGCGCCAGTGGGCAAACTAACACCCAGTTCAAACTCCTCCAAACCCCCCTACACA 1608
Y14570 GCCCCCGAGTCAGTA--AGTGTTTCTGA----TACAACGGTTCTGGGCGCTGGCGGACAGACCAGCTCCCAGTTCAAACTCCTTCAATCCCCCCTGTGCG 1596
NC_001815 GCCCCCGAGTCAGTA--AGTGTTTCTGA----TACAACGGTTCTGGGCGCTGGCGGACAGACCAGCTCCCAGTTCAAACTCCTTCAATCCCCCCTGTGCG 1596
U90557 GCCCCCGAGTCAGAA--GGT--CTCTGA----CACAACGGTTCTAGGCGCTGGCGGTCAGACCAGCTCCCAGTTCAAACTCCTCCGATCCCCCCTGTGTG 1593
Y07616 GTGCCCTC-CCGATTCCAACCTCCAGGA----CACCACCGTCCTAGGCGCAGGCGGACCAAGCGCCAACAAGTTTAAGATCCTGCCTCATCCCGTCCATA 1579
AY217650 GTGCCCTC-CCGATTCCAACCTCCAGGA----CACCACCATCCTAGGCGCGGGCGGACCAAGTGCCAACAAGTTTAAGGTCCTGCCCCATCCCGTCCATA 1576
AF517775 GTGCCCTC-CTGACTCTAATCTCCAGGA----CACCACTGTCCTAGGTGCAGGCGGGCCAAGTGCTAACAAGTTCAAGATTCTGCCCCGTCCCGTCCATA 1575
AY222339 GTGCCCTC-CTGACTCCAACCTCCAGGA----TACCACTATCCTAGGTGCAGGCGGGCCAAGCGCCAACAAGTTCAAAATCCTACCCCGCCCCGTCCACA 1575
NC_003323 GTGCCCTC-CTGACTCCAACCTTCAGGA----CACCACTGTCCTAGGCGCGGGCGGGCCAAGTACCAGCAAGTTTAAGATCCTGCCTCGTCCCGTCCATA 1576
L36905 GTTCTCAAG-TAATACTCCCCTCAGAAA----TACATCCGTATTAGGGGCAGGGGGCCAAACCCAAGATCACTTTAAGCTCACCTCCCTTCCTGTACTAA 1620
AF139170 GTTCTCAAG-TAATACTCCCCTCAAAAA----TACATCCGTATTAGGGGCAGGGGGCCAAACCCAAGATCACTTTAAGCTCACCTCCCTTCCTGTGCTAA 1608
AF042071 GTTCTCAAG-TAATACTCCCCTCAAAAA----TACATCCGTATTAGGGGCAGGGGGCCAAACCCAAGATCACTTTAAGCTCACCTCCCTTCCTGTGCTAA 1620
L03561 GTTCTCAAG-TAATACTCCCCTCAAAAA----TACATCCGTATTAGGGGCAGGGGGCCAAACCCAAGATCACTTTAAGCTCACCTCCCTTCCTGTGCTAA 1620
AF259264 GTTCTCAAG-TAATACTCCCCTCAAAAA----TACATCCGTATTAGGGGCAGGGGGCCAAACCCAAGATCACTTTAAGCTCACCTCCCTTCCTGTGCTAA 1620
AY563953 GTTCTCAAG-TAATACTCCCCTCAAAAA----TACATCCGTATTAGGGGCAGGGGGCCAAACCCAAGATCACTTTAAGCTCACCTCCCTTCCTGTGCTAA 1620
AY563954 GTTCTCAAG-TAATACTCCCCTCAAAAA----TACATCCGTATTAGGGGCAGGGGACCAAACCCAAGATCACTTTAAGCTCACCTCCCTTCCTGTGCTAA 1620
U19949 GTTCTCAAG-TAATACTCCCCTCAAAAA----TACATCCGTATTAGGGGCAGGGGGCCAAACCCAAGATCACTTTAAGCTCACCTCCCTTCCTGTGCTAA 1620
J02029 GTTCTCAAG-TAATACTCCCCTCAAAAA----TACATCCGTATTAGGGGCAGGGGGCCAAACCCAAGATCACTTTAAGCTCACCTCCCTTCCTGTGCTAA 1620
D13748 GTTCTCAAG-TAATACTCCCCTCAAAAA----CACATCCGTGTTAGGGGCAGGGGGCCAAACCCAAGATCACTTTAAGCTCACCTCCCTTCCTGTGCTAA 1620
AF033817 GTTCTCAAG-TAATACTCCCCTCAAAAA----CACATCCGTGTTAGGGGCAGGGGGCCAAACCCAAGATCACTTTAAGCTCACCTCCCTTCCTGTGCTAA 1620
NC_001436 GTTCTCAAG-TAATACTCCCCTCAAAAA----CACATCCGTGTTAGGGGCAGGGGGCCAAACCCAAGATCACTTTAAGCTCACCTCCCTTCCTGTGCTAA 1620
AF074966 GTTCTCAAG-TAATACTCCCCTCAAAGA----CACATCCGTATTAGGGGCAGGAGGCCAAACCCAAGATCACTTTAAGCTCACCTCCCTTCCTGTGCTAA 1617
NC_000858 GTTCTCAAG-TAATACTCCCCTCAAAGA----CACATCCGTATTAGGGGCAGGAGGCCAAACCCAAGATCACTTTAAGCTCACCTCCCTTCCTGTGCTAA 1617
L02534 ATTCTCAAG-TGACACTCCCCTTAAAGA----CACATCCGTGTTAGGGGCAGGAGGCCAAACCCAAGACCACTTTAAGCTCACCTCCCTTCCTGTGCTAA 1625
Z46900 ATTCTCAAA-TAACACCCCCCTTAAAAA----CACATCTGTACTAGGGGCAGGAGGCCAAACTCAAGATCACTTTAAGCTCACCTCCCTTCCGGTGCTAA 1618
AY590142 GTTTCCGGA-AAATACCCCCCTTGAAAA----CACCGCAGTTGTAGGAGCAGGAGGCCAAACTCAAGATCAGTTTAAAATCGCCTCCCTTCCGGTGTTAA 1653
AF033818 GCTGGTTCGAGATTACCCAC------GGATCCCCGCCGCGGTGCTCGGAGCAGGGGGAGTCTCCCGGAACAGATACAATTGGCTACAAGGCCCTCTGAC-- 1585
NC_001414 GCTGGTTCGAGATTACCCAC------GGATCCCCGCCGCGGTGCTCGGAGCAGGGGGAGTCTCCCGGAACAGATACAATTGGCTACAAGGCCCTCTGAC-- 1585
K02120 GCTGGTTCGAGATTACCCAC------GGATCCCCGCCGCAGTGCTCGGAGCAGGGGGAGTCTCCCGGAACAGATACAATTGGCTACAAGGCCCTCTGAC-- 1582
D00647 GCTGGTTCGAGATTACCCAC------GGACCCCCGCCGCGGTACTCGGAGCGGGAGGAATCTCCCGGAACAGATACAATTGGCTACAAGGCCCTCTGAC-- 1582
AF257515 GCTGGTTCGAGAATACCCAC------GGACCCCCGCCGCAGTGCTCGGAGCGGGGGGAATCTCCCGGAACAGATACAATTGGTCACAAGGCCTTCTGAC-- 1585
ruler ......1710......1720......1730......1740......1750......1760......1770......1780......1790......1800

```
                 *  *  *    **  *     **  *        * **          *  * **           * * *   **     **  * **
   M10060 TATTCTTGCCCTT----CCGAAGGTCCCCCGTTATCCTTTCCTCCTG-----CCTCTTAGACACCCACAACAAATGGACCATCATT-GGAAGGGACGCCC 1698
NC_001488 TATTCTTGCCCTT----CCGAAGGTCCCCCGTTATCCTTTCCTCCTG-----CCTCTTAGACACCCACAACAAATGGACCATCATT-GGAAGGGACGCCC 1698
 AF412314 TATTCTTGCCCTT----CCGAAAGTCCCCCGTTATCCTTTCCTCCTG-----CCTCTTAGACACCCACAACAAATGGACCATCATT-GGAAGGGACGCCC 1698
 AF139382 TATTCTTGCCCTT----CCGAAAGTCCCCCGTTATCCTTTCCTCCTG-----CCTCTTAGACACCCACAACAAATGGACCATCATT-GGAAGGGACGCCC 1698
 AF326584 TATTCTTGCCCTT----CCGAAGGTCCCCCGTTATCCTTTCCTCCTG-----CCTCTTAGACACCCACAACAAATGGACCATCATT-GGAAGGGACGCCC 1698
 AF326583 TATTCTTGCCCTT----CCGAAAGTCCCCCGTTATCCTTTCCTCCTG-----CCTCTTAGACACCCACAACAAATGGACCATCATT-GGAAGGGACGCCC 1698
   L20734 TATTCTTACCCTT----CCGAAAGTCCCCCGTTATTCTTCCCTCCTG-----TCTCTTAGACACCCACAACAAATGGACCATCATT-GGAAGAGACGCCC 1698
   Y13051 TATTCTTACCCTT----CCGAAAGTCCCCCGTTATTCTTCCCTCCTG-----TCTCTTAGACACCCACAACAAATGGACCATCATT-GGAAGAGACGCCC 1698
   L11456 TATTCTTACCCTT----CCGAAAGTCCCCCGTTATTCTTCCCTCCTG-----TCTCTTAGACACCCACAACAAATGGACCATCATT-GGAAGAGACGCCC 1698
   X89270 TATTCTTACCCTT----CCGAAAGTCCCCCGTTATTCTTCCCTCCTG-----TCTCTTAGACACCCACAACAAATGGACCATCATT-GGAAGGGACGCCC 1698
 AF074965 TATTCTTACCCTT----CCGAAAGTCCCCCGTTATTCTTCCCTCCTG-----TCTCTTAGACACCCACAACAAATGGACCATCATT-GGAAGAGACGCCC 1633
   Y14365 TATTCTTACCCTT----CCGAAAGTCCCCCGTTATTCTTTCCTCCTG-----TCTCTTAGACACCCACAACAAATGGACCATCATT-GGGAGGGATGCCC 1698
   Y14570 TCTACCTACCCTT----CCGGAGGGCCCCTGTTACCCTCCCATCATG-----CCTTGTAGACACCAATAGCAAATGGGCCATTATT-GGCCGTGATATCC 1686
NC_001815 TCTACCTACCCTT----CCGGAGGGCCCCTGTTACCCTCCCATCATG-----CCTTGTAGACACCAATAGCAAATGGGCCATTATT-GGCCGTGATATCC 1686
   U90557 TCTACCTGCCCTT----CCGGAAGGCCCCCGTTACTCTCCCGTCATG-----CCTTCTAGACACCGATAATAAATGGGCCATTATT-GGCCGTGATATCC 1683
   Y07616 TCCATTTGCCCTT----CCGAAGGCAGCCAGTAACCTTAACTTCTTG-----CCTAATCGACACCAACAACCAATGGGCCAT-ATTAGGACGAGATGCCC 1669
 AY217650 TCCATTTGCCCTT----CCGAAGGCAGCCAGTAACCCTAACTTCTTG-----CCTAATCGACACCAACAACCAGTGGGCCAT-ATTAGGACGAGATGCCT 1666
 AF517775 TCCATTTGCCTTT----CCGAAGACAGCCAGTGACCCTGACCTCTTG-----TCTAATCGATGTTAACAACCAGTGGACCAT-ATTAGGGCGGGACGCCC 1665
 AY222339 TCCACTTGCCCTT----CCGAAGACAGCCGGTGACCCTAACCTCTTG-----CCTAATCGATATTAACAACCAATGGACCAT-ATTAGGACGGGACGCCC 1665
NC_003323 TCCACTTGCCCTT----CCGGAAACGGCCAGTAACCCTAACTTCCTG-----CCTAATTGATACTAACGATCAGTGGACTAT-ACTAGGACGGGATGCTC 1666
   L36905 TACGCCTCCCTTT----CCGGACAACGCCTATTGTTTTAACATCTTG-----CCTAGTTGATACCAAAAACAACTGGGCCATCATA-GGTCGTGATGCCT 1710
 AF139170 TACGCCTCCCTTT----CCGGACAACGCCTATTGTTTTAACATCTTG-----CCTAGTTGATACCAAAAACAACTGGGCCATCATA-GGTCGTGATGCCT 1698
 AF042071 TACGCCTCCCTTT----CCGGACAACGCCTATTATTTTAACATCGTG-----CCTAGTTGATACCAAAAACAACTGGGCCATCATA-GGTCGTGATGCCT 1710
   L03561 TACGCCTCCCTTT----CCGGACAACGCCTATTGTTTTAACATCTTG-----CCTAGTTGATACCAAAAACAACTGGGCCATCATA-GGTCGTGATGCCT 1710
 AF259264 TACGCCTCCCTTT----CCGGACAACGCCTATTGTTTTAACATCTTG-----CCTAGTTGATACCAAAAACAACTGGGCCATCATA-GGTCGCGATGCCT 1710
 AY563953 TACGCCTCCCTTT----CCGGACAACGCCTATTGTTTTAACATCTTG-----CCTAGTTGATACCAAAAACAACTGGGCCATCATA-GGTCGTGATGCCT 1710
 AY563954 TACGCCTCCCTTT----CCGGACAACGCCTATTGTTTTAACATCTTG-----CCTAGTTGATACCAAAAACAACTGGGCCATCATA-GGTCGTGATGCCT 1710
   U19949 TACGCCTCCCTTT----CCGGACAACGCCTATTGTTTTAACATCTTG-----CCTAGTTGATACCAAAAACAACTGGGCCATCATA-GGTCGTGATGCCT 1710
   J02029 TACGCCTCCCTTT----CCGGACAACGCCTATTGTTTTAACATCTTG-----CCTAGTTGATACCAAAAACAACTAGGCCATCATA-GGTCGTGATGCCT 1710
   D13748 TACGCCTCCCTTT----CCGGACGACGCCTATTGTTTTAACATCTTG-----CCTAGTTGATACCAAAAACAACTGGGCCATCATA-GGTCGTGATGCCT 1710
 AF033817 TACGCCTCCCTTT----CCGGACGACGCCTATTGTTTTAACATCTTG-----CCTAGTTGATACCAAAAACAACTGGGCCATCATA-GGTCGTGATGCCT 1710
NC_001436 TACGCCTCCCTTT----CCGGACGACGCCTATTGTTTTAACATCTTG-----CCTAGTTGATACCAAAAACAACTGGGCCATCATA-GGTCGTGATGCCT 1710
 AF074966 TACGCCTCCCTTT----CCGGACAACGCCTATTGTTTTAACATCTTG-----CCTAGTTGATACCGAAAACAACTGGGCCATCATA-GGTCGTGATGCCT 1707
NC_000858 TACGCCTCCCTTT----CCGGACAACGCCTATTGTTTTAACATCTTG-----CCTAGTTGATACCGAAAACAACTGGGCCATCATA-GGTCGTGATGCCT 1707
   L02534 TACGCCTCCCTTT----CCGGACAACGCCTATTGTTTTAACATCTTG-----CTTAGTCGATACCAAAAACAACTGGGCCATCATA-GGTCGTGATGCCT 1715
   Z46900 TACGCCTCCCTTT----CCGGACAACGCCTATTGTTTTAACATCTTG-----CCTAGTCGATACTAAAAACAACTGGGCCATCATA-GGTCGTGACGCCT 1708
 AY590142 TTCACCTCCCTTT----CCGGACGACACCCATTGTGTTAACCTCATG-----CCTTATAGATACCGAAAACAACTGGGTCATAATT-GGCCGCGACGCCC 1743
 AF033818 ----CCTGGCTCTAAAACCAGAGGGTCCCTTTA---TCACCATCCCAAAAATTTTAGTTGACACTTTCGATAAATGGCAAATTTTA-GGACGGGACGTCC 1677
NC_001414 ----CCTGGCTCTAAAACCAGAGGGTCCCTTTA---TCACCATCCCAAAAATTTTAGTTGACACTTTCGATAAATGGCAAATTTTA-GGACGGGACGTCC 1677
   K02120 ----CCTGGCTCTAAAACCAGAGGGTCCCTTTA---TCACCATCCCAAAAATTTTAGTTGACACTTCCGACAAATGGCAAATTTTA-GGACGGGACGTCC 1674
   D00647 ----CCTGGCTCTAAAACCAGAGGGTCCCTTTA---TCACCATCCCAAAAATTTTAGTTGACACCTTCGATAAATGGCAAATTTTA-GGACGGGACGTCT 1674
 AF257515 ----CCTGGCTATAAAACCAGAGGGTCCCTTTA---TCACCATCCCAAAAATTTTAGTTGACACCTTCGACAAATGGCAAATTTTA-GGACGGGACGCCT 1677
    ruler ......1810......1820......1830......1840......1850......1860......1870......1880......1890......1900
```

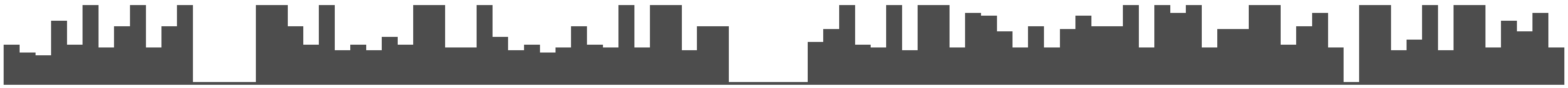

```
                    * * *  *    ** *  *  *  * **                            *   *                             *
M10060     TACAACAATGCC-AGGGGCTTCTATACC--TCCCAGACGAC-------------------CCCAGCCCCC-----------------ACCAAT-TGC--T  1756
NC_001488  TACAACAATGCC-AGGGGCTTCTATACC--TCCCAGACGAC-------------------CCCAGCCCCC-----------------ACCAAT-TGC--T  1756
AF412314   TACAACAATGCC-AGGGGCTTCTATACC--TCCCAGACGAC-------------------CCCAGCCCCC-----------------ACCAAT-TGC--T  1756
AF139382   TACAACAATGCC-AGGGGCTTCTATACC--TTCCAGACGAC-------------------CCCAGCCCCC-----------------ACCAAT-TGC--T  1756
AF326584   TACAACAATGCC-AGGGGCTTCTATACC--TTCCAGACGAC-------------------CCCAGCCCCC-----------------ACCAAT-TGC--T  1756
AF326583   TACAACAATGCC-AGGGGCTTCTATACC--TTCCAGACGAC-------------------CCCAGCCCCC-----------------ACCAAT-TGC--T  1756
L20734     TACAACAATGCC-AGGGGCTTCTATACC--TTCCAGACGAT-------------------CCCAGCCCCC-----------------ATCAAT-TGC--T  1756
Y13051     TACAACAATGCC-AGGGGCTTCTATACC--TTCCAGACGAT-------------------CCCAGCCCCC-----------------ATCAAT-TGC--T  1756
L11456     TACAACAATGCC-AGGGGCTTCTATACC--TTCCAGACGAT-------------------CCCAGCCCCC-----------------ACCAAT-TGC--T  1756
X89270     TACAACAATGCC-AGGGGCTTCTATACC--TTCCAGACGAT-------------------CCCAGCCCCC-----------------ACCAAT-TGC--T  1756
AF074965   TACAACAATGCC-AGGGGCTTCTATACC--TTCCAGACGAT-------------------CCCAGCCCCC-----------------ACCAAT-TGC--T  1691
Y14365     TGCAACAATGCC-AGGGACTTCTATACC--TCCCAGACGAT-------------------CCCAGCCCCCGGT--------GCTCCCACCAAT-TGC--T  1765
Y14570     TCCAGAAATGCC-AGAGTGTCCTGTACC--TTCCGGAGGACAATCTGTGCAAGGGTACCCCCCGGCTCCCCGATGGAATAACTCCTCCCCGAT-TAT--T  1780
NC_001815  TCCAGAAATGCC-AGAGTGTCCTGTACC--TTCCGGAGGACAATCTGTGCAAGGGTACCCCCCGGCTCCCCGATGGAATAACTCCTCCCCGAT-TAT--T  1780
U90557     TCCAGCAATGCC-AGAGTGTCCTGTACC--TCCCGGAGGACAATCTCTGCGAAGGTACCCCCCGGCCCTCCGGCAGAATGAATTCCCCCCGAC-TAT--T  1777
Y07616     TACAACAATGTC-AGAGTTCCCTTTATC--TTGCTGA------------------------CCAACCCCC-------------------GGAGGTCC--T  1721
AY217650   TACAACAATGTC-AGAGTTCCCTCTATC--TTGCTGA------------------------CCAACCCCC-------------------GGAGATCC--T  1718
AF517775   TACAACAGTGTC-AGGGCTCCCTCTATC--TGGCTGA------------------------CCAGCCCTC-------------------GAGTGTCC--T  1717
AY222339   TACAACAGTGTC-AGGGCTCCCTCTATC--TGGCCGA------------------------CCAACCCTC-------------------AAATGTCC--T  1717
NC_003323  TGCAACAGTGTC-AAAGCTCCCTCTACC--TTGCTGA------------------------CCAACCCTC-------------------AAGTGTTC--T  1718
L36905     TACAACAATGCC-AAGGCGCCCTGTACC--TCCCTGAGGCAAA------------------AGGGCCGCC-----------------TGTAAT---CT-T  1768
AF139170   TACAACAATGCC-AAGGCGTCCTGTACC--TCCCTGAGGCAAA------------------AGGGCCGCC-----------------TGTAAT---CT-T  1756
AF042071   TACAACAATGCC-AAGGCGTCCTGTACC--TCCCTGAGGCAAA------------------AGGGCCGCC-----------------TGTAAT---CT-T  1768
L03561     TACAACAATGCC-AGGGCGTCCTGTACC--TCCCTGAGGCAAA------------------AGGGCCGCC-----------------TGTAAT---CT-T  1768
AF259264   TACAACAATGCC-AGGGCGTCCTGTACC--TCCCTGAGGCAAA------------------AGGGCCGCC-----------------TGTAAT---CT-T  1768
AY563953   TACAACAATGCC-AAGGCGTCCTGTACC--TCCCTGAGGCAAA------------------AGGGCCGCC-----------------TGTTAT---CT-T  1768
AY563954   TACAACAATGCC-AAGGCGTCCTGTACC--TCCCTGAGGCAAA------------------AGGGCCGCC-----------------TGTTAT---CT-T  1768
U19949     TACAACAATGCC-AAGGCGTCCTGTACC--TCCCTGAGGCAAA------------------AAGGCCGCC-----------------TGTAAT---CT-T  1768
J02029     TACAACAATGCC-AAGGCGTCCTGTACC--TCCCTGAGGCAAA------------------AAGGCCGCC-----------------TGTAAT---CT-T  1768
D13748     TACAACAATGCC-AAGGCGTCCTGTACC--TCCCTGAGGCAAA------------------AAGGCCGCC-----------------TGTAAT---CT-T  1768
AF033817   TACAACAATGCC-AAGGCGTCCTGTACC--TCCCTGAGGCAAA------------------AAGGCCGCC-----------------TGTAAT---CT-T  1768
NC_001436  TACAACAATGCC-AAGGCGTCCTGTACC--TCCCTGAGGCAAA------------------AAGGCCGCC-----------------TGTAAT---CT-T  1768
AF074966   TACAACAATGCC-AAGGCGTCCTGTACC--TCCCTGAGGCAAA------------------AGGGCCGCC-----------------AGTAAT---CC-T  1765
NC_000858  TACAACAATGCC-AAGGCGTCCTGTACC--TCCCTGAGGCAAA------------------AGGGCCGCC-----------------AGTAAT---CC-T  1765
L02534     TACAGCAATGCC-AAGGCGTTCTGTATC--TTCCTGAGGCAAA------------------AAGGCCGCC-----------------TGTAAT--TCT-T  1774
Z46900     TACAACAATGCC-AAGGCGTCCTATATC--TTCCCGAGGCAAA------------------GGGGCCGTC-----------------TGTCAT---CT-T  1766
AY590142   TACAGCAATGCC-AAGGGGCCCTATACC--TCCCTGAGGCAGG------------------TCGCCCACC-----------------ACTAAT--AC--T  1801
AF033818   TCTCCC---GCCTACAGGCCTCTATCTCCATACCTGAGGAGGT------------------ACGCCCCCC-----------------CATGGTAGGCG-T  1738
NC_001414  TCTCCC---GCCTACAGGCCTCTATCTCCATACCTGAGGAGGT------------------ACGCCCCCC-----------------CATGGTAGGCG-T  1738
K02120     -CTCCC---GCCTACAGGCTTCTATCTCCATACCTGAGGAAGT------------------ACGCCCCCC-----------------TGTGGTAGGCG-T  1734
D00647     -CTCCC---GCCTACAGGCTTCTATCTCCATACCTGAGGAAGT------------------ACACCCCCC-----------------TGTGGTAGGCG-T  1734
AF257515   -CTCCC---GGCTACAGGCTTCTATCTCCATACCTGAGGAAGT------------------ACCCCCCCC-----------------TGTGGTAGGCG-T  1737
ruler      ......1910......1920......1930......1940......1950......1960......1970......1980......1990......2000
```

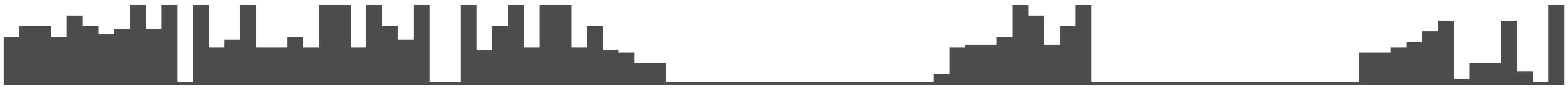

```
                *       *  *        * **  * ** **  * **    ** **  *  *    ** **  *  ******  ** ** ********  * 
   M10060 GCCAA-T--AGCCACTCCAAACACCATAGGCCTCGAACACCTTCCCCCACCTCCCCAAGTGGACCAATTTCC-TTTAAACCTGAGCGCCTCCAGGCCTTA  1852
NC_001488 GCCAA-T--AGCCACTCCAAACACCATAGGCCTCGAACACCTTCCCCCACCTCCCCAAGTGGACCAATTTCC-TTTAAACCTGAGCGCCTCCAGGCCTTA  1852
 AF412314 GCCAA-T--AGCCACTCCAAACACCATAGGCCTCGAACACCTTCCCCCACCTCCCCAAGTGGACCAATTTCC-TTTAAACCTGAGCGCCTCCAGGCCTTA  1852
 AF139382 GCCAA-T--AGCCACCCCAAACACCATAGGCCTCGAACACCTTCCCCCACCTCCCCAAGTGGACCAATTTCC-TTTAAACCTGAGCGCCTCCAGGCCTTA  1852
 AF326584 GCCAA-T--AGCCACCCCAAACACCATAGGCCTCGAACACCTTCCCCCACCTCCCCAAGTGGACCAATTTTC-TTTAAACCTGAGCGCCTCCAGGCCTTA  1852
 AF326583 GCCAA-T--AGCCACCCCAAACACCATAGGCCTCGAACACCTTCCCCCACCTCCCCAAGTGGACCAATTTCC-TTTAAACCTGAGCGCCTCCAGGCCTTA  1852
   L20734 GCCAA-T--AGCCACTCCACACACCATAGGCCTCGAACACCTTCCCCCACCGCCCCAGGTGGACCAATTTCC-TTTAAACCTGAGCGCCTCCAGGCCTTA  1852
   Y13051 GCCAA-T--AGCCACTCCACACACCATAGGCCTCGAACACCTTCCCCCACCGCCCCAGGTGGACCAATTTCC-TTTAAACTTGAGCGCCTCCAGGCCTTA  1852
   L11456 GCCAA-T--AGCCACTCCACACACCATAGGCCTGGAACACCTTCCCCCACCGCCCCAGGTGGACCAATTTCC-TTTAAACCTGAGCGCCTCCAGGCCTTA  1852
   X89270 GCCAA-T--AGCCACTCCACACACCATAGGCCTGGAACACCTTCCCCCACCGCCCCAGGTGGACCAATTTCC-TTTAAACCTGAGCGCCTCCAGGCCTTA  1852
 AF074965 GCCAA-T--ATCCACTCCACACCCCATAGGCTTCGAACACCTTCCCCCACCGCCGCAGGTGGACCAATTTCC-TTTAAACGTGAGCGCCTCCAGGCCTTA  1787
   Y14365 GCCAA-T--AGCCACTCCAAACACCATAGGCCTCGAACACCTTCCCCCACCTCCCCAGGTGGACCAATTTCC-TTTAAACCTGAGCGCCTCCAGGCCTTA  1861
   Y14570 ACCCG-T--GACCACCCCCAATGTCATTGGCCTTGAGCACTTCCCACCACCCCCACAGATAGATCAGTTCCC-TTTAAACCTGAGCGCCTCCAGGCCTTG  1876
NC_001815 ACCCG-T--GACCACCCCCAATGTCATTGGCCTTGAGCACTTCCCACCACCCCCACAGATAGATCAGTTCCC-TTTAAACCTGAGCGCCTCCAGGCCTTG  1876
   U90557 ACCCG-T--GGCCACCCCCAGTGTCATCGGCCTTGAGCACTTCCCACCACCCCCACAGATAGATCAGTTCCCCTTTAAACCTGAGCGCCTCCAGGCCTTG  1874
   Y07616 CCCCG-TTCAAACAC-CCAAGC-TCATTGGGCTAGAACACCTGCCTCCGCCCCCGGAAATCTCGCAGTTCCC-GTTAAACCGGAACGCCTCCAGGCCTTG  1817
 AY217650 CCCCG-TTCAAACAC-CCAAGC-TTATTGGTTTAGAGCACCTGCCCCCGCCCCCGGAGGTCTCGCAGTTCCC-GTTAAACCAGAACGCCTCCAGGCCCTG  1814
 AF517775 CCCTG-TTCAGACAC-CCAAGC-TTATCGGATTAGAACACCTTCCCCCGCCCCCGGAAGTCTCTCAGTTTCC-GTTAAACCGGAGCGCCTCCAGGCTCTG  1813
 AY222339 TCCTG-TCCAAACAC-CCAAAC-TTATTGGATTAGAACACCTTCCCCCGCCCCCAGAAGTTTCTCAGTTCCC-GTTAAACCGGAGCGCCTCCAGGCCCTG  1813
NC_003323 CCCTG-TCCAAACAC-CCAAGC-TTATTGGGTTAGAACATCTTCCCCCGCCCCCGGAGGTCTCGCAGTTCCC-GTTAAACCGGAGCGCCTCCAGGCCCTG  1814
   L36905 GCCAA-TACAGGCGC--CAGCCGTCCTTGGGCTAGAACACCTCCCAAGGCCCCCCGAAATCAGCCAGTTCCC-TTTAAACCAGAACGGCTCCAGGCCTTG  1864
 AF139170 GCCAA-TACAGGCGC--CAGCCGTCCTTGGGCTAGAACACCTCCCAAGGCCCCCCGAAATCAGCCAGTTCCC-TTTAAACCAGAACGCCTCCAGGCCTTG  1852
 AF042071 GCCAA-TACAGGCGC--CAGCCGTCCTTGGGCTAGAACATCTCCCAAGGCCCCCCGAAATCAGCCAGTTCCC-TTTAAACCAGAACGCCTCCAGGCCTTG  1864
   L03561 GCCAA-TACAGGCGC--CAGCCGTCCTTGGACTAGAACACCTCCCAAGGCCCCCCGAAATCAGCCAGTTCCC-TTTAAACCAGAACGCCTCCAGGCCTTG  1864
 AF259264 GCCAA-TACAGGCGC--CAGCCGTCCTTGGGCTAGAACACCTCCCAAGGCCCCCCGAAATTAGCCAGTTCCC-TTTAAACCAGAACGCCTCCAGGCCTTG  1864
 AY563953 GCCAA-TACAGGCGC--CAGCCGTCCTTGGGCTAGAACACCTCCCAAGGCCCCCCGAAATCAGCCAGTTCCC-TTTAAACCAGAACGCCTCCAGGCCTTG  1864
 AY563954 GCCAA-TACAGGCGC--CAGCCGTCCTTGGGCTAGAACACCTCCCAAGGCCCCCCGAAATCAGCCAGTTCCC-TTTAAACCAGAACGCCTCCAGGCCTTG  1864
   U19949 GCCAA-TACAGGCGC--CAGCCGTCCTTGGGCTAGAACACCTCCCAAGGCCCCCCGAAATCAGCCAGTTCCC-TTTAAACCAGAACGCCTCCAGGCCTTG  1864
   J02029 GCCAA-TACAGGCGC--CAGCCGTCCTTGGGCTAGAACACCTCCCAAGGCCCCCCGAAATCAGCCAGTTCCC-TTTAAACCAGAACGCCTCCAGGCCTTG  1864
   D13748 GCCAA-TACAGGCGC--CAGCTGTCCTTGGGCTAGAACACCTCCCAAGGCCCCCCGAAATCAGCCAGTTCCC-TTTAAACCAGAACGCCTCCAGGCCTTG  1864
 AF033817 GCCAA-TACAGGCGC--CAGCTGTCCTTGGGCTAGAACACCTCCCAAGGCCCCCCGAAATCAGCCAGTTCCC-TTTAAACCAGAACGCCTCCAGGCCTTG  1864
NC_001436 GCCAA-TACAGGCGC--CAGCTGTCCTTGGGCTAGAACACCTCCCAAGGCCCCCCGAAATCAGCCAGTTCCC-TTTAAACCAGAACGCCTCCAGGCCTTG  1864
 AF074966 GCCTA-TACAGGTGC--CAGCCGTCCTTGGGCTAGAACACCTCCCAAGGCCACCTGAAATCAGCCAGTTCCC-TTTAAACCAGAACGCCTCCAGGCCTTG  1861
NC_000858 GCCTA-TACAGGTGC--CAGCCGTCCTTGGGCTAGAACACCTCCCAAGGCCACCTGAAATCAGCCAGTTCCC-TTTAAACCAGAACGCCTCCAGGCCTTG  1861
   L02534 GCCAA-TACAGGCGC--CAGCCGTCCTTGGACTAGAACACCTCCCAAGGCCCCCAGAAATTAGCCAGTTCCC-TTTAAACCAGAACGCCTCCAGGCCCTG  1870
   Z46900 ACCAA-TACAGACGC--CAGCCGTCCTTGGGCTAGAGCACCTCCCGAAGCCCCCCGAAATTGGCCAGTTCCC-TTTAAACCGGAACGCCTCCAGGCCTTG  1862
 AY590142 GCCAG-TTCAGGCGC--CGGCCATGCTAGGCCTCGAGCATTTCCCAAAACCCCCCGAAATCAGCCAGTTTCC-TTTAAACCTGAACGCCTCCAGGCCCTG  1897
 AF033818 CCTAGAT---GCCCCCCCGAGCCACATTGGATTAGAACATTTGCCCGTCCCACCTGAGGTACCTCAATTCCC-TTTAAACTAGAACGCCTCCAGGCCCTT  1834
NC_001414 CCTAGAT---GCCCCCCCGAGCCACATTGGATTAGAACATTTGCCCGTCCCACCTGAGGTACCTCAATTCCC-TTTAAACTAGAACGCCTCCAGGCCCTT  1834
   K02120 CTTGGAT---ACCCCCCCGAGCCACATTGGATTAGAACATCTGCCCCCCCCACCTGAGGTGCCTCAATTCCC-TTTAAACTAGAACGCCTCCAGGCCCTT  1830
   D00647 CTTAGAT---GCCCCCCCGAGCCACATTGGATTAGAACATTTGCCCCCCCCACCTGAGGTACCTCAATTCCC-TTTAAACTAGAACGCCTCCAGGCCCTT  1830
 AF257515 CTTGGAT---GCCCCCCCGAGCCACATTGGATTAGAACATCTGCCCCCCCCCCCTGAGGTACCTCAATTCCC-TTTAAACTAGAACGCCTCCAGGCCCTT  1833
    ruler ......2010......2020......2030......2040......2050......2060......2070......2080......2090......2100
```

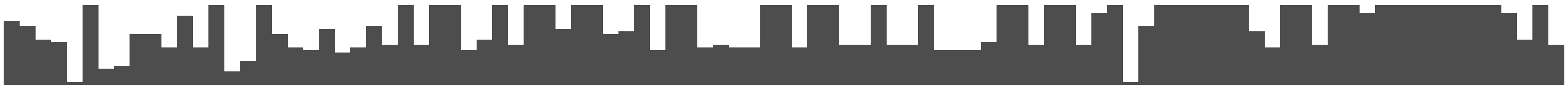

Supplement: Additional File 1 — contains an alignment of the 42 retrovirus sequences from Table 1 (the genome region between primer delta-F and delta-R). [file 1742-4690-4-11-S1.pdf]
